# New miRNA Profiles Accurately Distinguish Renal Cell Carcinomas and Upper Tract Urothelial Carcinomas from the Normal Kidney

**DOI:** 10.1371/journal.pone.0091646

**Published:** 2014-03-12

**Authors:** Apostolos Zaravinos, George I. Lambrou, Nikos Mourmouras, Patroklos Katafygiotis, Gregory Papagregoriou, Krinio Giannikou, Dimitris Delakas, Constantinos Deltas

**Affiliations:** 1 Molecular Medicine Research Center and Laboratory of Molecular and Medical Genetics, Department of Biological Sciences, University of Cyprus, Nicosia, Cyprus; 2 Choremeio Research Laboratory, First Department of Pediatrics, University of Athens, Athens, Greece; 3 Department of Urology, Asklipieio General Hospital, Athens, Greece; 4 Department of Medical Genetics, Medical School, University of Athens, Athens, Greece; The Chinese University of Hong Kong, Hong Kong

## Abstract

**Background:**

Upper tract urothelial carcinomas (UT-UC) can invade the pelvicalyceal system making differential diagnosis of the various histologically distinct renal cell carcinoma (RCC) subtypes and UT-UC, difficult. Correct diagnosis is critical for determining appropriate surgery and post-surgical treatments. We aimed to identify microRNA (miRNA) signatures that can accurately distinguish the most prevalent RCC subtypes and UT-UC form the normal kidney.

**Methods and Findings:**

miRNA profiling was performed on FFPE tissue sections from RCC and UT-UC and normal kidney and 434 miRNAs were significantly deregulated in cancerous vs. the normal tissue. Hierarchical clustering distinguished UT-UCs from RCCs and classified the various RCC subtypes among them. qRT-PCR validated the deregulated expression profile for the majority of the miRNAs and ROC analysis revealed their capability to discriminate between tumour and normal kidney. An independent cohort of freshly frozen RCC and UT-UC samples was used to validate the deregulated miRNAs with the best discriminatory ability (AUC>0.8, p<0.001). Many of them were located within cytogenetic regions that were previously reported to be significantly aberrated. miRNA targets were predicted using the miRWalk algorithm and ingenuity pathway analysis identified the canonical pathways and curated networks of the deregulated miRNAs. Using the miRWalk algorithm, we further identified the top anti-correlated mRNA/miRNA pairs, between the deregulated miRNAs from our study and the top co-deregulated mRNAs among 5 independent ccRCC GEO datasets. The AB8/13 undifferentiated podocyte cells were used for functional assays using luciferase reporter constructs and the developmental transcription factor TFCP2L1 was proved to be a true target of miR-489, which was the second most upregulated miRNA in ccRCC.

**Conclusions:**

We identified novel miRNAs specific for each RCC subtype and UT-UC, we investigated their putative targets, the networks and pathways in which they participate and we functionally verified the true targets of the top deregulated miRNAs.

## Introduction

Renal cell carcinoma (RCC) represents 2–3% of all cancers and accounts for approximately 90% of all kidney malignancies. Apart from surgery, it is both chemotherapy and radiotherapy resistant and it is composed of various morphologically and cytogenetically distinct subtypes. The most prevalent subtypes are clear cell RCC (ccRCC, 75–80%), papillary RCC (papRCC, 10–15%) and chromophobe RCC (chRCC, 5%) [1]. Distinguishing RCC subtypes is of clinical importance because they have different prognoses and consequently different management schemes [2]. However, morphology-based distinction is not always conclusive since some subtypes may have overlapping or related morphologic features. For the purpose of targeted therapy it is especially important to classify the different subtypes of RCC. The histological types arise from different cells of origin in the kidney, different constellations of genetic alterations [3], and expression or mutation in different oncogenic pathways. Therefore, different subtypes offer different molecular candidates for targeted therapy, such as Tyrosine Kinase Inhibitors, Sorafenib and Sunitinib, mTOR inhibitors, Everolimus and Temsirolimus, etc. There is growing evidence that variability in response rates may be linked to sub-classification [4]. Therefore, new biomarkers are needed in order to improve the identification and diagnosis of renal tumor subtypes. Recent data suggest that RCC classification through microRNA (miRNA) expression profiles is highly accurate [5], [6].

Upper tract urothelial carcinoma (UT-UC) is a relatively uncommon form of kidney cancer arising from the urothelial lining of the renal pelvis and calyces. UT-UC accounts for the majority of bladder cancer; however, it only accounts for about 7% of renal neoplasms [7]. UC of the renal pelvis is an aggressive tumour, which may invade the renal parenchyma, mimicking primary renal cell carcinoma. Similarly, advanced RCC can invade the pelvicalyceal system. This can make differential diagnosis of RCC and urothelial carcinoma of the renal pelvis difficult. Correct diagnosis is critical for determining appropriate surgery and post-surgical treatments. For instance, UT-UC including renal pelvis, calyces and ureters will require radical nephrectomy with ureterectomy and bladder cuff resection. However, RCC will require only partial or radical nephrectomy without extensive ureter resection. Correct diagnosis is critical for determining appropriate surgery and post-surgical treatments. Therefore, it is of major importance to identify biomarkers that can accurately distinguish UT-UC from RCC [8], [9].

MicroRNAs (miRNAs) are small non-coding RNAs of approximately 19–23 nt size, shown to regulate gene expression at the post-transcriptional level, by binding through partial sequence homology to the 3′ UTR of mammalian target mRNAs and causing translational inhibition and/or mRNA degradation [10]. miRNAs have been identified as key regulators in many biological processes including development, differentiation, apoptosis and proliferation. They are aberrantly expressed or mutated in cancers, suggesting that they may play a role as a novel class of oncogenes or tumour suppressor genes [11]. Deregulated miRNAs have been found in many tumours, including kidney cancer and carcinomas of the urinary tract [5], [12], [13], [14], [15], [16], [17], [18], [19], [20], [21]; however, not much is known regarding their expression profile in upper tract urothelial carcinomas. Furthermore, in RCC the results are not always consistent [12], [13], [17], [18], [22], [23], [24], [25]. Additionally, many deregulated miRNAs are located in chromosomal regions that are frequently altered in cancerous situations [26].

To our knowledge there are no accurate biomarkers for early detection of both RCC and UT-UC, thereby accounting for late diagnosis and subsequent poor prognosis. Better understanding of RCC and UT-UC pathogenesis is required for identifying biomarkers and developing novel targeted therapies. In the present study, we examined the miRNA expression profiles mainly of ccRCC, as well as in a smaller cohort of papRCC, chRCC and UT-UC samples. We identified novel miRNAs that are specific for each tumour entity, we investigated their putative targets, and we explored the networks and pathways in which these miRNAs participate. Finally, we functionally verified the target gene of the top deregulated miRNA in ccRCC.

## Materials and Methods

### Patient samples

**Figure 1:**
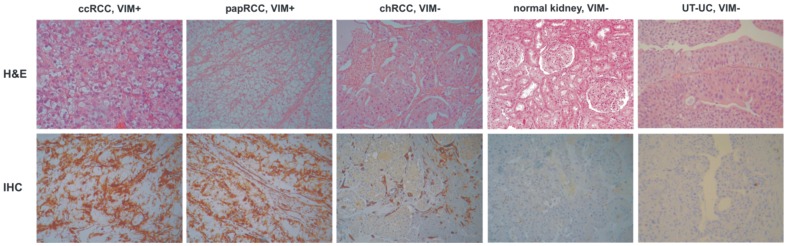
Haematoxylin and eosin (H&E) staining and immunohistochemistry (IHC). *Upper pannel:* Representative H&E staining from ccRCC, papRCC, chRCC and normal kidney tissue. About 5–10 serial tissue sections of 10 μm were cut from each FFPE block, deparaffinized with xylene, hydrated and stained with H&E before microscopic examination. When the proportion of tumour cells was >70% the FFPE block was subjected to total RNA extraction. *Lower pannel:* IHC of FFPE tissue sections using anti-vimentin as primary antibody. Vimentin was predominantly seen in ccRCC and papRCC (∼70% and ∼50%, respectively), but only rarely in chRCC (4%) and absent in the normal kidney. Vimentin was also down-regulated in the majority of UT-UC cases.

Formalin-fixed and paraffin embedded (FFPE) tissue samples from patients with kidney cancer and upper tract urothelial carcinoma were retrieved from the archives of the Asklipieio General Hospital, Athens, following research ethics board approval from the same Hospital. Paraffin sections from each specimen were reviewed by two independent pathologists and histologically classified according to the WHO classification and staged according to the UICC-TNM classification (2009). Nuclear grade was scored according to the Fuhrman classification system. Kidney specimens were collected between 2007 and 2011. Written informed consent was obtained from all patients included in this study. The study protocol was approved by the Ethics Committees of the Asklipieio General Hospital, Athens and the University of Cyprus. Twenty-four ccRCC, 5 papRCC, 3 chRCC, 5 UT-UC patients and 40 normal kidney tissue (parenchyma/cortex) samples which were used as controls, were enrolled in the present study (**Table 1**). None of the patients had a history of previous bladder cancer or concomitant bladder cancer at UT-UC diagnosis. The criteria to select normal kidney tissues were that the normal kidney cortex tissue was from the same individual, from the same kidney, from the same surgical specimen, and collected under the same standard operation procedure conditions: if possible the sampling of normal cortex was done leaving ∼2 cm of macroscopically healthy cortex adjacent to the tumor untouched. ccRCC are macroscopically characterized by a sharp demarcation (fibrous pseudocapsule) between tumor and surrounding non-tumoral tissue. This is also reflected microscopically, as RCC are sharply demarcated from the surrounding tissue. The non-tumoral tissue was therefore obtained in macroscopically clear-cut benign tissue. Haematoxylin and eosin (H&E) staining and immunohistochemistry (IHC) for several markers (AE1/AE3 keratins, CK7, CK8, CK18, CK19, EMA, CD10, Vimentin, EGFR, Ki67, NSE, p53 and S-100) was performed for all RCC samples. Anti-vimentin staining was also performed for UT-UC samples (**Figure 1**).

**Table 1:** Clinicopathological information of the patients.

	ccRCC (n = 24)	papRCC (n = 5)	chRCC (n = 3)	UT-UC (n = 5)
**Median age (range), y**	70 (43–79)	67 (53–80)	68 (60–77)	76 (76–78)
**Gender (male/female)**	20/4	4/1	0/3	4/1
**Stage**				
T1	13	4	0	3
T2	6	1	3	1
T3	5	0	0	1
T4	0	0	0	0
**Metastatic tissue samples**	2	0	0	0
**Grade**				
1	0	0	0	0
2	5 (T1a), 3 (T1b), 2 (T2a), 1 (T2), 1 (T3a)	2 (T1a)	1 (T2b)	1 (T1+in situ)
3	3 (T1a), 2 (T1b), 1 (T2a), 3 (T3a)	2 (T1b), 1 (T2a)	2 (T2a)	1 (T1), 1 (T2), 1 (T3)
4	1 (T2a), 1 (T2b), 1 (T3a)	0	0	0
**median tumour size (range), cm**				
Grade 1	-	-	-	-
Grade 2	4 (2–8.5)	2.75 (2.5–3)	13.2 (0)	1.5 (0)
Grade 3	6.5 (1.5–9)	5.5 (4.5–8.5)	9.25 (8.5–10)	0.7 (0.5–1.5)
Grade 4	8.4 (6.5–13)	-	-	-
**Nodal status,**				
positive/negative/unknown	0/24/0	0/5/0	0/3/0	0/5/0
**Metastatic status,**				
positive/negative	2/22	0/5	0/3	0/5

### RNA extraction and microRNA microarray analysis

Total RNA containing small RNAs was isolated from 18 ccRCC, 5 papRCC, 3 chRCC, 4 UT-UC and 20 normal tissue samples using the High Pure FFPE RNA Micro Kit (Roche Applied Science, IN). The quality of the total RNA was verified by an Agilent 2100 Bioanalyzer (Agilent Technologies, Santa Clara, CA) and the concentration was measured spectrophotometrically (Nanodrop technologies, Montchanin, DE). Tissue sections were deparaffinized with xylene and ethanol washes, treated with protease and total RNA containing small RNAs was isolated using the High Pure FFPE RNA Micro Kit (Roche Applied Science). Total RNA (0.5 μg) from each sample and reference was labeled with Hy3 fluorescent label, using the miRCURY LNA microRNA Hi-Power Labeling Kit (Exiqon, Vedbaek, Denmark), according to the manufacturer protocol. The Hy3-labeled samples were hybridized to the miRCURY LNA microRNA Array, 6^th^ gen (Exiqon, Vedbaek, Denmark), containing capture probes that target all miRNAs for all species registered in the miRBase v18.0. The hybridization was performed using an Agilent hybridization SureHyb chamber and gasket slide kits. After hybridization, the microarray slides were scanned at 10 μm using the High-Resolution Microarray Scanner (Agilent Technologies) and stored in an ozone free environment. The image analysis was carried out using the ImaGene 8.0 software (BioDiscovery, Inc., USA). Filtering was performed based on the signal intensity. Spots with no signal above the background (flags 1 and 2) were detected and removed. Background correction was performed by subtracting the median global background from the median local background from the signal intensity. Normalization was performed using the quantile normalization algorithm. Normalized data were further extracted, pre-processed and sorted with Microsoft Excel®. For further data analysis the Matlab® (The Mathworks Inc.) computing environment was used. MicroRNAs were considered to be significantly differentially expressed (DE) if they obtained a p-value<0.05 and a FDR≤0.05. Two-way average-linkage hierarchical clustering (HCl) with Euclidian distance was performed with Matlab® and Genesis 1.7.2 (Technische Universitaet-Graz, Austria) software. Our MIAME compliant microarray data were deposited in the Gene Expression Omnibus (GEO; www.ncbi.nlm.nih.gov/geo) under accession number GSE48008.

### Quantitative real time polymerase chain reaction (qRT-PCR) validation in FFPE samples and an independent cohort of freshly frozen tissue samples

qRT-PCR for the top DE miRNAs was performed for all FFPE samples for which total RNA was initially extracted (24 ccRCCs, 5 papRCCs, 3 chRCCs, 5 UT-UCs and 40 normal kidney tissue samples) including those used for the microarray analysis.

Additionally, a blinded independent validation set of 40 freshly frozen kidney tissue samples (10 ccRCCs, 3 papRCCs, 4 chRCCs, 3 UT-UCs and 20 normal kidney cortex samples) was used to study the assay performance. The validation set included samples that met the same selection criteria with the initial FFPE cohort, such as >70% tumor cells in each sample and concordance in diagnosis between two independent pathologists. Samples were collected at the Asklipieio General Hospital, Athens. Only samples for which both pathologists agreed in the diagnosis were included in the validation cohort.

Total RNA was extracted by homogenizing approximately 0.2 g of frozen tissue with a tissue homogenizer, followed by isolation using the mirVANA miRNA Isolation Kit (Ambion, Austin, TX) and the concentration was measured spectrophotometrically (Nanodrop technologies, Montchanin, DE). The microRNA in the total RNA sample (80 ng/ul) was converted to cDNA by reverse transcription using the miRCURY LNA Universal RT cDNA synthesis kit (Exiqon, Vedbaek, Denmark). qRT-PCR using appropriate endogenous controls (SNORA66, U6snRNA, RNU1A1) was performed in order to validate the microarray results of the top up-regulated miRNAs: miR-3648, miR-489, miR-638, miR-3656, miR-3687, miR-663b, miR-3126-5p, miR-25-5p, miR-1908, miR-204-3p, miR-711, miR-516b-5p, miR-498 and miR-612; as well as the top down-regulated ones: miR-145-3p, miR-551b-5p, miR-155-3p, miR-106b-3p, miR-140-5p, miR-4284, miR-520g, miR-520e, miR-944, miR-656 and miR-141-3p. The cDNA template was diluted 80x and amplified using SYBR® Green master mix (Exiqon, Vedbaek, Denmark) and LNA microRNA-specific primers on a ViiA 7 Real-Time PCR System (Applied Biosystems). Polymerase activation/DNA denaturation was at 95°C for 10 mins and qPCR amplification was performed for 45 cycles at 95°C for 10 sec and 60°C for 1 min with a ramp-rate 1.6°C/sec, followed by melt curve analysis. All reactions were performed in triplicates. Before proceeding with data normalization, a technical quality assessment was performed based on results of the melting curve, serial dilution curve and no-template-controls. The stability and ranking of the endogenous controls was calculated with the NormFinder algorithim (www.mdl.dk/publicationsnormfinder.htm). For every endogenous control gene, the pair-wise variation with all other endogenous controls was determined as a gene stability measurement M. Genes with expression stability M>1.5 were considered unstable across the samples and unsuitable for endogenous controls in this experiment. Relative expression was performed using the ΔΔCt method as previously described [21], [27]. Data were standardized by log_2_ transformation.

### Locked nucleic acids-in situ hybridization (LNA-ISH)

For LNA-ISH we used the probe sequences: miR-25-5p, 5′-DIG/CAATTGCCCAAGTCTCCGCCT/DIG-3′; miR-3687, 5′-DIG/ACGTCGCACGAACGCCTGT/DIG-3′; LNA U6 snRNA control probe, 5′-DIG/CACGAATTTGCGTGTCATCCTT-3′; LNA scrambled microRNA control probe, 5′-DIG/GTGTAACACGTCTATACGCCCA/DIG-3′. Briefly, 6-µm sections of FFPE tumour and control tissue samples were prepared. Paraffin was melted at 60°C on the day before conducting the ISH experiment and slides were stored overnight at 4°C in an RNase-free environment. The following day, slides were deparaffinized in xylene and ethanol washes, treated with proteinase K (15 μg/ml) for 30 minutes at 37°C, washed in PBS and dehydrated in ethanol. Hybridization mix (1 nM LNA U6 snRNA, 50 nM double-DIG LNA miR-25-5p and miR-3687 probes or 50 nM double-DIG LNA scrambled microRNA probe) (Exiqon, Vedbaek, Denmark) was applied for 2 hours at ∼30°C below the RNA Tm of the corresponding LNA probe, in a StatSpin ThermoBrite System (Abbott Laboratories Ltd). Three different reactions were performed per slide. This was achieved by cutting with scissors the polypropylene coverslips to a size slightly larger than the tissue section. Three serial sections were placed per glass slide, in order to perform the test, the negative and positive controls on the same glass slide. This not only allowed for more efficient work, but permitted the U6 snRNA and scrambled sequence controls to be done under the same exact conditions as miR-25-5p or miR-3687. Slides were washed in SSC buffers prior to incubation with blocking solution. Subsequently, sheep anti-DIG-AP reagent was applied for 1 hour, slides were washed in PBS-T, incubated firstly with AP substrate (NBT/BCIP containing levamisol) for 2 hours at 30°C in a humidifying chamber protected from light, and then in KTBT buffer to stop the reaction. Nuclei were counter stained with Nuclear Fast Red. Slides were carefully rinsed in tap water and dehydrated in ethanol solutions. Finally, slides were mounted, left overnight to allow settlement of the precipitate and analyzed under a light microscope (Nikon eclipse TE2000-U) the subsequent day.

### Computational analysis of the deregulated microRNAs

#### Chromosomal location and phylogenetic analysis of DE miRNAs

Using miRBase v19 (www.mirbase.org/), the chromosomal distribution of the DE miRNAs was compared with reported genomic alterations in the different RCC subtypes and UT-UC. The unaligned fasta format of the mature sequences of miRNA gene families was fetched using miRBase v19 and multiple sequence alignment was subsequently performed with the ClustalW algorithm using T-Coffee (www.ebi.ac.uk/Tools/msa/tcoffee/). Finally, a phylogenetic tree for the miRNA sequences was built using bayesian reconstruction.

#### miRNA target prediction

The DE microRNAs were imported into the miRWalk algorithm (www.ma.uni-heidelberg.de/apps/zmf/mirwalk) and prediction of miRNA target sequences on the 3′UTR of their target genes was performed using 7 nucleotides as the minimum seed number. A multiple comparison using 4 additional algorithms was performed for filtering purposes, each one working based on different sets of properties regarding mRNA-miRNA targeting; TargetScan, miRanda, miRDB and Diana-microT. Filtering was performed for pairs of miRNA-mRNA targets that were predicted by all five algorithms and gave a p-value<0.05, according to the miRWalk algorithm. This p-value depicts the probability of a miRNA 5′-end sequence to be randomly paired with a given 3′UTR mRNA sequence. The validated gene targets of the DE miRNAs in each RCC subtype and UT-UC were also investigated.

#### Ingenuity pathway analysis for the significantly deregulated miRNAs

Ingenuity Pathway Analysis, version 7 (IPA; Ingenuity Systems, USA; www.analysis.ingenuity.com) was performed to identify the molecular pathways and functional groupings based on published literature for the significantly DE miRNAs. miRNAs were uploaded into IPA and overlaid onto a global molecular network developed from information contained in the application. Networks of these genes were generated by IPA based on their connectivity, each ranked by a score. This score is based on the hypergeometric distribution, calculated with the right-tailed Fisher’s Exact Test, and corresponds to the negative log of this p-value. Functional analysis in IPA identified the published biological functions that were most significantly associated with the genes in the network. Genes or gene products are represented as nodes, where shape indicates functional groups, and the biological relationship between two nodes is represented as an edge (line). All lines are supported by at least one reference in literature, textbook, or from canonical information stored in the Ingenuity Pathways knowledge database.

### Functional assays

#### Identification of anti-correlated miRNA/mRNA pairs

Using miRWalk, we searched for gene targets of the top deregulated miRNAs as described above. We also selected the top co-deregulated (top co-up- and top co-down-regulated) mRNA transcripts from 5 ccRCC GEO datasets (accession numbers: GSE6344, GSE4125, GSE15641, GSE781 and GSE6280). For functional analysis we chose the miRNA/mRNA pairs that exhibited anti-correlated profiles between our miRNA profiling and the co-deregulated mRNA transcripts from the aforementioned 5 GEO datasets.

#### Bacterial transformation of ligated products and DNA sequencing

Primers were designed to introduce a SpeI and a HindIII restriction enzyme sites to be cloned into the pMiR-REPORT luciferase vector (**Table S1**). DH5a E. coli cells (Takara, Japan) were gown from glycerol stocks and plasmid DNA was isolated and linearized using SpeI and a HindIII restriction enzymes. PCR-generated inserts were gel purified and ligated into the pMiR-REPORT luciferase vector. Ligation products were transformed into competent DH5a cells and the next day clones were picked and plasmid DNA was isolated. Insert verification included a restriction reaction with SpeI and HindIII. Also, 100 ng of plasmid DNA containing the insert was sequenced to confirm the absence of unwanted mutations, using the forward sequencing primer: 5'-AGGCGATTAAGTTGGGTA-3′.

DNA sequencing of predicted target regions was performed using BigDye V3.1 chemistry on an ABI Prism Genetic Analyzer (Applied Biosystems, CA). Sequencing primers were designed to flank the target region but also included an additional 300 bp on average on each side. Sequence electropherograms were obtained from the ABI Sequencing Analysis V5.2 software (Applied Biosystems, CA) and sequences were imported into MEGA4 software to be aligned against a reference sequence with ClustalW algorithm.

### Real-Time PCR for endogenous miRNA detection

To examine the expression levels of the endogenous miR-23c, mR-204-3p, miR-520a-5p, miR-489, miR-874 and miR-513a-3p in undifferentiated AB8/13 cells, total RNA enriched in small RNAs was isolated from the cells using TRIzol® reagent (Ambion). miRNA specific reverse transcription was performed with the miRCURY LNA Universal RT cDNA synthesis kit (Exiqon, Vedbaek, Denmark). The cDNA template was diluted 80x and amplified using SYBR® Green master mix (Exiqon, Vedbaek, Denmark) and LNA microRNA-specific primers on a ViiA 7 Real-Time PCR System (Applied Biosystems). All reactions were performed in triplicates. SNORA66, U6 snRNA and RNU1A1 were used as reference.

### Transfection of AB8/13 podocytes and luciferase assays

To evaluate the binding efficiency of the top deregulated miRNAs onto their predicted targets, the pMiR-REPORT miRNA Expression Reporter Vector System (AMBION, Texas) was used. The AB8/13 undifferentiated podocyte cells were incubated at 33°C at 5% CO_2_ and cultured in RPMI medium, supplemented with 10% Fetal Bovine Serum (FBS) (Invitrogen, CA), 1% of 100 units/ml Penicillin/Streptomycin (Invitrogen, CA) and 1% Insulin-Transferrin-Selenium (Invitrogen, CA). For the luciferase reporter system experiments, AB8/13 cells were transfected with equal amounts of the pMIR-REPORT luciferase and β-gal vectors (100 ng each), as well as with 25 nM of miRNA inhibitors, mimics, or the AllStars Negative Control scrambled sequence LNA (Qiagen, West Sussex, UK), using lipofectamine 2000 (Invitrogen, CA). The β-gal vector was used for normalization. Every experiment was performed in quadruplicates in 12-well cell culture plates with the appropriate controls. Cells were harvested 16 hours after transfection. The Dual-Light Assay Kit (Applied Biosystems, CA) was used for the quantification of both luciferase and β-gal in an automated luminometer (Sirius, Berthold Detection Systems, Pforzheim, Germany).

### Western blotting

AB8/13 cells were lysed in equal volumes of pre-heated 2xSDS loading buffer (Sodium Dodecyl Sulphate–125 mM Tris-HCl pH 6.8, 20% Glycerol, 2% SDS, 2% β-mercaptoethanol and bromophenol blue) and homogenized using a 2 ml syringe. Whole cell lysates were subsequently electrophoresed in a 12% SDS-Polyacrylamide gel. Gel transfer was held in a wet transfer system on Hybond Polyvinylidene Fluoride (PVDF–Millipore, MA) membranes. Membranes were blocked with 5% non-fat dry milk in PBS/0.01% Tween20 for 1 hour at room temperature. Primary antibody was diluted in milk and added to the membrane for one hour. TFCP2L1 protein was detected with the goat primary polyclonal antibody C-20 (SantaCruz Biotechnology, CA) at around 48 kDa. β-Tubulin was used as loading control by using the T-4026 primary antibody (Sigma, Taufkirchen, Germany). As secondary antibody we used the rabbit anti-mouse antibody (SantaCruz Biotechnology, CA), conjugated with Horseradish Peroxidase (HRP). Proteins were detected using the Enhanced ChemiLuminescence (ECL) Plus Blotting Detection system (Amersham Biosciences, Buckinghamshire, UK) and were visualized on a ChemiDoc MP imaging system using the Image Lab software (Bio-Rad Laboratories).

### Statistical analyses

Microarray data analysis was performed using Matlab®. The two tailed student t-test was used to test the mean differences between two groups. Continuous variables are expressed as median ± standard deviation unless indicated differently. Receiver operating characteristic (ROC) curves were established to evaluate the diagnostic value of deregulated miRNAs for differentiating between RCC subtypes and controls. Pearson's correlation coefficient (CC) was used to identify positive or negative linear correlation between variables. Linear regression analysis using miRNA expression levels as dependent variables and patient age, sex, tumour stage, grade, size and nodal status as independent variables, was also performed to identify miRNAs associated with the aforementioned characteristics. Linear regression analysis along with ANOVA were also performed to develop models that discriminate RCC/UT-UC from the normal kidney samples. The median expression values of all the DE miRNAs were used to perform linear regression analysis. Similar analysis was performed based on tumor grade. All tests were performed 2-sided and a p≤0.05 was considered as statistically significant. Luciferase expression levels were analyzed using one-way ANOVA followed by Tukey post-testing, after being normalized against β-gal expression levels. One-way ANOVA was also used to test densitometry results from western blot analysis, followed by Tukey post-testing. All statistical analyses were performed using GraphPad Prism v.5 and Matlab®.

## Results

### microRNA profiling

**Figure 2:**
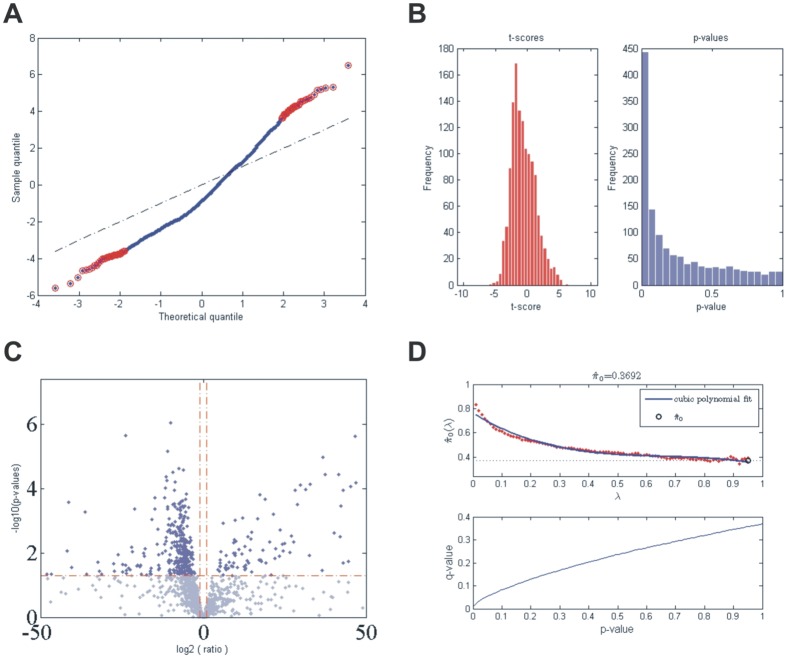
microRNA profiling. Four-hundred and thirty-four miRNAs were statistically significantly deregulated in all RCC subtypes and UT-UC versus the normal kidney. (A) Q-Q (quantile-quantile) plot. Red circles indicate the significantly deregulated miRNAs. (B) Frequencies of the t-scores and p-values. The deregulated miRNAs had a p<0.05. (C) The volcano-plot depicts the 434 statistically significantly deregulated miRNAs in ccRCC, papRCC, chRCC and UT-UC versus the normal kidney, of which the majority was significantly down-regulated in the cancerous tissue compared to the latter. (D) FDR diagram depicting the percentage of FDR with respect to p-value along with a plot of the estimated a priori probability that the null hypothesis π(0), is true versus the tuning parameter, lambda, λ, with a cubic polynomial fitting curve.

**Figure 3:**
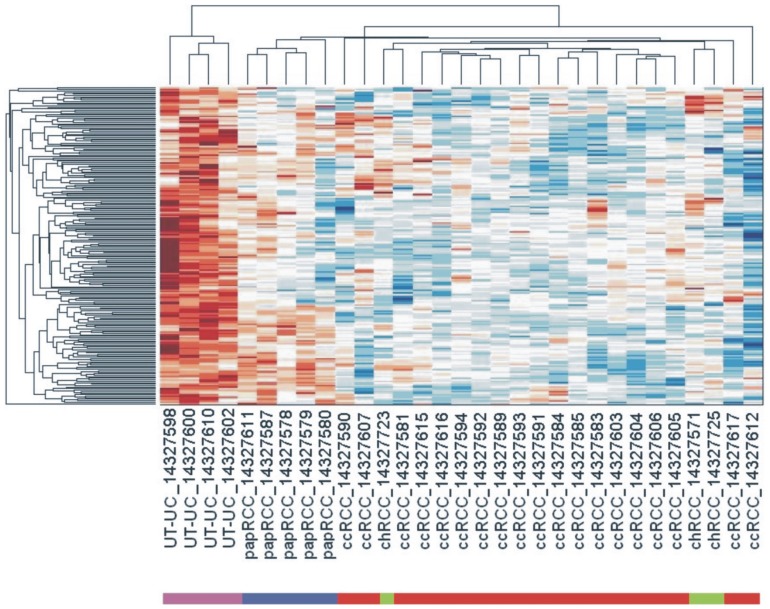
Hierarchical Clustering (HCl). The unsupervised two-way HCl with Euclidian distance depicts differential miRNA expression in ccRCC, papRCC, chRCC and UT-UC. The log_2_ fold change in each RCC subtype and UT-UC versus the normal kidney tissue was used to construct the heat map. miRNA profiling accurately discriminated between RCC and UT-UC, as well as among ccRCC, papRCC and chRCC. ccRCC, clear cell renal cell carcinoma; papRCC, papillary renal cell carcinoma; chRCC, chromophobe renal cell carcinoma; UT-UC, upper tract urothelial carcinoma. Red and blue colours show significant up- or down-regulation of each miRNA in the tumour versus the normal kidney, respectively.

**Figure 4:**
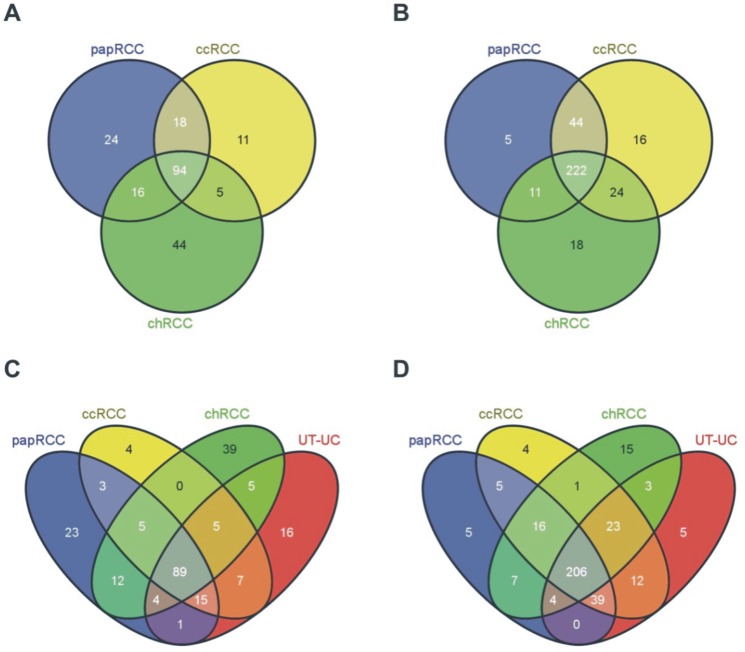
Overlapping relationship of the deregulated miRNAs. Venn diagrams illustrate the overlapping relationship of the number of up-regulated miRNAs among RCC subtypes (A), down-regulated miRNAs among RCC subtypes (B), up-regulated miRNAs among RCC subtypes and UT-UCs (C), down-regulated miRNAs between RCC subtypes and UT-UCs (D). Ninety-four miRNAs were co-upregulated among ccRCC, papRCC and chRCC; and 11, 44 and 24 miRNAs were specifically up-regulated in each one of the three RCC subtypes (ccRCC, chRCC and papRCC), respectively. On the other hand, 222 miRNAs were co-down-regulated in the three RCC subtypes, whereas 16, 18 and 5 miRNAs were specifically down-regulated in ccRCC, chRCC and papRCC, respectively. When the DE miRNAs in each RCC subtype were combined with those in UT-UC, we identified 89 and 206 miRNAs that were up- and down-regulated, respectively in all tumor types.

microRNA profiling was performed retrospectively in a total of 27 RCCs (3 chRCC, 5 papRCC and 19 ccRCC), 4 UT-UCs and 20 normal kidney tissues. We identified 434 deregulated (DE) miRNAs (p<0.05 and FDR<0.05) in all RCC subtypes and UT-UC versus the normal kidney. The majority of them was down-regulated in RCCs but over-expressed in UT-UCs. Overall, 126 miRNAs (29%) showed increased expression and 303 miRNAs (69.8%) had decreased expression in RCCs and UT-UCs vs. the normal kidneys (**Figure 2**). Specifically, 374, 420, 421 and 409 miRNAs were 90% consistently down-regulated in ccRCC, papRCC, chRCC and UT-UC, respectively. Unsupervised two-way hierarchical clustering (HCl) with Euclidian distance accurately discriminated between RCC and UT-UC. Apart from one chRCC sample that was clustered with ccRCCs, HCl also managed to successfully classify the 3 RCC subtypes among them. Furthermore, it showed that ccRCC is more closely related to papRCC and that both are distinct from chRCC or UT-UC (**Figure 3**). HCl pair-tissue-wise also discriminated perfectly ccRCC from papRCC, chRCC from ccRCC, chRCC from papRCC, as well as ccRCC from UT-UC, papRCC from UT-UC and chRCC from UT-UC (**Figure S1**). **Table S2** lists the top deregulated miRNAs for each RCC subtype and UT-UC. The overlapping relationship of the deregulated miRNAs among the three RCC subtypes, as well as among all tumour types is depicted in **Figure 4** **.** Tissue-specific miRNAs, including those that can distinguish RCCs from UT-UC, were identified. We found 16 miRNAs that were specifically down-regulated (miR-3144-5p, miR-193b-3p, miR-587, miR-3117-3p, miR-769-5p, miR-617, miR-3615, miR-148b-5p, miR-24-2-5p, miR-142-5p, miR-3164, miR-514b-5p, miR-375, miR-520c-3p, miR-191-5p and miR-520b/miR-520c-3p) and 5 miRNAs that were specifically up-regulated in UT-UC (miR-885-5p, miR-1912, miR-378a-5p, miR-24-3p and miR-26a-2-3p) **Table S3**.

**Figure 5:**
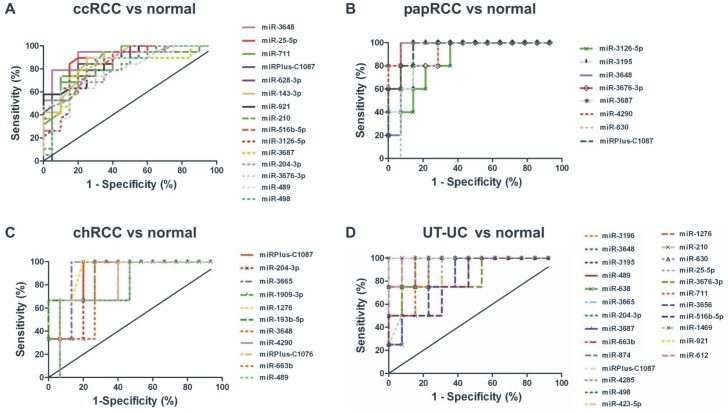
ROC analysis using microarray data. ROC curves of the top 30 up-regulated miRNAs in each tissue type using the microarray expression data. Of them, the miRNAs with a p<0.01 and an AUC>0.8 were selected as successful distinguishing markers between cancerous and normal tissues. The median area under the curve (AUC) for ccRCC was 0.85 (A); for papRCC, median AUC = 0.94 (B); for chRCC, median AUC = 0.84 (C) and for UT-UC, median AUC = 0.94 (D).

ROC analysis of the top 30 up-regulated miRNAs in each tissue type, using the microarray expression data, evaluated the extent to which they could separate each tumour entity from the normal kidney tissue. Of them, the miRNAs with a p<0.01 and an AUC>0.84 were selected as successful distinguishing markers between each tumor subtype and the normal kidney tissue (**Figure 5**). The ROC statistics of the miRNAs that exhibited the best discriminatory ability are depicted in **Table 2**.

**Table 2:** ROC statistics of the miRNAs that exhibited the best discriminatory ability in ccRCC (A), papRCC (B), chRCC (C) and UT-UC (D) vs. the normal kidney tissue.

**A. miRNAs in ccRCC**	**AUC**	**S.E.**	**95% C.I.**	**p value**	**Sensitivity**		**1-Specificity**		
miR-3648	0.918	0.057	0.746–0.971	0.0001	100.0		90.0		
miR-143-3p	0.878	0.060	0.735–0.969	0.0001	100.0		95.0		
miR-25-5p	0.878	0.066	0.723–0.982	0.0001	100.0		95.0		
miR-628-3p	0.868	0.064	0.724–0.975	0.0001	100.0		90.0		
miR-921	0.859	0.060	0.727–0.964	0.0002	100.0		95.0		
miR-210	0.853	0.066	0.699–0.958	0.0004	100.0		95.0		
miR-711	0.853	0.071	0.678–0.958	0.0006	100.0		95.0		
miRPlus-C1087	0.850	0.067	0.684–0.947	0.0007	100.0		94.7		
miR-516b-5p	0.846	0.069	0.674–0.944	0.0009	100.0		95.0		
miR-3126-5p	0.828	0.072	0.660–0.944	0.0012	100.0		90.0		
miR-3687	0.818	0.072	0.661–0.941	0.0013	100.0		95.0		
miR-204-3p	0.816	0.075	0.650–0.946	0.0014	100.0		90.0		
miR-3676-3p	0.809	0.074	0.650–0.941	0.0015	100.0		90.0		
miR-489	0.802	0.075	0.644–0.939	0.0018	100.0		95.0		
miR-498	0.801	0.072	0.645–0.928	0.0022	100.0		90.0		
**B. miRNAs in papRCC**	**AUC**	**S.E.**	**95% C.I.**	**p value**	**Sensitivity**		**1-Specificity**		
miR-4290	0.985	0.035	0.901–1.042	0.002	100		92.8		
miR-3687	0.971	0.057	0.829–1.056	0.004	100		92.8		
miRPlus-C1087	0.957	0.052	0.840–1.046	0.004	100		92.8		
miR-3648	0.942	0.063	0.803–1.054	0.005	100		92.8		
miR-3676-3p	0.928	0.043	0.871–1.043	0.003	100		92.8		
miR-3195	0.928	0.022	0.941–1.030	0.001	100		92.8		
miR-630	0.871	0.035	0.901–1.042	0.002	100		92.8		
miR-3126-5p	0.857	0.059	0.812–1.045	0.005	100		92.8		
**C. miRNAs in chRCC**	**AUC**	**S.E.**	**95% C.I.**	**p value**	**Sensitivity**		**1-Specificity**		
miR-1276	0.944	0.122	0.516–0.994	0.1731	100		93.33		
miRPlus-C1087	0.911	0.133	0.538–1.061	0.109	100		93.33		
miR-3665	0.911	0.138	0.573–1.116	0.066	100		93.33		
miR-1909-3p	0.889	0.133	0.538–1.061	0.109	100		93.33		
miR-204-3p	0.867	0.138	0.485–1.026	0.173	100		93.33		
miR-193b-5p	0.844	0.179	0.381–1.085	0.213	100		93.33		
miR-3648	0.822	0.169	0.356–1.021	0.314	100		93.33		
miR-4290	0.822	0.163	0.414–1.052	0.213	100		93.33		
miRPlus-C1076	0.822	0.074	0.764–1.057	0.028	100		93.33		
miR-663b	0.800	0.198	0.255–1.034	0.441	100		93.33		
miR-489	0.800	0.194	0.329–1.092	0.26	100		93.33		
**D. miRNAs in UT-UC**	**AUC**	**S.E.**	**95% C.I.**	**p value**		**Sensitivity**		**1-Specificity**	
miR-3648	1.00	0.063	0.818–1.066	0.009		100		92.308	
miR-489	1.00	0.078	0.768–1.078	0.001		100		92.308	
miR-3665	1.00	0	1.000–1.000	0.003		100		92.308	
miR-874	1.00	0.063	0.818–1.066	0.009		100		92.308	
miR-4285	1.00	0	1.000–1.000	0.003		100		92.308	
miR-210	1.00	0	1.000–1.000	0.003		100		92.308	
miR-612	0.98	0.068	0.788–1.058	0.012		100		92.308	
miR-663b	0.96	0.046	0.869–1.054	0.006		100		92.308	
miR-3687	0.96	0.045	0.872–1.051	0.006		100		92.308	
miR-1276	0.96	0	1.000–1.000	0.003		100		92.308	
miR-1908	0.94	0.056	0.831–1.053	0.009		100		92.308	
miR-3195	0.94	0.087	0.713–1.055	0.023		100		92.308	
miRPlus-C1087	0.94	0	1.000–1.000	0.003		100		92.308	
miR-498	0.94	0.063	0.818–1.066	0.009		100		92.308	
miR-1469	0.94	0.091	0.686–1.044	0.031		100		92.308	
miR-630	0.94	0.094	0.718–1.089	0.017		100		92.308	
miR-3196	0.92	0.124	0.602–1.090	0.041		100		92.308	
miR-204-3p	0.92	0.045	0.872–1.051	0.006		100		92.308	
miR-185-3p	0.91	0.06	0.824–1.060	0.009		100		92.308	
miR-423-5p	0.90	0.122	0.567–1.048	0.07		100		92.308	
miR-921	0.88	0.087	0.712–1.057	0.023		100		92.308	
miR-638	0.86	0	1.000–1.000	0.003		100		92.308	
miR-25-5p	0.86	0.029	0.922–1.039	0.004		100		92.308	
miR-3676-3p	0.84	0.071	0.774–1.053	0.014		100		92.308	
miR-711	0.84	0.046	0.869–1.054	0.006		100		92.308	
miR-3656	0.82	0	1.000–1.000	0.003		100		92.308	
miR-516b-5p	0.80	0.063	0.818–1.066	0.009		100		92.308	

Due to the small chRCC sample number (n = 3), no statistical significance could be gained. AUC, area under the curve; S.E., standard error; 95% C.I., 95% confidence interval.

### Validation of deregulated miRNA expression by qRT-PCR

**Figure 6:**
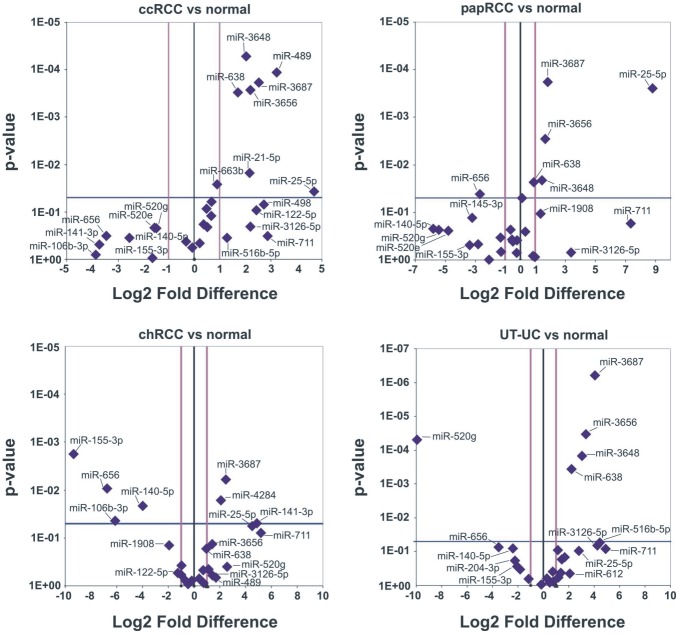
qRT-PCR validation of the top 25 deregulated miRNAs. The Volcano-plots depict the significantly deregulated miRNAs in ccRCC, papRCC, chRCC and UT-UC. Eight miRNAs were significantly up-regulated in ccRCC, 5 in papRCC, 3 in chRCC and 4 in UT-UC. On the other hand, miR-656 was significantly down-regulated in papRCC; miR-155-3p, miR-106b-3p, miR-140-5p and miR-656 were significantly down-regulated in chRCC; and miR-520g was significantly down-regulated in UT-UC. The threshold of statistically significant difference was set at p<0.05 and log_2_ fold change>2.

We verified by qRT-PCR the expression levels of the top 25 deregulated miRNAs as acquired by miRNA profiling (14 most up-regulated and 11 most down-regulated miRNAs). These included: miR-3648, miR-489, miR-638, miR-3656, miR-3687, miR-663b, miR-3126-5p, miR-25-5p, miR-1908, miR-204-3p, miR-711, miR-516b-5p, miR-498 and miR-612 (top up-regulated miRNAs); as well as miR-145-3p, miR-551b-5p, miR-155-3p, miR-106b-3p, miR-140-5p, miR-4284, miR-520g, miR-520e, miR-944, miR-656 and miR-141-3p (top down-regulated miRNAs). The validated significantly deregulated miRNAs in ccRCC, papRCC, chRCC and UT-UC are depicted in **Figure 6**.

The frequencies of differential expression were mostly consistent across the same subtypes. In keeping with the microarray results, some miRNAs showed a similar expression pattern between the subtypes. For example, miR-3687 exhibited a similar up-regulation pattern in all RCC subtypes and in UT-UC; miR-3656, miR-638 and miR-3648 were similarly up-regulated in ccRCC, papRCC and UT-UC and miR-25-5p was similarly up-regulated in ccRCC and papRCC. Also, miR-656 was equally down-regulated in papRCC and chRCC.

**Figure 7:**
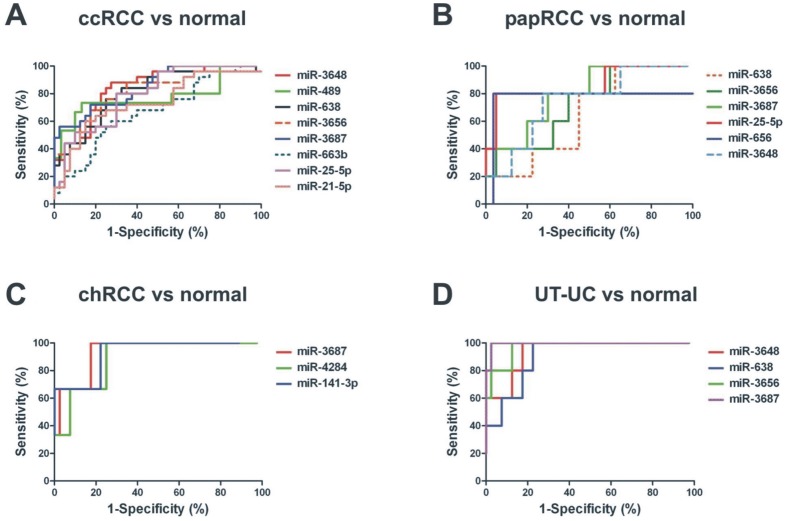
ROC analysis using qRT-PCR data. ROC curves of the significantly deregulated miRNAs, using the expression data from qRT-PCR analysis. Of them, the miRNAs with a p<0.01 and an AUC>0.8 were selected as successful distinguishing markers between cancerous and normal kidney tissues. The median area under the curve (AUC) for ccRCC was 0.802 (A); for papRCC, median AUC = 0.756 (B); for chRCC, median AUC = 0.926 (C) and for UT-UC, median AUC = 0.955 (D).

**Figure 8:**
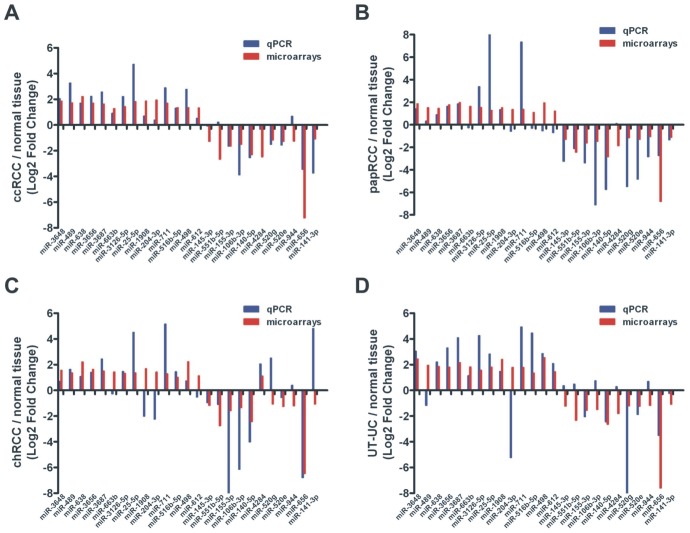
Correlation between microarrays and qRT-PCR. Median log_2_ fold change expression levels of the 14 most up-regulated and 11 most down-regulated miRNAs between ccRCC, papRCC, chRCC and UT-UC and the normal kidney tissue, as determined by both qRT-PCR and microarray analysis. As shown in the figure, qRT-PCR and microarray results were highly compatible. The most identical results between the two techniques were those for ccRCC, which was expected due to the high sample number (Pearson’s CC = 0.778, p<0.001). The qRT-PCR results for papRCC, chRCC and UT-UC also revealed similar deregulation patterns with those of the microarray experimentation, however the correlation coefficients were lower, apparently due to small sample number (in papRCC, CC = 0.596, p = 0.002; in chRCC CC = 0.570, p = 0.003; in UT-UC, CC = 0.517, p = 0.009).

In order to further validate the capability of the DE miRNAs to discriminate between tumour and normal kidney tissue, ROC curves were further constructed for the deregulated miRNAs in each RCC subtype and in UT-UC, using the normalized qRT-PCR data. miRNAs with a p<0.01 and an AUC>0.8 were then selected as successful distinguishing markers between cancerous and normal kidney tissue (**Figure 7**). miR-3648, miR-638, miR-3656 and miR-3687 were excellent discriminators between ccRCC and normal tissue (median AUC>0.8, p<0.0001); miR-3687 and miR-25-5p were very good discriminators between papRCC and normal tissue (median AUC>0.8, p<0.05); miR-3687, miR-4284 and miR-141-3p were very good discriminators between chRCC and normal tissue (median AUC>0.8, p<0.05); and miR-3648, miR-3656 and miR-3687 discriminated perfectly UT-UC from the normal tissue (median AUC>0.94, p<0.001). ROC curve data are shown in **Table 3**. The qRT-PCR results revealed similar expression patterns with the microarray results (**Figure 8**).

**Table 3:** ROC statistics of the miRNAs that exhibited the best discriminatory ability in ccRCC (A), papRCC (B), chRCC (C), and UT-UC (D) vs. the normal kidney tissue, respectively.

**A. miRNAs in ccRCC**	**AUC**	**S.E.**	**95% C.I.**	**p value**	**Sensitivity**	**1-Specificity**
miR-3648	0.839	0.049	0.742–0.935	<0.0001	100.0	97.5
miR-489	0.773	0.086	0.602–0.943	0.003	100.0	96.66
miR-638	0.807	0.056	0.697–0.917	<0.0001	100.0	97.5
miR-3656	0.817	0.055	0.707–0.926	<0.0001	100.0	97.5
miR-3687	0.847	0.048	0.752–0.941	<0.0001	100.0	97.5
miR-25-5p	0.796	0.054	0.689–0.902	<0.0001	100.0	97.5
miR-21-5p	0.737	0.065	0.608–0.866	0.001	100.0	96.0
miR-663b	0.668	0.068	0.533–0.802	0.023	100.0	97.5
**B. miRNAs in papRCC**	**AUC**	**S.E.**	**95% C.I.**	**p value**	**Sensitivity**	**1-Specificity**
miR-3648	0.745	0.108	0.532–0.957	0.076	100	97.5
miR-638	0.650	0.111	0.431–0.868	0.278	100	97.5
miR-3656	0.725	0.110	0.508–0.942	0.104	100	97.5
miR-3687	0.800	0.094	0.614–0.985	0.030	100	97.5
miR-25-5p	0.865	0.102	0.663–1.066	0.008	100	97.5
miR-656	0.770	0.174	0.427–1.113	0.058	100	97.5
**C. miRNAs in chRCC**	**AUC**	**S.E.**	**95% C.I.**	**p value**	**Sensitivity**	**1-Specificity**
miR-3687	0.933	0.053	0.828–1.038	0.013	100	97.5
miR-4284	0.891	0.071	0.752–1.031	0.025	100	97.5
miR-141-3p	0.925	0.084	0.759–1.092	0.033	100	88.89
**D. miRNAs in UT-UC**	**AUC**	**S.E.**	**95% C.I.**	**p value**	**Sensitivity**	**1-Specificity**
miR-3648	0.940	0.041	0.857–1.022	0.001	100	97.5
miR-638	0.905	0.052	0.801–1.008	0.003	100	97.5
miR-3656	0.970	0.027	0.916–1.024	0.0006	100	97.5
miR-3687	0.995	0.007	0.979–1.011	0.0003	100	97.5

AUC, area under the curve; S.E., standard error; 95% C.I., 95% confidence interval.

**Figure 9:**
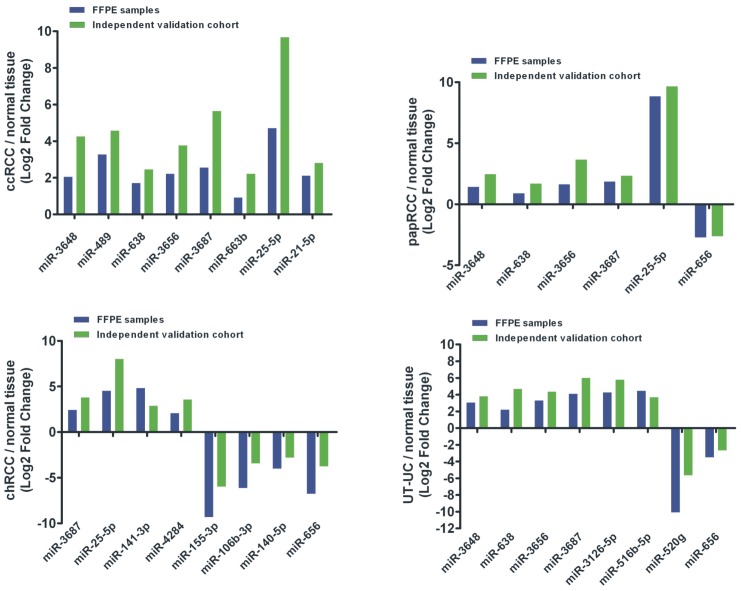
Validation in a blinded independent cohort. A blinded independent validation cohort composed of 40 freshly frozen ccRCC, papRCC, chRCC, UT-UC and normal kidney samples was also used to validate the discriminatory ability of the qRT-PCR verified deregulated miRNAs. The results were highly repeatable between the two cohorts, recapitulating the specificity of these miRNAs in each tumour group.

A blinded independent validation set composed of 40 freshly frozen ccRCC, papRCC, chRCC, UT-UC and normal kidney samples was also used to validate the discriminatory ability of the qRT-PCR verified deregulated miRNAs. The results were highly repeatable between the two cohorts, recapitulating the specificity of these miRNAs in each tumour group (in ccRCC, Pearson's CC = 0.987, p<0.001; in papRCC, Pearson's CC = 0.917, p = 0.001; in chRCC, Pearson's CC = 0.952, p<0.001; in UT-UC, Pearson's CC = 0.926, p = 0.001) (**Figure 9**).

### miRNA expression and clinical characteristics of the patients

Only a few correlations between the expression levels of the DE miRNAs (verified by qRT-PCR) with the tumour stage, grade and size were identified. The expression levels of the significantly deregulated miRNAs that were verified by qRT-PCR, were correlated with the tumour stage, grade and size. Grade 4 ccRCCs exhibited higher miR-3656 and miR-3687 levels vs. those of grade 3 (p = 0.028 and p = 0.0497, respectively; unpaired t-test). Furthermore, grade 4 ccRCCs exhibited higher miR-21-5p levels vs. grade 2 ccRCCs (p = 0.0449, unpaired t-test). We also found a significant association between miR-21-5p expression and ccRCC size (Pearson's CC = 0.442, p = 0.031). The levels of miR-25-5p were significantly associated with papRCC tumour size (Pearson's CC = 0.935, p = 0.019). Grade II UT-UCs expressed higher levels of miR-3648 and miR-638 vs. grade III ones (p = 0.0153 and p = 0.0057, respectively). Finally, miR-638 was correlated with tumour size (Pearson's CC = 0.878, p = 0.050).

Furthermore, miRNA expression was validated using one-way ANOVA along with linear regression. Of note, miR-204-3p, miR-25-5p, miR-3648, miR-3687, miR-489, miR-516b-5p, miR-638 and miR-663b exhibited a linear expression pattern among the 3 RCC subtypes, UT-UC and the normal kidney samples (*R^2^* range = 0.93–0.98). Also, linear regression could classify between RCC and UT-UC, with the latter expressing higher levels of the pre-mentioned miRNAs (**Figure S2**).

Similarly, high linearity was achieved when we grouped together ccRCC, papRCC and chRCC samples, and we compared them to the UT-UC and the normal kidney cases (miR-204-3p, *R^2^>*0.99; miR-25-5p, *R^2^>*0.83; miR-3648, *R^2^>*0.98; miR-3687, *R^2^>*0.95; miR-489, *R^2^>*0.99; and miR-638, *R^2^>*0.98) (**Figure S3**).

Linear regression of RCC grades with respect to miRNA expression, revealed that miR-148b-5p, miR-1910 and miR-200b-5p, miR-210 and miR-3656, presented linear behavior in a descending order of the RCC grade; whereas miR-3687 and miR-654-5p manifested linear behavior in ascending order of the RCC grade (**Figure S4**).

Furthermore, miR-148a-5p and miR-940 manifested excellent linear behavior in an descending order of age (*R^2^* = 0.96 and 0.99, respectively), pointing out that both might be age-specific. Also, regression analysis showed that miR-145-3p and miR-1973 manifested increasing expression levels with ascending tumor size (<5cm, 5-10cm and 10-15 cm) (*R^2^* = 0.95 and 0.92, respectively).

Other varieties, such as a patient’s sex, age or tumor stage, were not associated with any other miRNA expression levels.

### Pair-wise miRNA correlations

Several DE microRNAs were correlated among each other. In ccRCC, the statistically significant Spearman’s correlation coefficients ranged from r_s_ = –0.585 (miR-106b-3p to miR-612; p = 0.002) to r_s_ = 0.843 (miR-638 to miR-3687; p<0.0001). In papRCC, the statistically significant Spearman’s correlation coefficients ranged from r_s_ = –0.90 (miR-145-3p to miR-711; and miR-21-5p to miR-141-3p; both p = 0.083) to r_s_ =  1.0 (miR-638 to miR-3648; miR-663b to miR-3656; miR-516b-5p to miR-489; miR-498 to miR-489; miR-155-3p to miR-489, among others; all p = 0.017). Furthermore, in UT-UC the statistically significant Spearman’s correlation coefficients ranged from r_s_ =  –0.9 (miR-551b-5p to miR-3126-5p; miR-520g to miR-3126-5p; miR-141-3p to miR-1908, among others; all p = 0.083) to r_s_ =  1.0 (miR-489 to miR-3648; miR-3656 to miR-3648; miR-3656 to miR-489; miR-155-3p to miR-638; miR-122-5p to miR-638, among others; all p = 0.017). Finally, in the normal kidney the statistically significant Spearman’s correlation coefficients ranged from r_s_ = –0.325 (miR-551b-5p to miR-155-3p; p = 0.041) to r_s_ = 0.885 (miR-3648 to miR-3656; p<0.0001).

### Validation of the pattern of expression of miR-25-5p and miR-3687 by LNA-ISH

**Figure 10:**
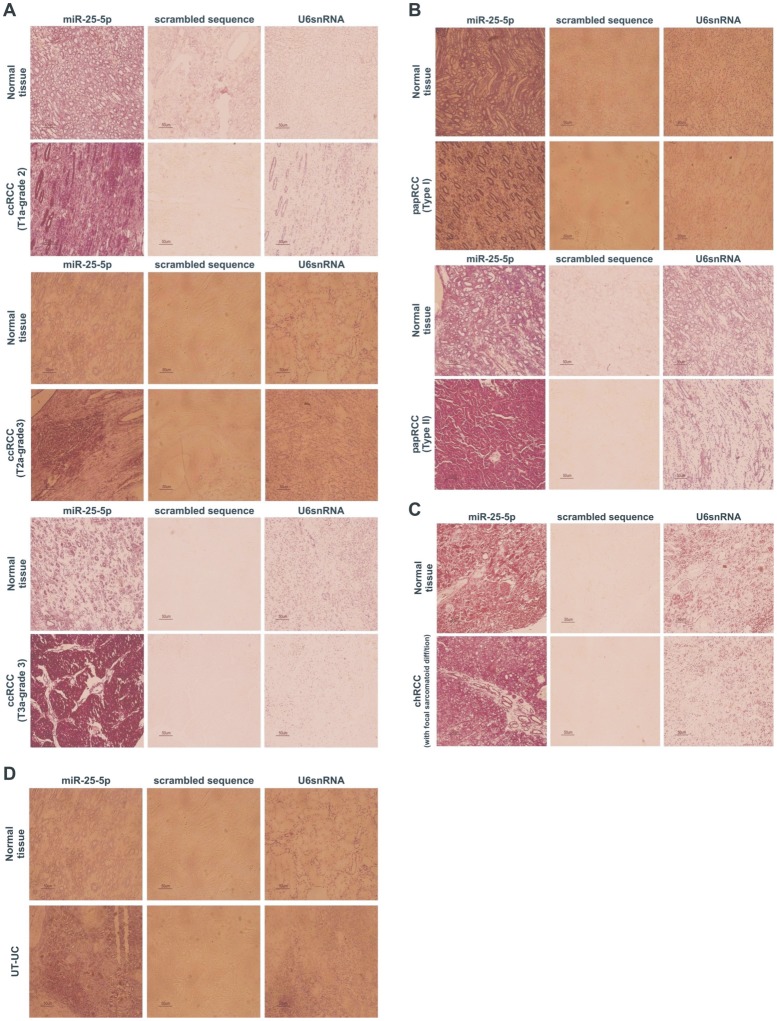
Locked Nucleic Acids-In Situ Hybridization (LNA-ISH). LNA-ISH for miR-25-5p in ccRCC (A), papRCC (B), chRCC (C) and UT-UC (D). miR-25-5p was confined to the cytoplasm both in normal and tumour sections. The nuclear expression of U6 snRNA (positive control) was confirmed in all the patient samples, whereas the scrambled oligonucleotide was negative in all samples. miR-25-5p high expression was confirmed in all ccRCC sections by LNA-ISH. ccRCCs of high stage and grade stained stronger miR-25-5p compared to lower stage and grade ccRCCs. Each ccRCC section was compared against its corresponding normal kidney section (A). papRCCs of type II stained stronger for miR-25-5p compared to type I papRCCs (B). Validating the qRT-PCR results, miR-25-5p did not stain stronger in chRCC sections vs. the normal tissue ones. This was also confirmed for chRCCs with focal sarcomatoid differentiation, suggesting that miR-25-5p does not play any role in the metastatic behavior of the tumour (C). Verifying the qRT-PCR results, miR-25-5p was not significantly stronger in UT-UC vs. the normal tissue sections (D).

In situ hybridization using digoxigenin labeled LNA probes permitted the spatial localization of two of the most deregulated miRNAs: a) miR-25-5p which was significantly up-regulated in ccRCC and papRCC; and b) miR-3687 which was significantly up-regulated in all 3 RCC subtypes as well as in UT-UC. The detection of both miRNAs was confined to the cytoplasm. The nuclear expression of U6 snRNA was confirmed in all patient samples, whereas the scrambled oligonucleotide was negative in all samples. Each RCC or UT-UC section was compared against its corresponding normal kidney section. The elevated expression of miR-25-5p was confirmed in all ccRCC sections. Furthermore, ccRCCs of high stage and grade stained stronger miR-25-5p compared to lower stage and grade tumours. Also, papRCCs of type II stained stronger for miR-25-5p compared to type I papRCCs (**Figure 10**). Likewise, the elevated expression of miR-3687 was confirmed in the majority of both RCC and UT-UC sections.

### Correlation of miRNA deregulation and chromosomal aberrations in RCC and UT-UC

**Figure 11:**
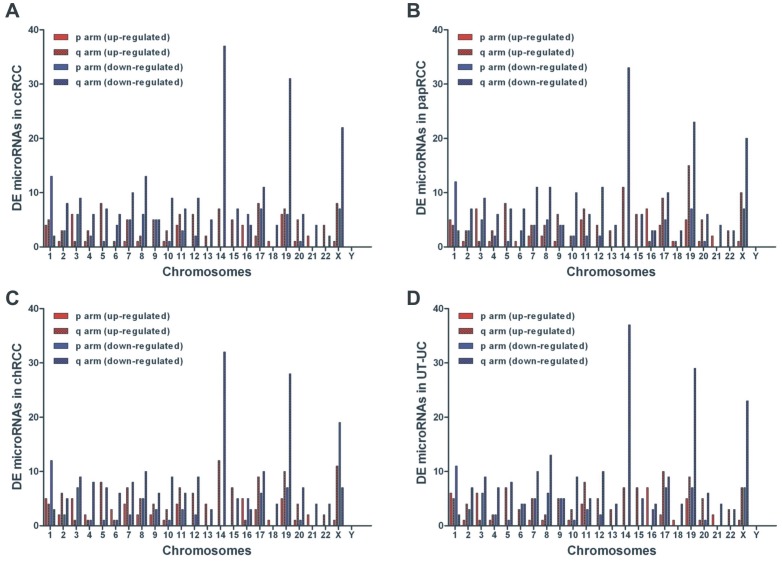
Correlation of miRNA deregulation and chromosomal aberrations. Chromosomal mapping for the deregulated miRNAs in ccRCC (A), papRCC (B), chRCC (C) and UT-UC (D). In ccRCC, the up-regulated miRNAs were mainly mapped on chromosomes Xq, 17q, 5q, 14q, 19q, 11q, 12q, 3p and 10p (in descending order); whereas the down-regulated ones were mainly mapped on chromosomes 14q, 19q, Xq, 8q, 1p, 17q, 7q, 3q, 10q, 12q and 2q (A). In papRCC, the up-regulated miRNAs were mainly mapped on chromosomes 19q, 14q, Xq, 5q, 3p, 16p, 11q, 9q and 15q; whereas the down-regulated miRNAs were mainly mapped on chromosomes 14q, 19q, Xq, 1p, 7q, 8q, 12q, 10q, 17q, 3q, 19p, Xp, 2q, 5, 6q, 4q, 11q, 15q and 20q (B). In chRCC, the up-regulated miRNAs were mainly mapped on chromosomes 14q, Xq, 19q, 17q, 5q, 7q, 11q, 15q, 2q and 12q; whereas the down-regulated ones were mainly mapped on chromosomes 14q, 19q, Xp, 3p, 8q, 17q, 3q, 10q, 12q, 4q, 7q, 3p, 5q, 19p and 20q (C). In UT-UC, the up-regulated miRNAs were mostly mapped on chromosomes 17q, 19q, 11q, 16p, 5q, 14q, 15q, Xq, 1p and 3p; whereas the down-regulated miRNAs were mainly mapped on chromosomes 14q, 19q, Xq, 8q, 1p, 7q, 12q, 3q, 10q, 5q, 17p, 19p, Xp, 2q, 4q, 3p and 8p (D).

The chromosomal locations of the DE miRNAs were compared to the most commonly reported chromosomal aberrations in ccRCC, chRCC, papRCC [28] or UT-UC [29]. In general, chromosomes 14q, 19q, Xq exhibited the highest number of deregulated miRNAs. In each RCC subtype, miRNAs had deregulated patterns that agreed with some of the previously reported chromosomal gains and losses; i.e., up-regulated miRNAs were located in regions of chromosomal gains and vice versa. **Table S4** lists a summary of the reported chromosomal abnormalities associated with the three RCC subtypes and UT-UC, as well as some of the well-studied genes located within those aberrated regions that are involved in the pathogenesis of the tumour subtypes. Many of the significantly deregulated miRNAs are located within cytogenetic regions that are significantly aberrated in the different subtypes. Chromosomal mapping for the deregulated miRNAs in each RCC subtype and in UT-UC is depicted in **Figure 11**.

### Phylogenetic analysis of the DE miRNAs in RCC

Phylogenetic analysis showed that 340 of the 434 (78.3%) DE miRNAs were highly conserved among species. Specifically, 70.68% of the up-regulated miRNAs and 81.22% of the down-regulated miRNAs were phylogenetically conserved among species, respectively. **Figure S5** shows representative multiple sequence alignments and trees of miR-489 and miR-25-5p.

**Figure 12:**
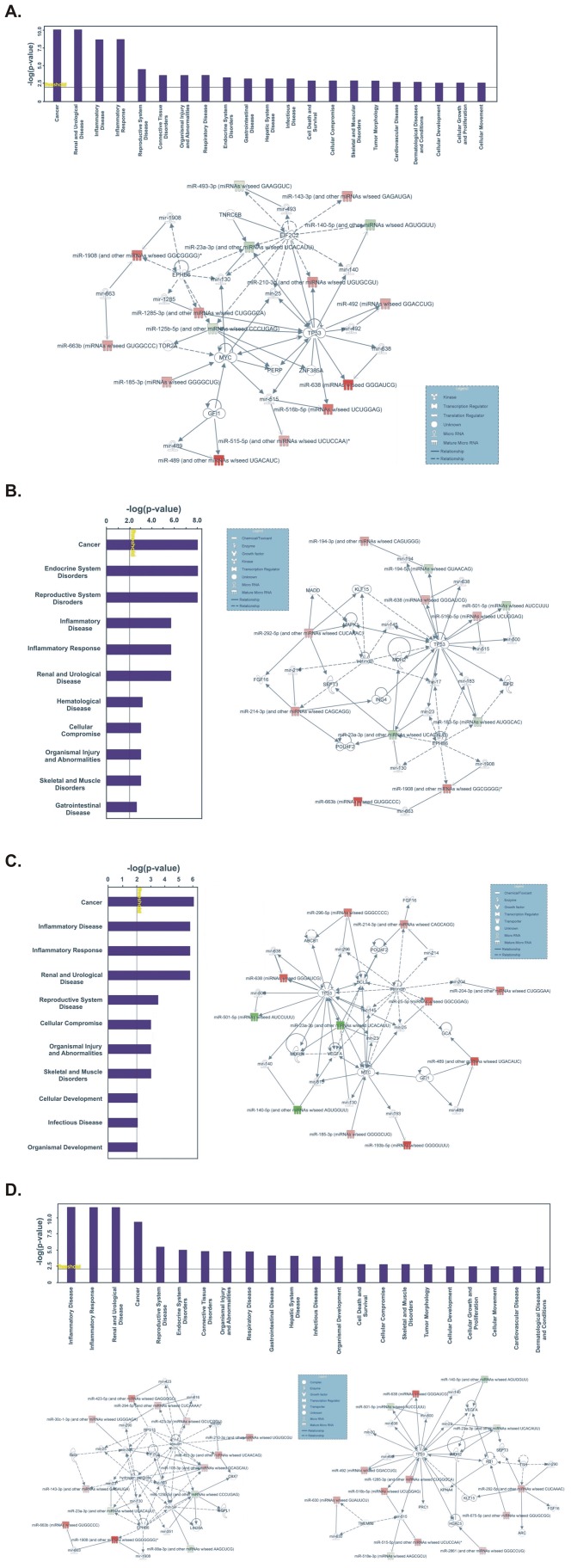
Ingenuity pathway analysis (IPA). A. In ccRCC, the most important biological functions of the top deregulated miRNAs were: 1) Cancer (p = 8.71E-11-4.91E-02); 2) Renal and Urological Disease (p = 8.71E-11-2.12E-09); 3) Inflammatory Disease (p = 2.12E-09-2.14E-03); 4) Inflammatory Response (p = 2.12E-09-2.4E-04) and 5) Reproductive System Disease (p = 3.37E-05-2.58E-02). The associated functions of the major miRNA network (score = 30) were: Inflammatory disease, inflammatory response, renal inflammation. Argonaute RISC catalytic component 2 (EIF2C2), tumour protein p53 (TP53), v-myc myelocytomatosis viral oncogene homolog (MYC) and EPH receptor B6 (EPHB6) constituted some of the major central nodes in this network. B. In papRCC, the most important biological functions of the top deregulated miRNAs were: 1) Cancer (p = 9.4E-09-4.15E-02); 2) Endocrine System Disorders (p = 9.4E-09-4.54E-02); 3) Reproductive System Disease (p = 9.4E-09-5.11E-03); 4) Inflammatory Disease (p = 2.17E-06-2.17E-06) and 5) Inflammatory Response (p = 2.17E-06-1.65E-03). The associated functions of the major miRNA network (score = 22) were: Endocrine System Disorders, Reproductive System Disease, Cellular Development. Tumour protein p53 (TP53) and EPH receptor B6 (EPHB6) constituted some of the major central nodes in this network. C. In chRCC, the most important biological functions of the top deregulated miRNAs were: 1) Cancer (p = 8.99E-07-4.73E-02); 2) Inflammatory Disease (p = 1.64E-06-1.64E-06); 3) Inflammatory Response (p = 1.64E-06-1.19E-03); 4) Renal and Urological Disease (p = 1.64E-06-7.96E-03) and 5) Reproductive System Disease (p = 3.02E-04-2.39E-02). The associated functions of the major miRNA network (score = 22) were: Hereditary Disorder, Skeletal and Muscular Disorders, Developmental Disorder. Tumour protein p53 (TP53), B-cell CLL/lymphoma 2 (BCL2) vascular endothelial growth factor A (VEGFA) and v-myc myelocytomatosis viral oncogene homolog (MYC) constituted some of the major central nodes in this network. D. In UT-UC, the most important biological functions of the top deregulated miRNAs were: 1) Inflammatory Disease (p = 2.7E-12-3.42E-02); 2) Inflammatory Response (p = 2.7E-12-1.75E-05); 3) Renal and Urological Disease (p = 2.7E-12-3.35E-09); 4) Cancer (p = 4.67E-10-4.53E-02) and 5) Reproductive System Disease (p = 3.82E-06-4.96E-02). The associated functions of the 2 major miRNA networks were: 1) Connective Tissue Disorders, Inflammatory Disease, Inflammatory Response (score = 25); and 2) Cancer, Reproductive System Disease, Renal and Urological Disease (score = 24). Insulin, hydrogen peroxide and ribosomal protein S15 (RPS15) constituted some of the major central nodes in the first network; whereas tumour protein p53 (TP53) constituted a central node in the second network.

IPA Core Analysis was performed for the significantly DE miRNAs, in each RCC subtype and in UT-UC. The log_2_ ratio was set at 0.3 (range –1.9155 to 1.1864) and the DE miRNAs were filtered down to 61 for ccRCC, 48 for papRCC, 44 for chRCC and 77 for UT-UC. The top biological functions and the major miRNA networks are shown in **Figure 12**.

### Target prediction and functional analysis of anti-correlated mRNA/miRNA pairs

From the GEO datasets with accession numbers GSE6344, GSE4125, GSE15641, GSE781 and GSE6280 we isolated the top co-up- and down-regulated genes in ccRCC. We also isolated the top co-upregulated and top co-downregulated microRNAs according to our microarray analysis in ccRCC. Using the miRWalk algorithm (http://www.ma.uni-heidelberg.de/apps/zmf/mirwalk) and four other prediction algorithms (TargetScan, miRanda, miRDB and Diana-microT) we identified the top anti-correlated mRNA/miRNA pairs, with a seed length (SL) of 10 nucleotides or more and a p<0.001. Using this kind of analysis we concluded to the following anti-correlated mRNA/miRNA pairs: ABCA1/miR-23c (SL = 10; p = 0.0032), ATP2B4/miR-520a-5p (SL = 10; p = 0.004) and NR3C1/miR-513a-3p (SL = 11; p = 0.0009) (up-regulated mRNA/down-regulated miRNA); and TFCP2L1/miR-489 (SL = 10; p = 0.0074), TRIM10/miR-874 (SL = 10; p = 0.002), SLCA7A8/miR-210 (SL = 10; p = 0.004) (down-regulated mRNA/up-regulated miRNA).

**Figure 13:**
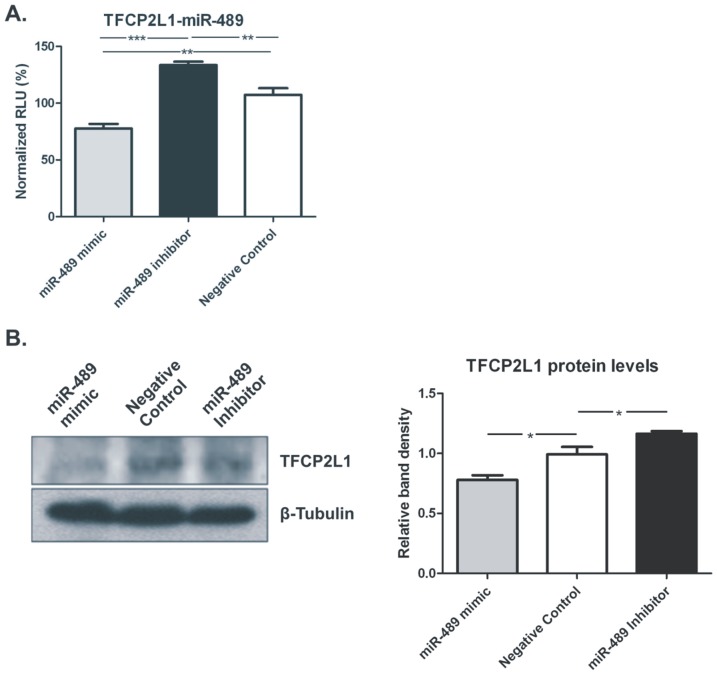
Western blotting. A. Normalized luciferase relative light units (RLUs) in AB8/13 cell lysates after transfection with sensor constructs. Co-transfection of pMIR-REPORT-TFCP2L1 with either miR-489 mimic or inhibitor resulted in significant reduction or increase in luciferase expression versus the negative control, respectively, indicating that miR-489 binds directly on the 3′UTR of TFCP2L1 (^***^, p<0.001; ^**^p<0.05, ANOVA). B. Western blot of TFCP2L1 from AB8/13 cells after transient transfection with miR-489 miRNA LNA mimics, Inhibitors and the AllStars™ Negative Control scrambled sequence LNA. This is a representative of three experiments.The statistical analysis of western blot densitometry results, normalized against the Negative Control is also depicted. Values represent the mean ± SEM. Results illustrate the reduction of TFCP2L1 protein levels at the presence of miR-489 mimics (^*^, p = 0.026), while miRNA Inhibitors significantly increased TFCP2L1 levels (^*^, p = 0.039).

The AB8/13 undifferentiated podocyte cells were incubated at 33°C at 5% CO2 and cultured in RPMI medium, supplemented with 10% Fetal Bovine Serum (FBS), 1% of 100 units/ml Penicillin/Streptomycin and 1% Insulin-Transferrin-Selenium (ITS). Endogenous expression of miR-23c, miR-520a-5p, miR-513a-3p, miR-489, miR-874 and miR-210 in undifferentiated AB8/13 podocyte cells was verified by qRT-PCR. A segment of the 3′UTR of the top co-deregulated genes was cloned into the 3′UTR of the luciferase gene in pMIR-REPORT plasmid (pMIR-REPORT-Gene of interest). Luciferase reporter plasmids and β-gal reference plasmid were co-transfected in AB8/13 podocyte cell line with the corresponding miRNA mimic, miRNA inhibitor or negative control mimics for 16 hours followed by luciferase and β-gal measurement. Co-transfection of pMIR-REPORT-TFCP2L1 with either miR-489 mimic or inhibitor resulted in significant reduction or increase in luciferase expression versus the negative control, respectively, indicating that miR-489 binds directly on the 3′UTR of TFCP2L1 (p<0.001; ANOVA). In agreement, transfection of miR-489 mimics in AB8/13 cells significantly reduced the endogenous levels of TFCP2L1 protein ∼21% of total expression; while miR-489 inhibitors augmented TFCP2L1 levels by reducing the endogenously expressed miRNA level by ∼17% (p<0.001; ANOVA) (**Figure 13**).

## Discussion

RCC subtype classification based on gene expression largely reiterates the one based on the histological appearance. However, miRNA expression profiles have shown that microRNAs are better indicators for distinguishing cancer from normal tissues, and can successfully classify even poorly differentiated tumours [30]. In the present study we analyzed the global microRNA expression of the three most common RCC subtypes along with UT-UC and we compared their miRNA profiles with the corresponding profiles from normal kidney tissue. The majority of the deregulated miRNAs was down-regulated in all RCC subtypes and UT-UC, a pattern consistent with miRNA expression profiles in human cancers [30]. Our hierarchical clustering also showed that RCC and UT-UC tumors could be distinguished perfectly from the normal kidney tissue. Given that RCCs and UT-UCs are distinct tumour entities, it was not surprising that they formed clearly separated clusters. Apart from one chRCC sample which was more heterogeneous and shared equal miRNA expression pattern with several ccRCC samples, each RCC subtype exhibited a distinct miRNA profile (**Figure 3**). Furthermore, in agreement with previous reports [5], [16], [20], ccRCC and papRCC subtypes were more closely related when compared with chRCC, reflecting their common origin in the proximal tubule of the renal cortex.

Using a strict filtering approach in the ROC tests, following qRT-PCR validation for the top deregulated miRNAs in each tumor entity, we concluded in a group of miRNAs that were also validated in an independent patient cohort. Therefore, we suggest that these miRNAs could be successfully used as distinguishing markers for each RCC subtype or UT-UC. We showed that miR-3648, miR-489, miR-638, miR-3656, miR-3687, miR-25-5p, miR-21-5p and miR-663b have significant potential diagnostic value for ccRCC. Moreover, miR-3648, miR-638, miR-3656, miR-3687, miR-25-5p and miR-656 were the best markers for papRCC distinction; whereas miR-3687, miR-4284 and miR-141-3p can accurately discriminate chRCC over the normal kidney. These results were also confirmed using linear regression analysis, where those miRNAs manifested both differential expression and linear behavior between RCC, UT-UC and the normal kidney. However, despite the strict filtering approach that we followed, a validation in a larger papRCC, chRCC and UT-UC sample number would strengthen the diagnostic power of the latter miRNAs. It is particularly important to note that several validated miRNAs were suggested as discriminatory markers in more than one tumour entities and especially miR-3687 could discriminate evenly all RCC subtypes and UT-UC vs. the normal kidney, as also shown from our regression analysis. This finding denotes that deregulation of miR-3687 seems to be a common manifestation during the renal carcinogenic process. Similar miRNA panels were recently used by others in order to distinguish various RCC subtypes from the normal kidney; however the results are not always consistent among them [12], [13], [16], [17], [19], [20], [23], [24], [31], [32]. This can be attributed to various factors, such as different microarray platforms used, different normalization methods followed, different sample number, etc. Spector et al. just recently developed a similar miRNA-based diagnostic assay for the classification of ccRCC, papRCC, chRCC and benign oncocytoma. Following a similar approach, they validated a classifier set of 24 miRNAs to an independent cohort and they distinguished miR-210, miR-195, miR-182, miR-31-3p and miR-551b, among others [33]. Interestingly enough, these miRNAs were also included among the top 30 deregulated miRNAs in our study (**Table S2**). Apart from the recent study of Izquierdo et al. [34], where the authors investigated miRNA expression in progressing and non-progressing UT-UC patients, this is the first report to our knowledge suggesting a group of miRNAs able to discriminate UT-UC from the normal kidney. Despite the small UT-UC sample number examined, our data show that miR-3648, miR-638, miR-3656 and miR-3687 were the best molecular markers for distinction of UT-UC over the normal kidney (**Table 3**). We also compared the data between UT-UC and RCC subtypes, and identified miRNAs that were specific for each tumour entity. Among them 16 miRNAs were specifically down-regulated and 5 miRNAs were specifically up-regulated in UT-UC (**Table S3**). Approximately 50% of all annotated human miRNA genes are located at fragile sites or areas of the genome that are associated with cancer which are prone to breakage and rearrangement in cancer cells [35], [36], [37], [38], [39], [40], [41], [42], [43], [44]. Our data showed a partial matching pattern between the reported cytogenetic changes and miRNA deregulation in RCC and UT-UC. This is in agreement with the recent findings of Chow et al. who demonstrated that chromosomal aberrations in ccRCC can control the deregulation of miRNAs [12].

It was not surprising that IPA revealed that the most significant biological functions of the deregulated miRNAs in RCC involved cancer and renal and urological disease. Furthermore, the systemic inflammatory response has been revealed to contribute to tumorigenesis in the last two decades. Key inflammatory pathways, such as the NF-κB [45], AP-1 [46] and STAT3 [47] pathways, are implicated in the proliferation, transformation, survival, invasion, angiogenesis, metastasis, chemoresistance and radioresistance of cancer [48]. Systemic inflammatory response, represented by elevated C-reactive protein (CRP), platelet count (PC) and erythrocyte sedimentation rate (ESR) was recently shown to predict poor survival in patients with RCC [49]. The tumor suppressor p53 (TP53) and v-myc avian myelocytomatosis viral oncogene homolog (MYC) constituted central nodes in most miRNA networks. TP53 plays a central role in tumor prevention. As a transcription factor, it mainly exerts its function through transcription regulation of its target genes to initiate various cellular responses. Recent studies have demonstrated that TP53 cross-talks with miRNAs at multiple levels; from inducing their transcription expression and promoting their maturation, to being negatively regulated by them [50]. MYC expression was recently shown to be significantly upregulated in RCC [51] and its implication with miRNAs in RCC was also recently reported [52]. As a transcription factor, it was recently shown to regulate genes involved in cell growth, including those regulating ribosome and mitochondrial biogenesis and intermediary metabolism; and glycolytic genes, enhancing glycolysis, glutamine uptake and metabolism, and lactate production [53].

Finally, we isolated the top co-upregulated and top co-downregulated mRNA transcripts from 5 independent GEO ccRCC datasets, and combining our miRNA results by a computational approach, we concluded to the six most anti-correlated mRNA/miRNA pairs the association of which was functionally investigated. We showed that the developmental transcription factor TFCP2L1 (transcription factor CP2-like 1) whose loss was recently suggested to play a role in the loss of epithelial differentiation and function in ccRCC, is a true target of miR-489, which was among the ccRCC-specific miRNAs in our study [54].

The current study adds a number of novel deregulated miRNAs accompanied by the molecular pathways in which they are involved mainly in ccRCC. These miRNAs accurately discriminate ccRCC from the normal kidney and can provide the basis for a specific targeted therapy. Due to the small sample number of the papRCC, chRCC and UT-UC samples that were used in the present study, the respective miRNAs should be carefully taken into consideration.

## Supporting Information

Figure S1 **Pair-wise Hierarchical Clistering (HCl).** Pair-tissue-wise unsupervised two-way hierarchical clustering with Euclidian distance. The log_2_ fold change in each RCC subtype and UT-UC versus the normal kidney tissue was used to construct the heat map. miRNA profiling accurately discriminated between ccRCC and papRCC (A), ccRCC and chRCC (B), chRCC and papRCC (C), ccRCC and UT-UC (D), papRCC and UT-UC (E), and chRCC and UT-UC (F). ccRCC, clear cell renal cell carcinoma; papRCC, papillary renal cell carcinoma; chRCC, chromophobe renal cell carcinoma; UT-UC, upper tract urothelial carcinoma. Red and blue colours show significant up- or down-regulation of each miRNA in the tumour versus the normal kidney, respectively.
(TIF)

Figure S2 **Regression analysis.** Regression analysis of top up-regulated (A) and top down-regulated (B) miRNAs. miRNAs were tested with one-way ANOVA and further linear regression was used to fit their expression into a linear model. Left columns represent the ANOVA results. Right columns represent the linear regression results after sorting of the median values of the respective miRNAs in ascending order.
(DOCX)

Figure S3 **Regression analysis.** Regression analysis of top up-regulated (A) and top down-regulated (B) miRNAs. miRNAs were tested with one-way ANOVA and further linear regression was used to fit their expression into a linear model. Left columns represent the ANOVA results. Right columns represent the linear regression results after sorting of the median values of the respective miRNAs in ascending order.
(DOCX)

Figure S4 **Regression analysis.** Regression analysis of the top deregulated miRNAs. One-way ANOVA (left columns) along with linear regression (right columns) of the RCC grades with respect to miRNA expression was used to fit miRNA expression into a linear model. Each regression consists of two curves: one of the form y = ax+b (red) and one of the form y = ax3+bx2+cx+d (blue). All results presented obtained a p<0.05. The median expression values of the miRNAs were used. Interestingly, miR-148b-5p, miR-1910 and miR-200b-5p, miR-210 and miR-3656, presented linear behavior in a descending order of the RCC grade; whereas miR-3687 and miR-654-5p manifested linear behavior in ascending order of the RCC grade.
(DOC)

Figure S5 **Multiple sequence alignments (MSA).** Representative MSA and phylogenetic trees of miR-489 (A) and miR-25-5p (B). The majority of the DE miRNAs (78.45%) were highly conserved among species. Specifically, 70.68% of the up-regulated and 81.22% of the down-regulated miRNAs were phylogenetically conserved among species.
(TIF)

Table S1 **The table lists the primer sequences that were designed to introduce a SpeI and a HindIII restriction enzyme sites to be cloned into the pMiR-REPORT luciferase vector.**
(DOC)

Table S2 **Top deregulated miRNAs in ccRCC, papRCC, chRCC and UT-UC vs. the normal kidney tissue.**
(DOC)

Table S3 **Co-deregulated and tissue specific miRNAs in ccRCC, papRCC, chRCC and UT-UC.** Co-upregulated and tissue specific miRNAs in RCC subtypes (A). Co-downregulated and tissue specific miRNAs in RCC subtypes (B). Co-upregulated and tissue specific miRNAs in RCC and UT-UC (C). Co-downregulated and tissue specific miRNAs in RCC and UT-UC (D).
(DOC)

Table S4 **Reported chromosomal abnormalities associated with ccRCC, papRCC, chRCC and UT-UC, as well as some of the well-studied genes located within those aberrated regions that are involved in the pathogenesis of the tumour subtypes.** Many of the significantly deregulated miRNAs are located within cytogenetic regions that are significantly aberrated in the different subtypes. In the reported chromosomal aberrations the minus sign denotes a loss and the plus sign denotes a gain in chromosomal material. Red colour (↑) denotes elevated miRNA expression and blue colour (↓) denotes reduced miRNA expression.
(DOC)

## References

[1] Meloni-Ehrig AM (2002) Renal cancer: cytogenetic and molecular genetic aspects. Am J Med Genet 115: 164–172. 12407697 10.1002/ajmg.10697

[2] Ficarra V, Martignoni G, Galfano A, Novara G, Gobbo S, et al (2006) Prognostic role of the histologic subtypes of renal cell carcinoma after slide revision. Eur Urol 50: 786–793. 16697521 10.1016/j.eururo.2006.04.009

[3] Lopez-Beltran A, Kirkali Z, Cheng L, Egevad L, Regueiro JC, et al (2008) Targeted therapies and biological modifiers in urologic tumors: pathobiology and clinical implications. Semin Diagn Pathol 25: 232–244. 19013890 10.1053/j.semdp.2008.07.006

[4] Tazi el M, Essadi I, Tazi MF, Ahellal Y, M'Rabti H, et al (2011) Advanced treatments in non-clear renal cell carcinoma. Urol J 8: 1–11. 21404194

[5] Youssef YM, White NMA, Grigull J, Krizova A, Samy C, et al (2011) Accurate molecular classification of kidney cancer subtypes using microRNA signature. Eur Urol 59: 721–730. 21272993 10.1016/j.eururo.2011.01.004

[6] Fridman E, Dotan Z, Barshack I, David MB, Dov A, et al (2010) Accurate molecular classification of renal tumors using microRNA expression. J Mol Diagn 12: 687–696. 20595629 10.2353/jmoldx.2010.090187 PMC2928434

[7] Higgins JP, Kaygusuz G, Wang L, Montgomery K, Mason V, et al (2007) Placental S100 (S100P) and GATA3: markers for transitional epithelium and urothelial carcinoma discovered by complementary DNA microarray. Am J Surg Pathol 31: 673–680. 17460449 10.1097/01.pas.0000213438.01278.5f

[8] Roupret M, Zigeuner R, Palou J, Boehle A, Kaasinen E, et al (2011) European guidelines for the diagnosis and management of upper urinary tract urothelial cell carcinomas: 2011 update. Eur Urol 59: 584–594. 21269756 10.1016/j.eururo.2010.12.042

[9] Kirkali Z, Tuzel E (2003) Transitional cell carcinoma of the ureter and renal pelvis. Crit Rev Oncol Hematol 47: 155–169. 12900009 10.1016/s1040-8428(03)00079-9

[10] Garzon R, Pichiorri F, Palumbo T, Visentini M, Aqeilan R, et al (2007) MicroRNA gene expression during retinoic acid-induced differentiation of human acute promyelocytic leukemia. Oncogene 26: 4148–4157. 17260024 10.1038/sj.onc.1210186

[11] Esquela-Kerscher A, Slack FJ (2006) Oncomirs - microRNAs with a role in cancer. Nat Rev Cancer 6: 259–269. 16557279 10.1038/nrc1840

[12] Chow TF, Youssef YM, Lianidou E, Romaschin AD, Honey RJ, et al (2010) Differential expression profiling of microRNAs and their potential involvement in renal cell carcinoma pathogenesis. Clin Biochem 43: 150–158. 19646430 10.1016/j.clinbiochem.2009.07.020

[13] White NM, Bao TT, Grigull J, Youssef YM, Girgis A, et al (2011) miRNA profiling for clear cell renal cell carcinoma: biomarker discovery and identification of potential controls and consequences of miRNA dysregulation. J Urol 186: 1077–1083. 21784468 10.1016/j.juro.2011.04.110

[14] Zaravinos A, Radojicic J, Lambrou GI, Volanis D, Delakas D, et al (2012) Expression of miRNAs involved in angiogenesis, tumor cell proliferation, tumor suppressor inhibition, epithelial-mesenchymal transition and activation of metastasis in bladder cancer. J Urol 188: 615–623. 22704449 10.1016/j.juro.2012.03.122

[15] Chow TF, Youssef YM, Lianidou E, Romaschin AD, Honey RJ, et al (2010) Differential expression profiling of microRNAs and their potential involvement in renal cell carcinoma pathogenesis. Clin Biochem 43: 150–158. 19646430 10.1016/j.clinbiochem.2009.07.020

[16] Fridman E, Dotan Z, Barshack I, Ben David M, Dov A, et al (2010) Accurate molecular classification of renal tumors using microRNA expression. J Mol Diagn 12: 687–696. 20595629 10.2353/jmoldx.2010.090187 PMC2928434

[17] Jung M, Mollenkopf HJ, Grimm C, Wagner I, Albrecht M, et al (2009) MicroRNA profiling of clear cell renal cell cancer identifies a robust signature to define renal malignancy. J Cell Mol Med 13: 3918–3928. 19228262 10.1111/j.1582-4934.2009.00705.x PMC4516539

[18] Nakada C, Matsuura K, Tsukamoto Y, Tanigawa M, Yoshimoto T, et al (2008) Genome-wide microRNA expression profiling in renal cell carcinoma: significant down-regulation of miR-141 and miR-200c. J Pathol 216: 418–427. 18925646 10.1002/path.2437

[19] Osanto S, Qin Y, Buermans HP, Berkers J, Lerut E, et al (2012) Genome-wide microRNA expression analysis of clear cell renal cell carcinoma by next generation deep sequencing. PLoS One 7: e38298. 22745662 10.1371/journal.pone.0038298 PMC3380046

[20] Petillo D, Kort EJ, Anema J, Furge KA, Yang XJ, et al (2009) MicroRNA profiling of human kidney cancer subtypes. Int J Oncol 35: 109–114. 19513557 10.3892/ijo_00000318

[21] Radojicic J, Zaravinos A, Vrekoussis T, Kafousi M, Spandidos DA, et al (2011) MicroRNA expression analysis in triple-negative (ER, PR and Her2/neu) breast cancer. Cell Cycle 10: 507–517. 21270527 10.4161/cc.10.3.14754

[22] Gottardo F, Liu CG, Ferracin M, Calin GA, Fassan M, et al (2007) Micro-RNA profiling in kidney and bladder cancers. Urol Oncol 25: 387–392. 17826655 10.1016/j.urolonc.2007.01.019

[23] Huang Y, Dai Y, Yang J, Chen T, Yin Y, et al (2009) Microarray analysis of microRNA expression in renal clear cell carcinoma. Eur J Surg Oncol 35: 1119–1123. 19443172 10.1016/j.ejso.2009.04.010

[24] Juan D, Alexe G, Antes T, Liu H, Madabhushi A, et al (2010) Identification of a microRNA panel for clear-cell kidney cancer. Urology 75: 835–841. 20035975 10.1016/j.urology.2009.10.033

[25] Yi Z, Fu Y, Zhao S, Zhang X, Ma C (2010) Differential expression of miRNA patterns in renal cell carcinoma and nontumorous tissues. J Cancer Res Clin Oncol 136: 855–862. 19921256 10.1007/s00432-009-0726-x PMC11827771

[26] Sun Y, Koo S, White N, Peralta E, Esau C, et al (2004) Development of a micro-array to detect human and mouse microRNAs and characterization of expression in human organs. Nucleic Acids Res 32: e188. 15616155 10.1093/nar/gnh186 PMC545483

[27] Zaravinos A, Bizakis J, Soufla G, Sourvinos G, Spandidos DA (2007) Mutations and differential expression of the ras family genes in human nasal polyposis. Int J Oncol 31: 1051–1059. 17912430

[28] Ljungberg B, Cowan NC, Hanbury DC, Hora M, Kuczyk MA, et al (2010) EAU guidelines on renal cell carcinoma: the 2010 update. Eur Urol 58: 398–406. 20633979 10.1016/j.eururo.2010.06.032

[29] Roupret M, Zigeuner R, Palou J, Boehle A, Kaasinen E, et al (2012) [European guidelines for the diagnosis and management of upper urinary tract urothelial cell carcinomas: 2011 update. European Association of Urology Guideline Group for urothelial cell carcinoma of the upper urinary tract]. Actas Urol Esp 36: 2–14. 22036956 10.1016/j.acuro.2011.09.001

[30] Lu J, Getz G, Miska EA, Alvarez-Saavedra E, Lamb J, et al (2005) MicroRNA expression profiles classify human cancers. Nature 435: 834–838. 15944708 10.1038/nature03702

[31] Nakada C, Matsuura K, Tsukamoto Y, Tanigawa M, Yoshimoto T, et al (2008) Genome-wide microRNA expression profiling in renal cell carcinoma: significant down-regulation of miR-141 and miR-200c. J Pathol 216: 418–427. 18925646 10.1002/path.2437

[32] Weng L, Wu X, Gao H, Mu B, Li X, et al (2010) MicroRNA profiling of clear cell renal cell carcinoma by whole-genome small RNA deep sequencing of paired frozen and formalin-fixed, paraffin-embedded tissue specimens. J Pathol 222: 41–51. 20593407 10.1002/path.2736

[33] Spector Y, Fridman E, Rosenwald S, Zilber S, Huang Y, et al (2013) Development and validation of a microRNA-based diagnostic assay for classification of renal cell carcinomas. Mol Oncol 7: 732–738. 23587442 10.1016/j.molonc.2013.03.002 PMC5528479

[34] Izquierdo L, Ingelmo-Torres M, Mallofre C, Lozano JJ, Verhasselt-Crinquette M, et al.. (2013) Prognostic value of microRNA expression pattern in upper tract urothelial carcinoma. BJU Int. 10.1111/bju.12551 24180461

[35] Calin GA, Sevignani C, Dumitru CD, Hyslop T, Noch E, et al (2004) Human microRNA genes are frequently located at fragile sites and genomic regions involved in cancers. Proc Natl Acad Sci U S A 101: 2999–3004. 14973191 10.1073/pnas.0307323101 PMC365734

[36] Girgis AH, Iakovlev VV, Beheshti B, Bayani J, Squire JA, et al (2012) Multilevel whole-genome analysis reveals candidate biomarkers in clear cell renal cell carcinoma. Cancer Res 72: 5273–5284. 22926558 10.1158/0008-5472.CAN-12-0656

[37] Bratslavsky G, Sudarshan S, Neckers L, Linehan WM (2007) Pseudohypoxic pathways in renal cell carcinoma. Clin Cancer Res 13: 4667–4671. 17699843 10.1158/1078-0432.CCR-06-2510

[38] Klatte T, Rao PN, de Martino M, LaRochelle J, Shuch B, et al (2009) Cytogenetic profile predicts prognosis of patients with clear cell renal cell carcinoma. J Clin Oncol 27: 746–753. 19124809 10.1200/JCO.2007.15.8345

[39] Kovacs G, Fuzesi L, Emanual A, Kung HF (1991) Cytogenetics of papillary renal cell tumors. Genes Chromosomes Cancer 3: 249–255. 1958590 10.1002/gcc.2870030403

[40] Speicher MR, Schoell B, du Manoir S, Schrock E, Ried T, et al (1994) Specific loss of chromosomes 1, 2, 6, 10, 13, 17, and 21 in chromophobe renal cell carcinomas revealed by comparative genomic hybridization. Am J Pathol 145: 356–364. 7519827 PMC1887405

[41] Storkel S, Eble JN, Adlakha K, Amin M, Blute ML, et al (1997) Classification of renal cell carcinoma: Workgroup No. 1. Union Internationale Contre le Cancer (UICC) and the American Joint Committee on Cancer (AJCC). Cancer 80: 987–989. 9307203 10.1002/(sici)1097-0142(19970901)80:5<987::aid-cncr24>3.0.co;2-r

[42] van den Berg E, Dijkhuizen T, Oosterhuis JW, Geurts van Kessel A, de Jong B, et al (1997) Cytogenetic classification of renal cell cancer. Cancer Genet Cytogenet 95: 103–107. 9140459 10.1016/s0165-4608(96)00289-0

[43] Velickovic M, Delahunt B, Storkel S, Grebem SK (2001) VHL and FHIT locus loss of heterozygosity is common in all renal cancer morphotypes but differs in pattern and prognostic significance. Cancer Res 61: 4815–4819. 11406557

[44] Yoshimoto T, Matsuura K, Karnan S, Tagawa H, Nakada C, et al (2007) High-resolution analysis of DNA copy number alterations and gene expression in renal clear cell carcinoma. J Pathol 213: 392–401. 17922474 10.1002/path.2239

[45] Thapa RJ C, Cheung M, Nogusa S, Pei J, Peri S, et al (2013) NF-κB inhibition by bortezomib permits IFN-γ-activated RIP1 kinase-dependent necrosis in renal cell carcinoma. Mol Cancer Ther 12: 1568–1578. 23657944 10.1158/1535-7163.MCT-12-1010 PMC3746800

[46] Sato A, Nagase H, Obinata D, Fujiwara K, Fukuda N, et al (2013) Inhibition of MMP-9 using a pyrrole-imidazole polyamide reduces cell invasion in renal cell carcinoma. Int J Oncol 43: 1441–1446. 23969530 10.3892/ijo.2013.2073

[47] Zaravinos A, Pieri M, Mourmouras N, Anastasiadou N, Zouvani I, et al.. (2014) Altered metabolic pathways in clear cell renal cell carcinoma: A meta-analysis and validation study focused on the deregulated genes and their associated networks. Oncoscience, Advance Publications. 10.18632/oncoscience.13 PMC4278286 25594006

[48] Aggarwal BB, Gehlot P (2009) Inflammation and cancer: how friendly is the relationship for cancer patients? Curr Opin Pharmacol 9: 351–369. 19665429 10.1016/j.coph.2009.06.020 PMC2730981

[49] Wu Y, Fu X, Zhu X, He X, Zou C, et al (2011) Prognostic role of systemic inflammatory response in renal cell carcinoma: a sytemic review and meta-analysis. J Cancer Res Clin Oncol 137: 887–896. 20878529 10.1007/s00432-010-0951-3 PMC11828339

[50] Feng Z, Zhang C, Wu R, Hu W (2011) Tumor suppressor p53 meets microRNAs. J Mol Cell Biol 3: 44–50. 21278451 10.1093/jmcb/mjq040 PMC3030969

[51] Yamada Y, Hidaka H, Seki N, Yoshino H, Yamasaki T, et al (2013) Tumor-suppressive microRNA-135a inhibits cancer cell proliferation by targeting the c-MYC oncogene in renal cell carcinoma. Cancer Sci 104: 304–312. 23176581 10.1111/cas.12072 PMC7657112

[52] Wach S, Nolte E, Theil A, Stöhr C, T Rau T, et al (2013) MicroRNA profiles classify papillary renal cell carcinoma subtypes. Br J Cancer 109: 714–722. 23799849 10.1038/bjc.2013.313 PMC3738121

[53] Teicher BA, Linehan WM, Helman LJ (2012) Targeting cancer metabolism. Clin Cancer Res 18: 5537–5545. 23071355 10.1158/1078-0432.CCR-12-2587 PMC3475613

[54] Tun HW, Marlow LA, von Roemeling CA, Cooper SJ, Kreinest P, et al (2010) Pathway signature and cellular differentiation in clear cell renal cell carcinoma. PLoS One 5: e10696. 20502531 10.1371/journal.pone.0010696 PMC2872663

