# Correction: New miRNA Profiles Accurately Distinguish Renal Cell Carcinomas and Upper Tract Urothelial Carcinomas from the Normal Kidney

**DOI:** 10.1371/journal.pone.0100063

**Published:** 2014-06-05

**Authors:** The PLOS ONE Staff

In the XML and PDF versions of the article, the corresponding authors’ email addresses are duplicated. Please see below for the authors’ correct email addresses:

Apostolos Zaravinos, zaravinos.apostolos@ucy.ac.cy

Constantinos Deltas, Deltas@ucy.ac.cy

In the PDF version of the article, the corresponding author’s name is spelled incorrectly in the footnote containing the author’s current address. The correct name is: Apostolos Zaravinos.

The journal apologizes for these errors, which were introduced during the preparation of the article for the publication.
