# Supplementary figures and images for: New miRNA Profiles Accurately Distinguish Renal Cell Carcinomas and Upper Tract Urothelial Carcinomas from the Normal Kidney
Source: PLoS One. 2014 Mar 12;9(3):e91646. doi: 10.1371/journal.pone.0091646 (PMC3951427; doi:10.1371/journal.pone.0091646)

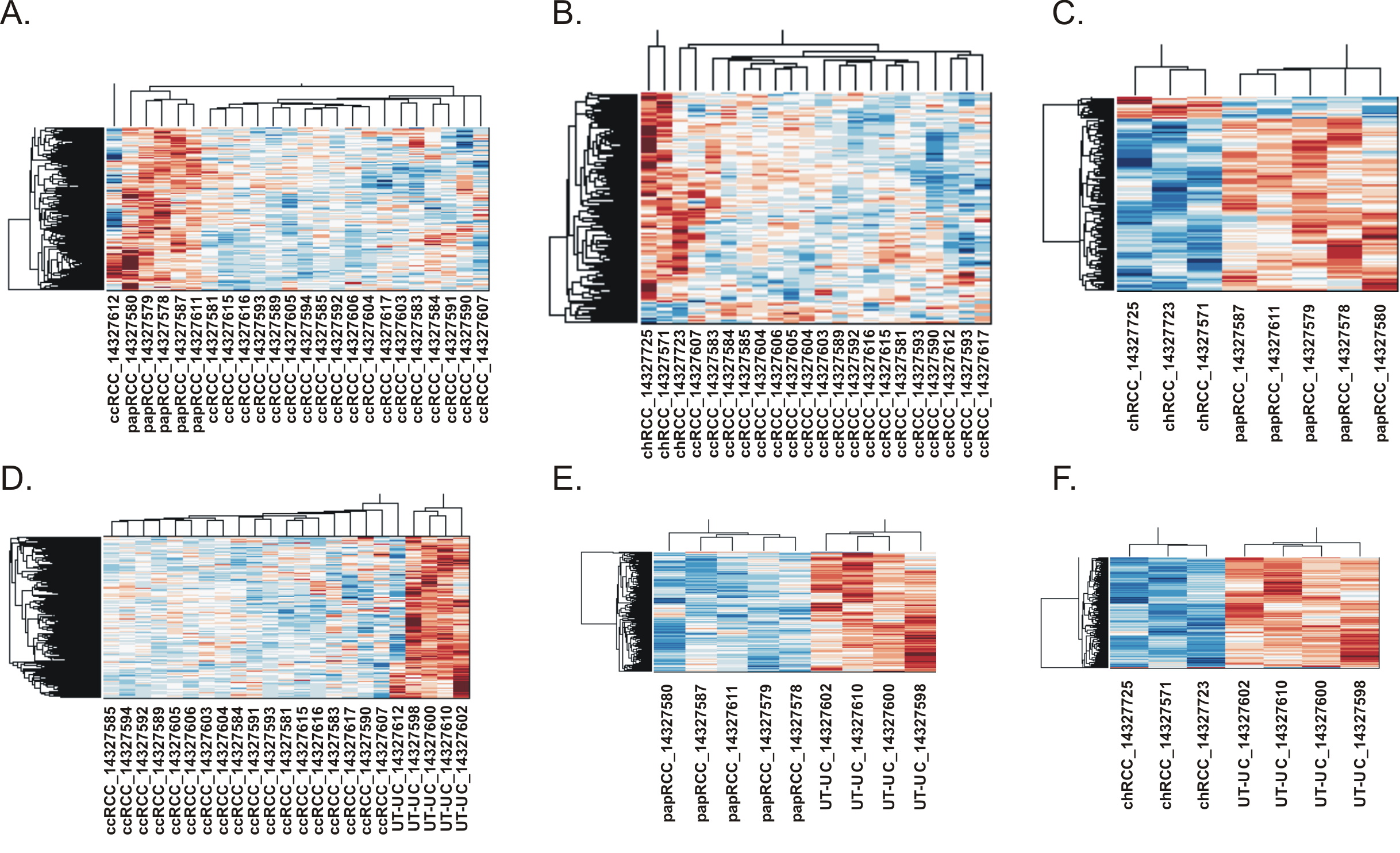

Supplement: Figure S1 — Pair-wise Hierarchical Clistering (HCl). Pair-tissue-wise unsupervised two-way hierarchical clustering with Euclidian distance. The log2 fold change in each RCC subtype and UT-UC versus the normal kidney tissue was used to construct the heat map. miRNA profiling accurately discriminated between ccRCC and papRCC (A), ccRCC and chRCC (B), chRCC and papRCC (C), ccRCC and UT-UC (D), papRCC and UT-UC (E), and chRCC and UT-UC (F). ccRCC, clear cell renal cell carcinoma; papRCC, papillary renal cell carcinoma; chRCC, chromophobe renal cell carcinoma; UT-UC, upper tract urothelial carcinoma. Red and blue colours show significant up- or down-regulation of each miRNA in the tumour versus the normal kidney, respectively. (TIF) [file pone.0091646.s001.tif]

**A)**


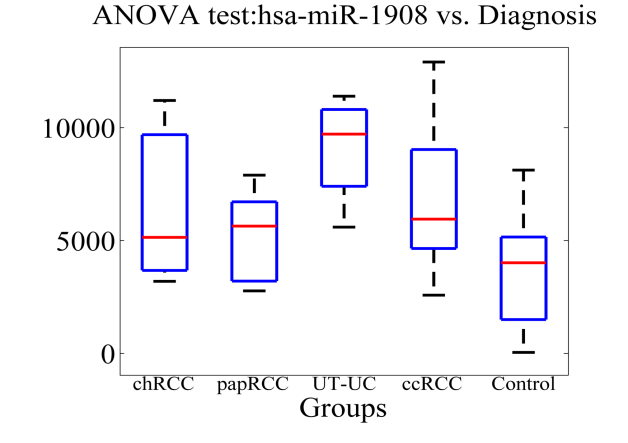

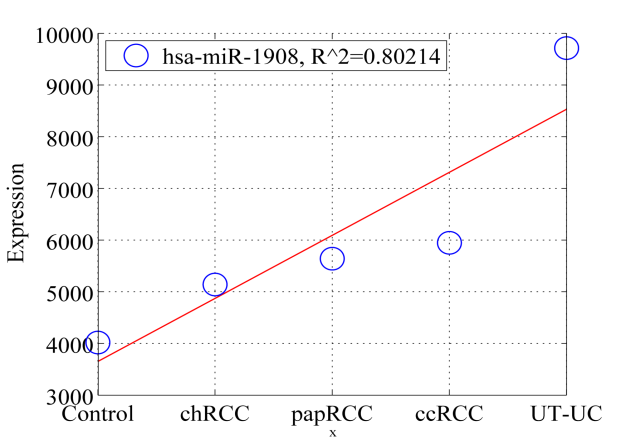

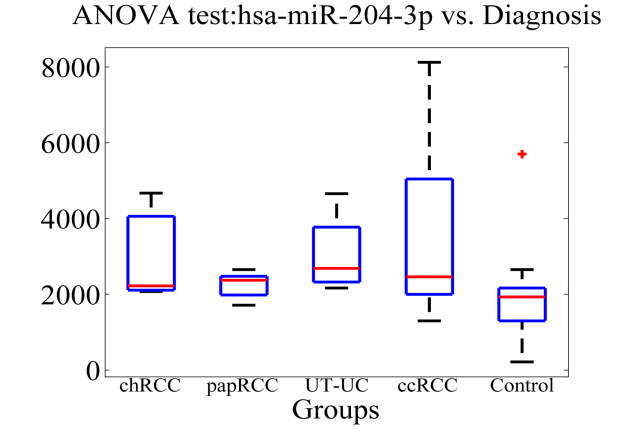

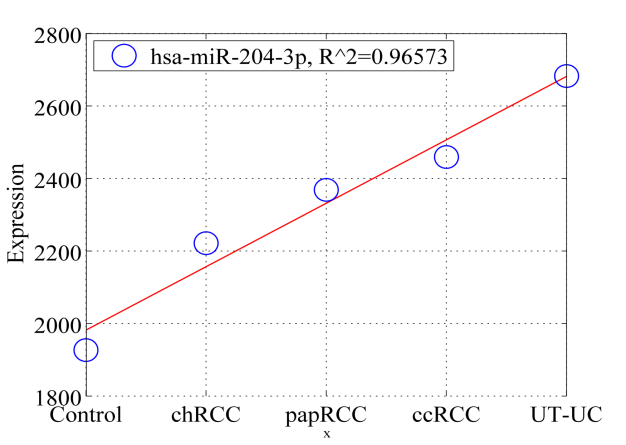

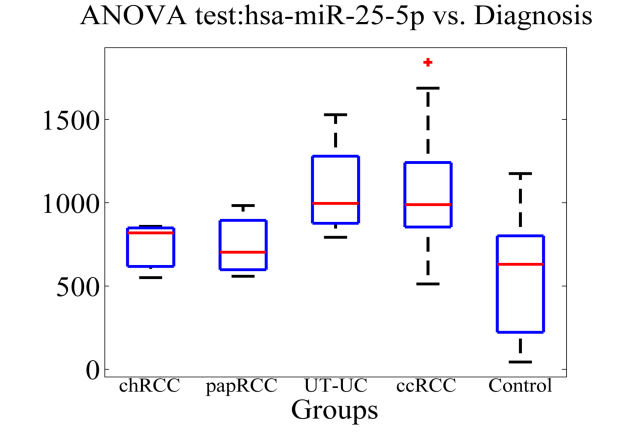

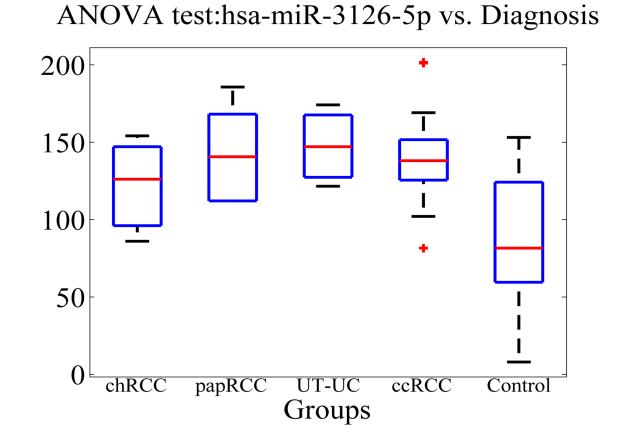

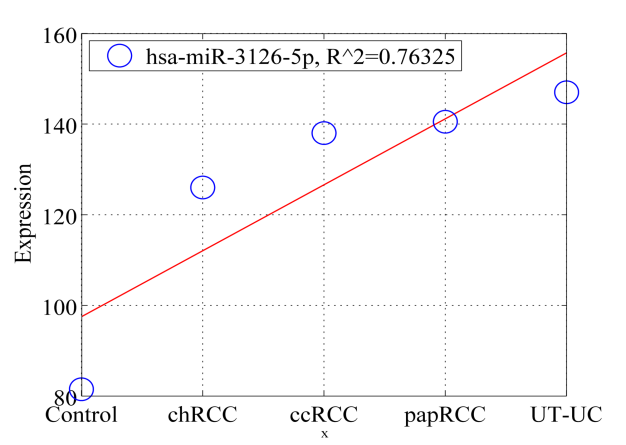

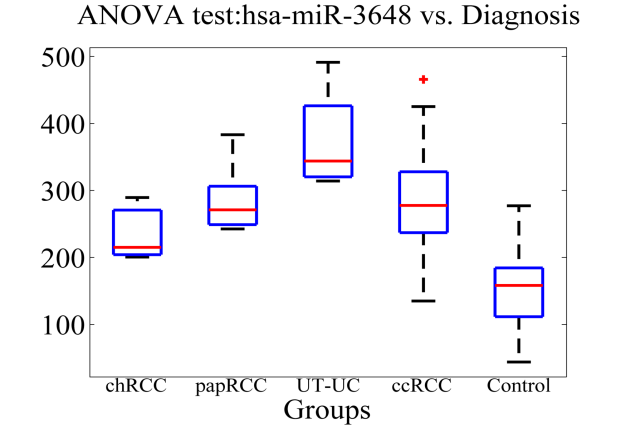

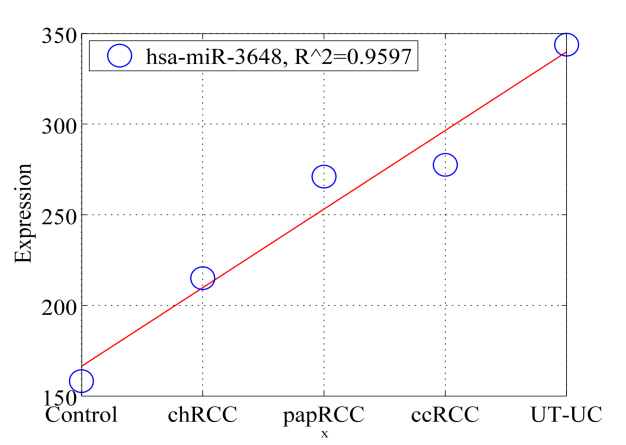

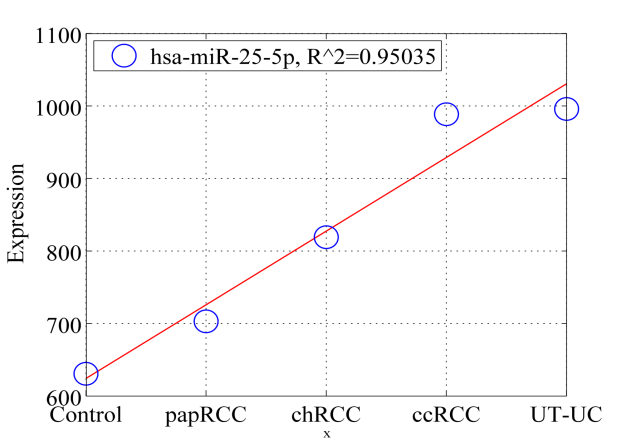

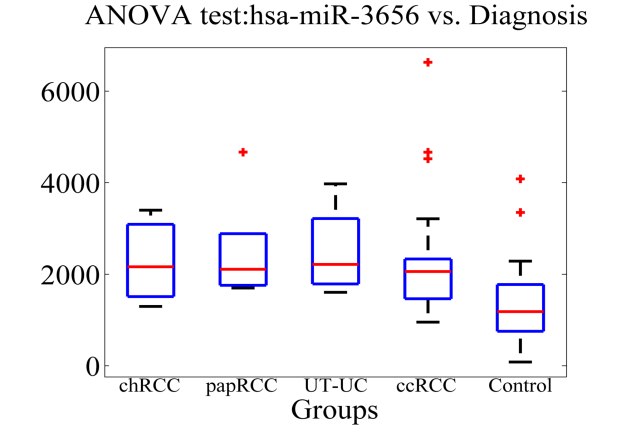

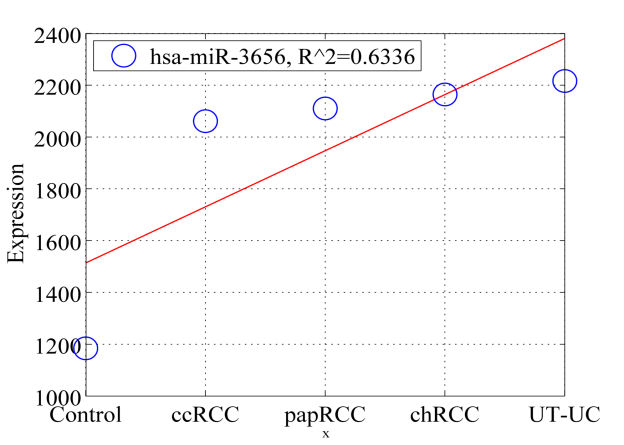

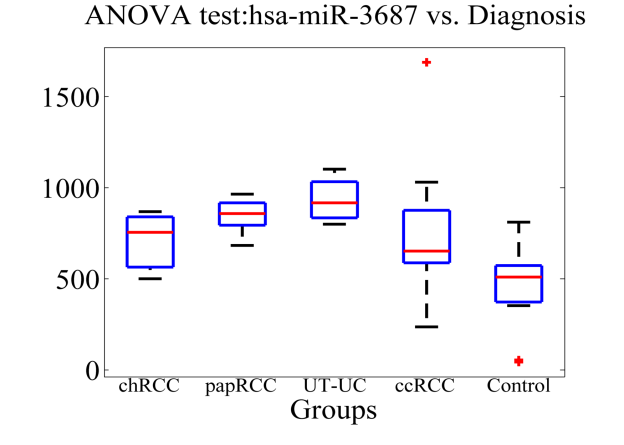

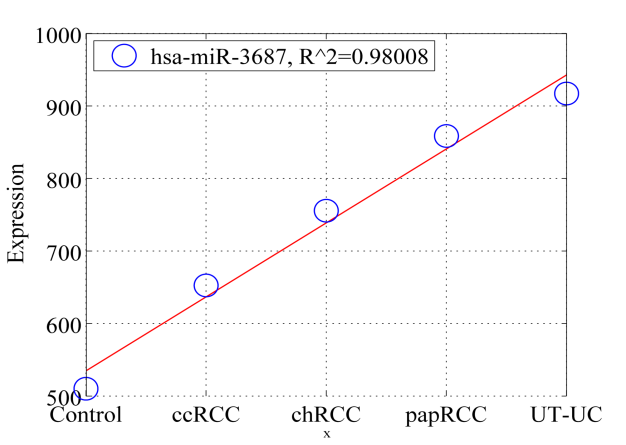

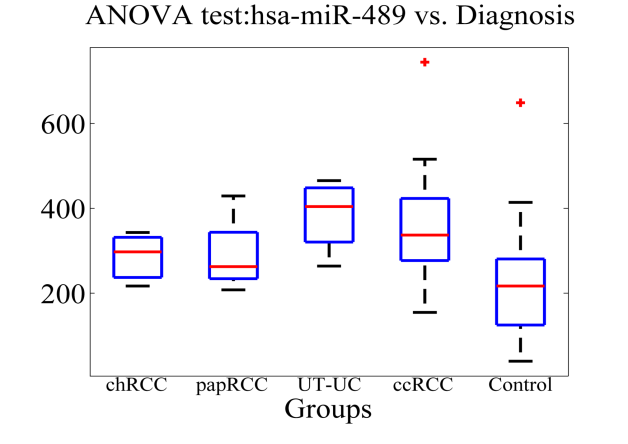

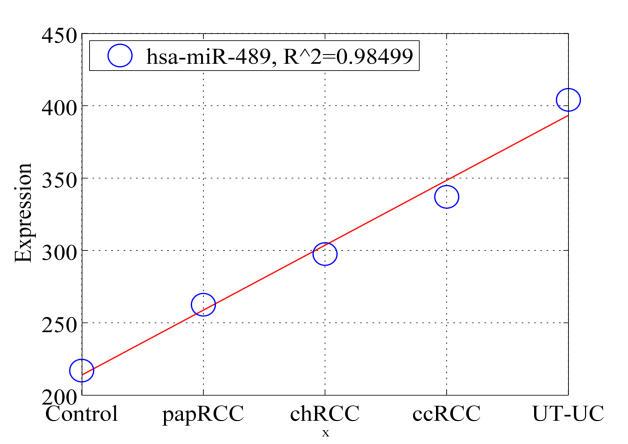

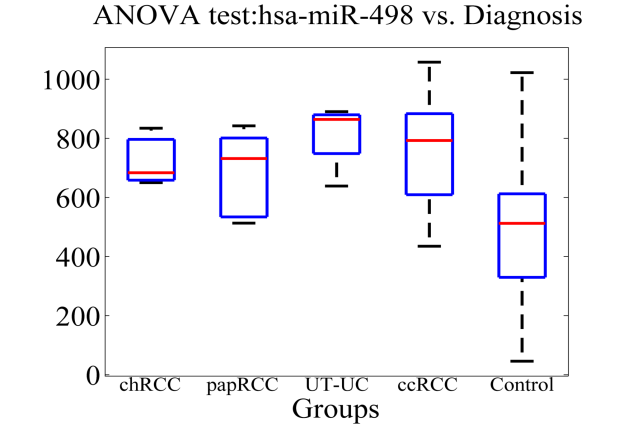

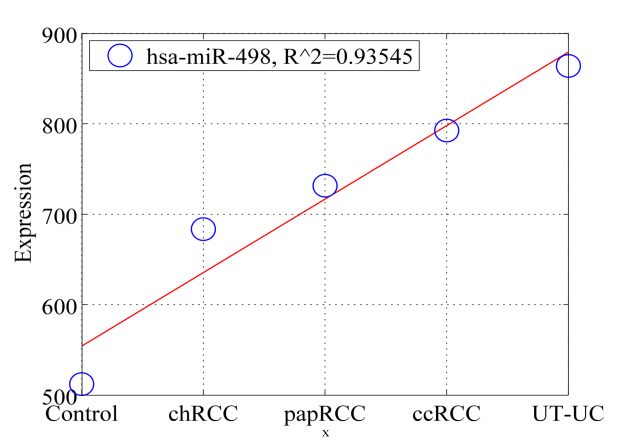

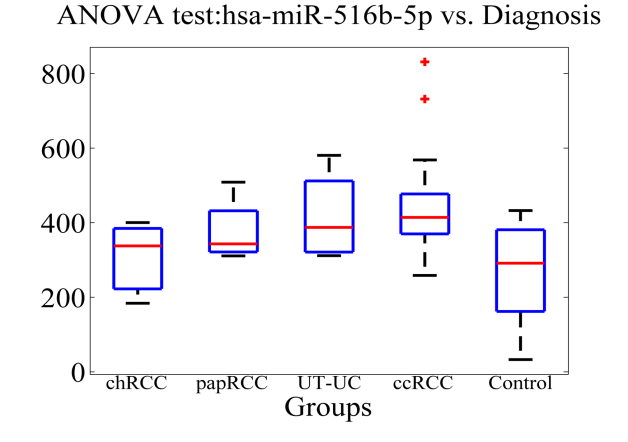

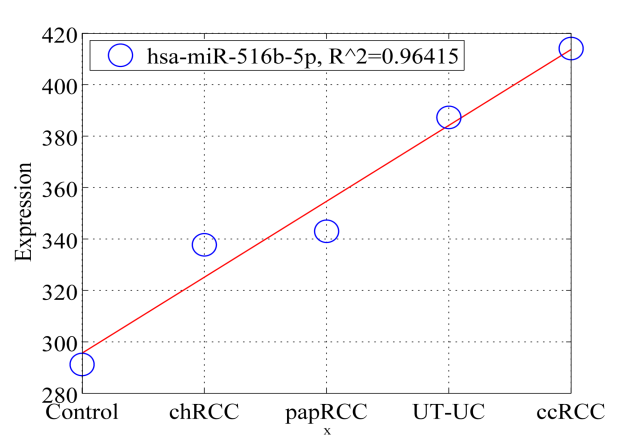

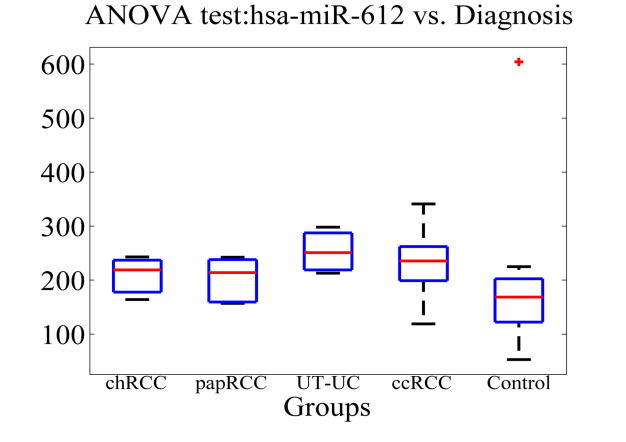

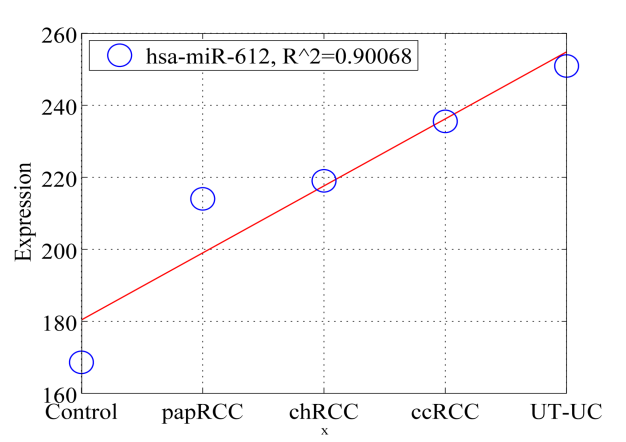

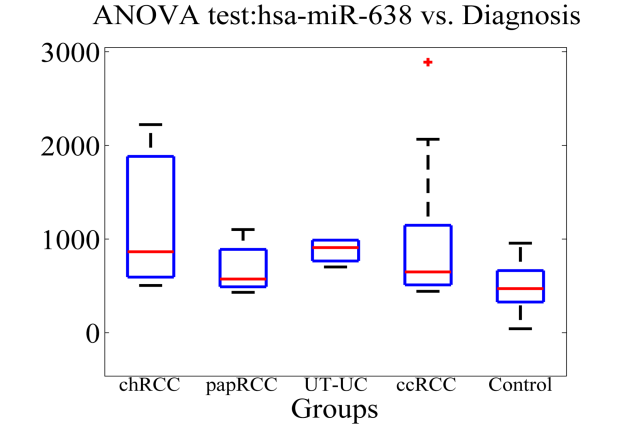

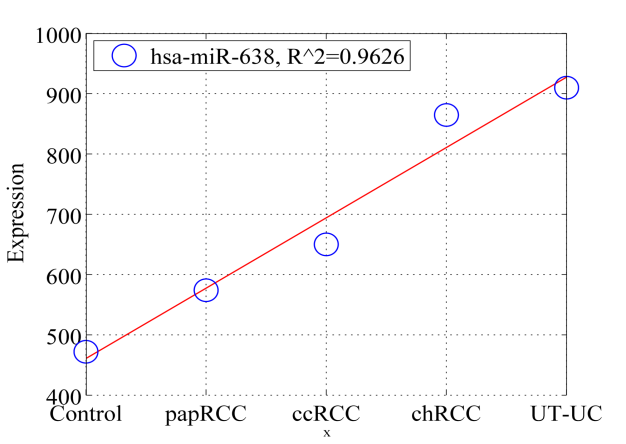

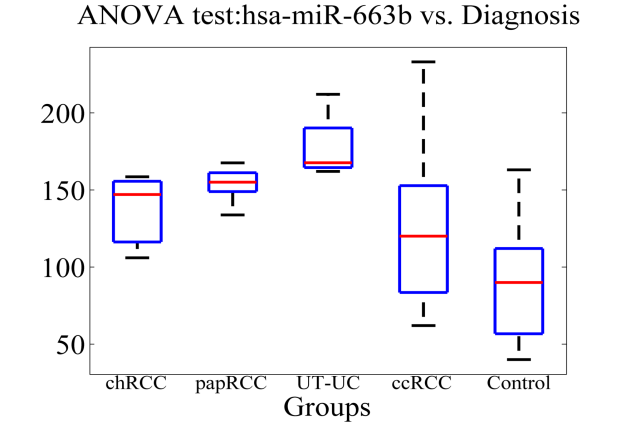

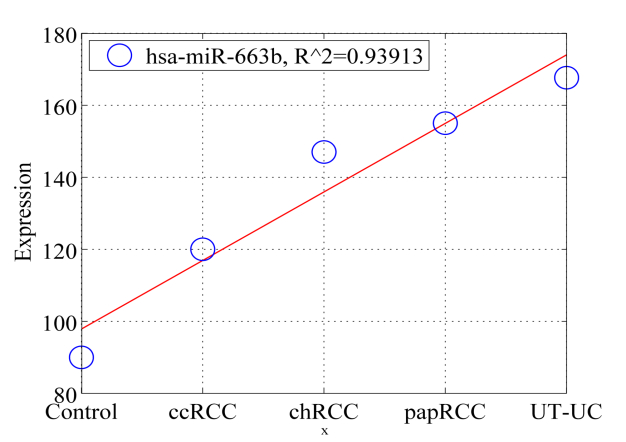

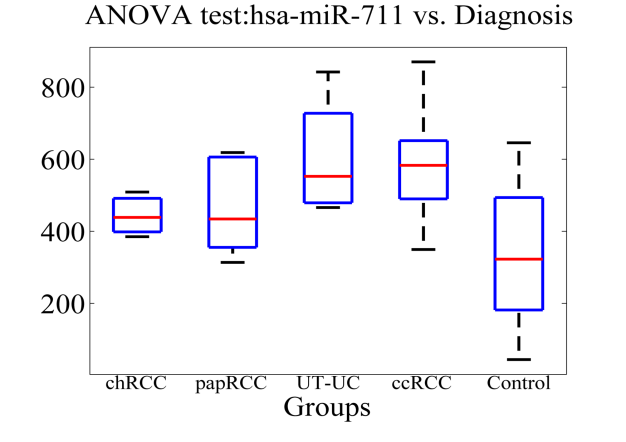

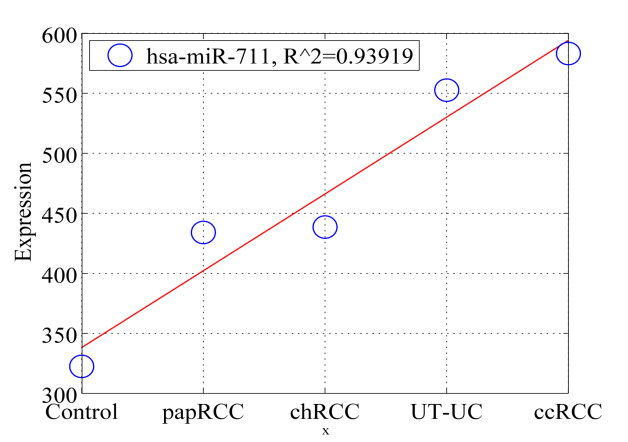


**B)**


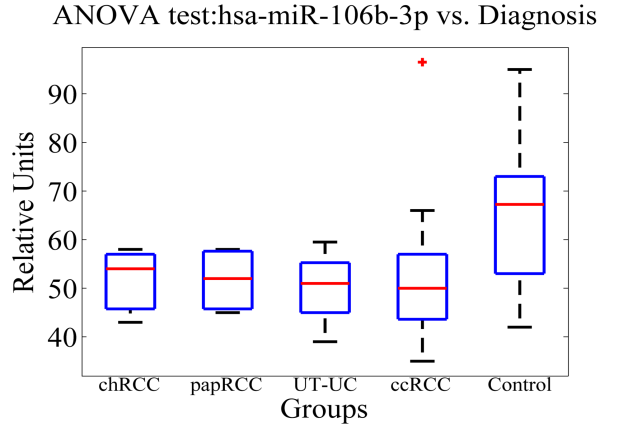

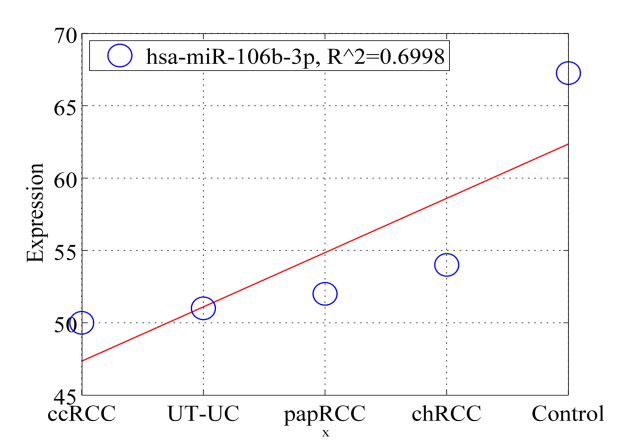

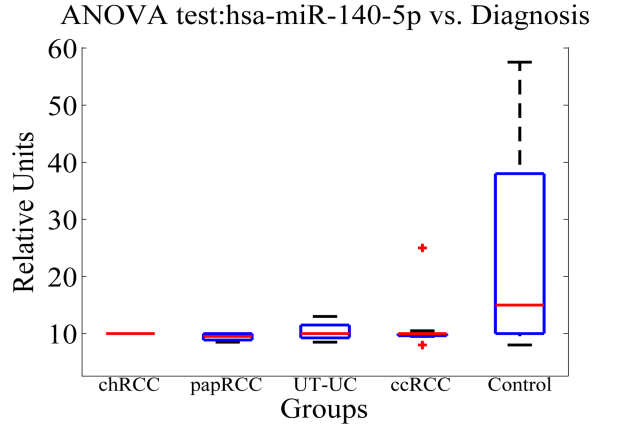

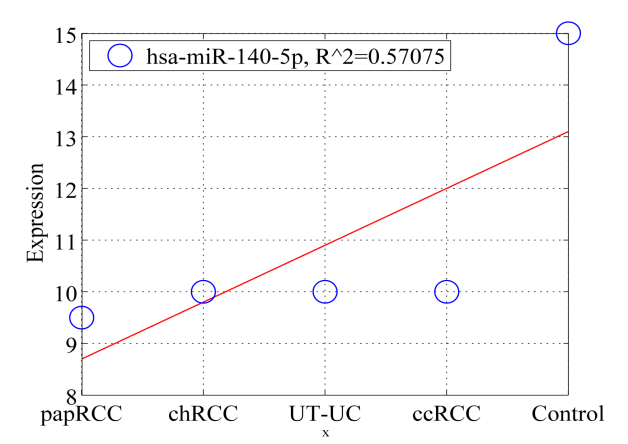

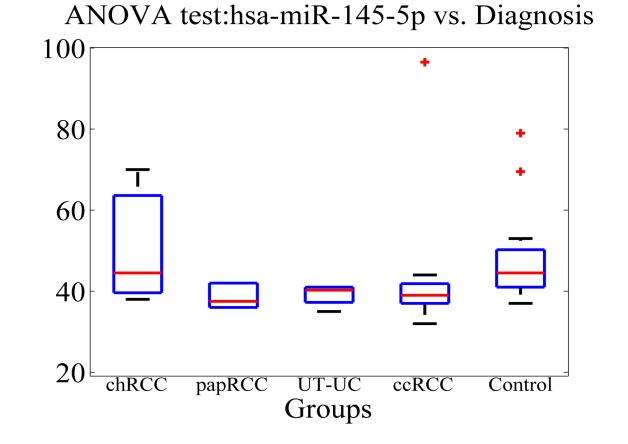

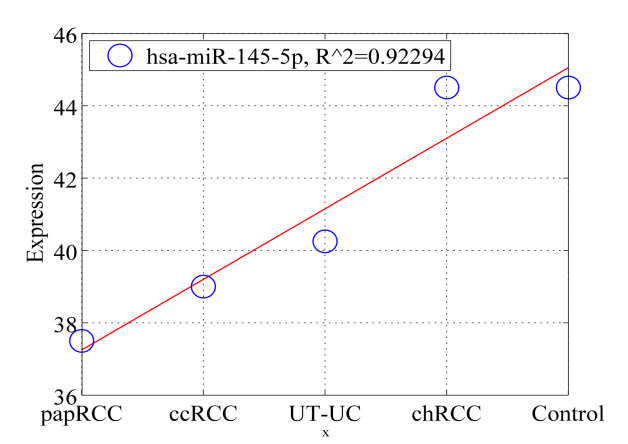

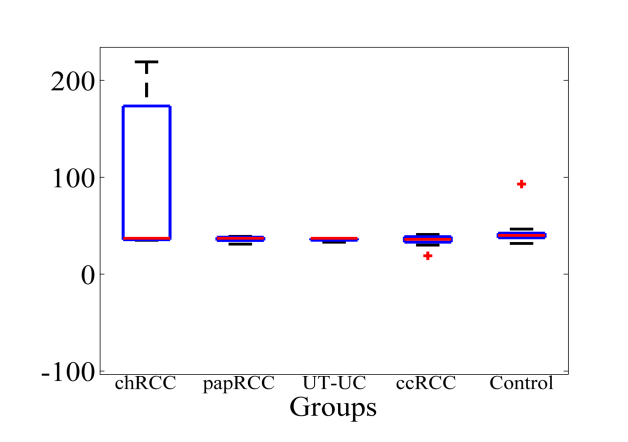

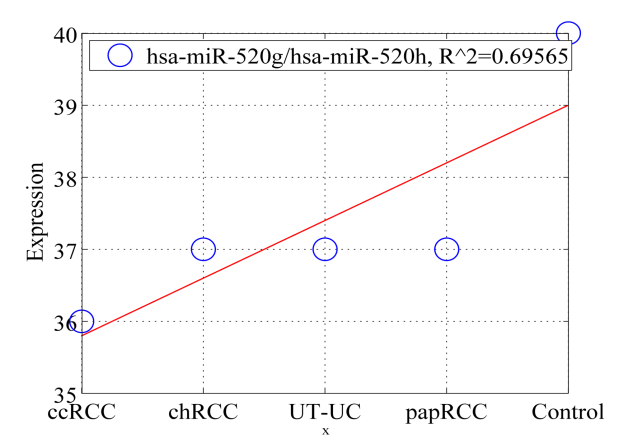

Supplement: Figure S2 — Regression analysis. Regression analysis of top up-regulated (A) and top down-regulated (B) miRNAs. miRNAs were tested with one-way ANOVA and further linear regression was used to fit their expression into a linear model. Left columns represent the ANOVA results. Right columns represent the linear regression results after sorting of the median values of the respective miRNAs in ascending order. (DOCX) [file pone.0091646.s002.docx]

**A)**


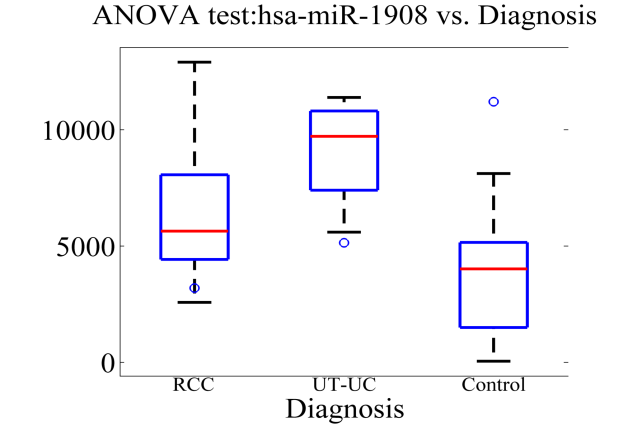

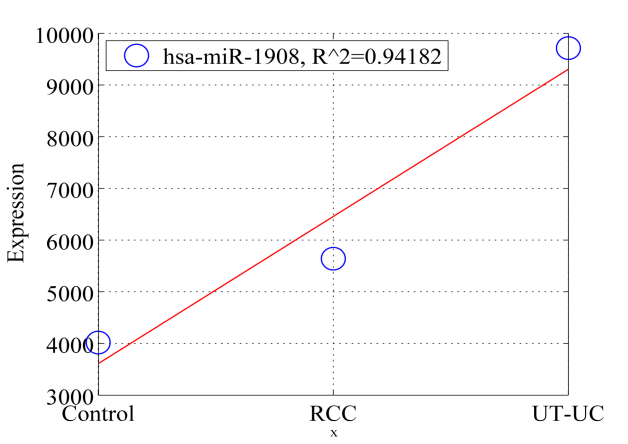

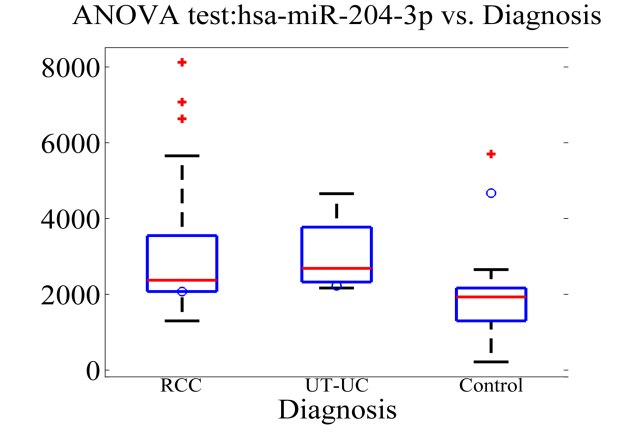

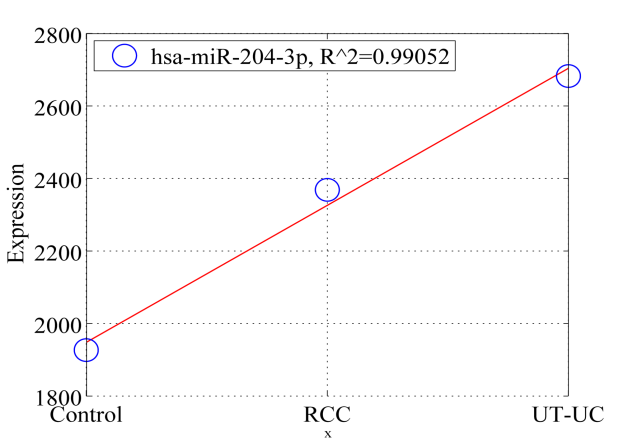

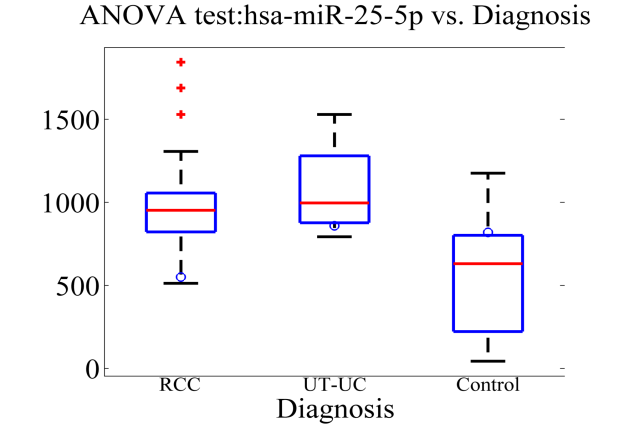

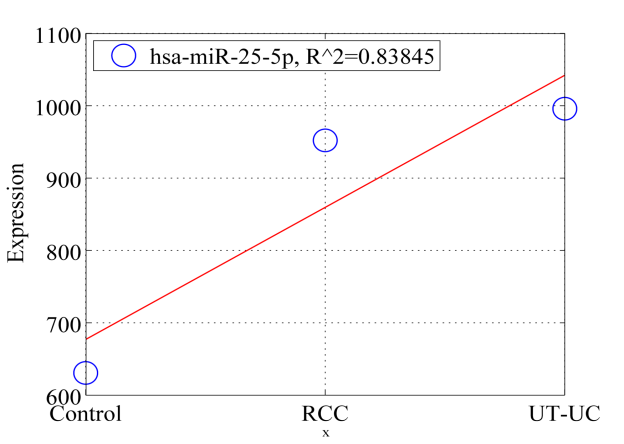

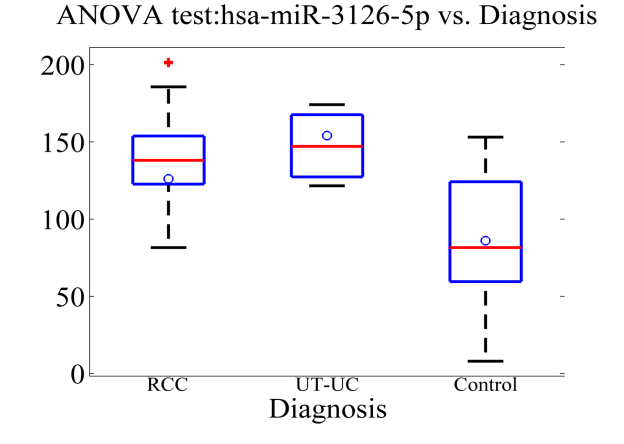

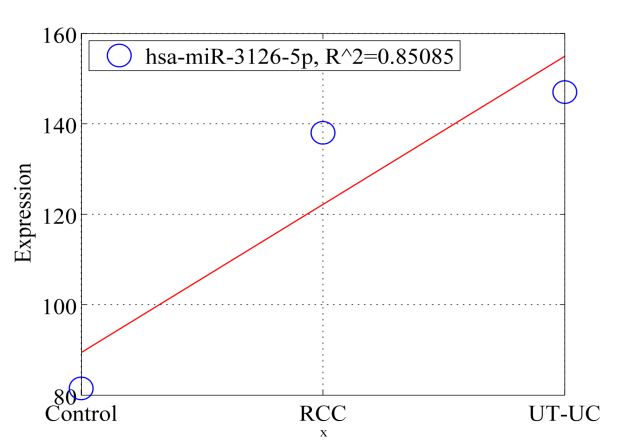

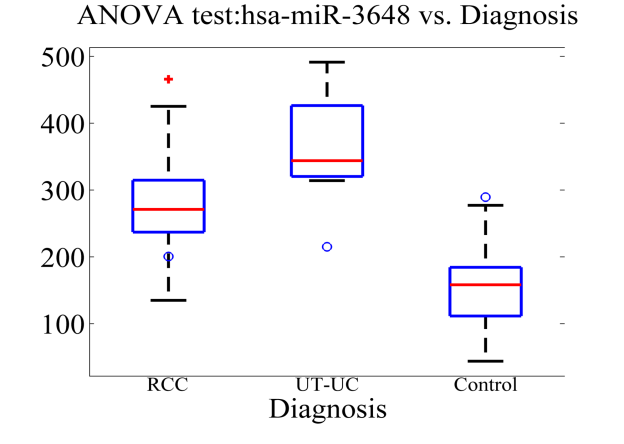

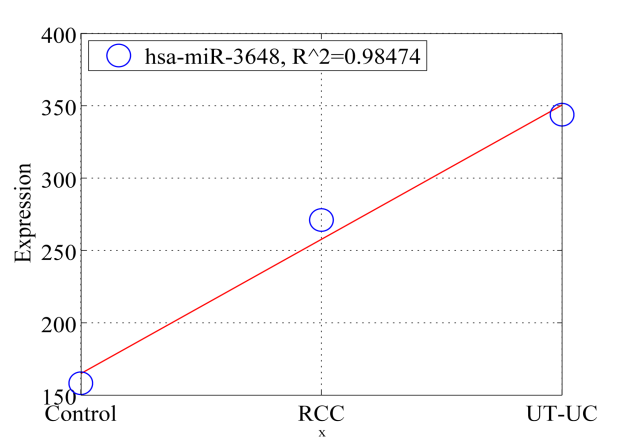

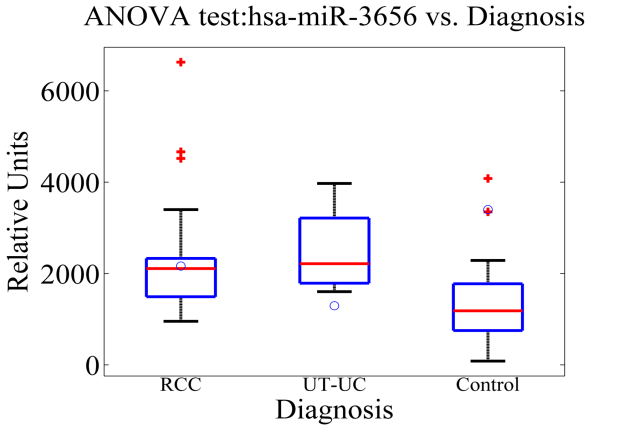

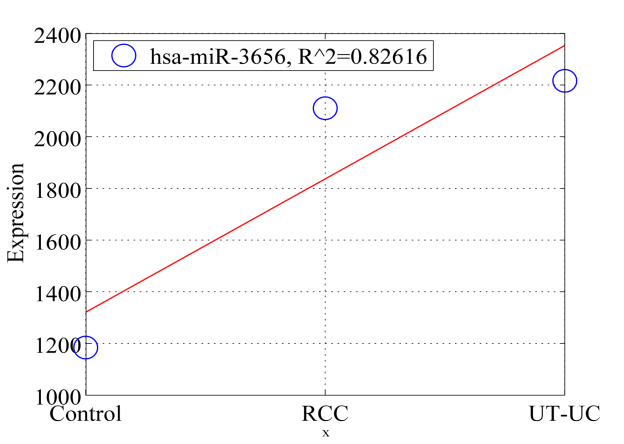

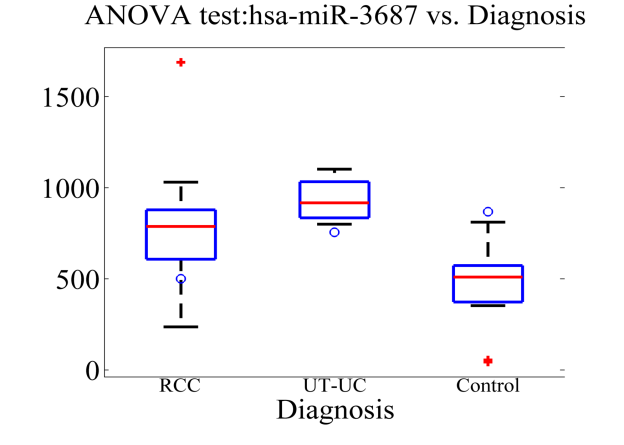

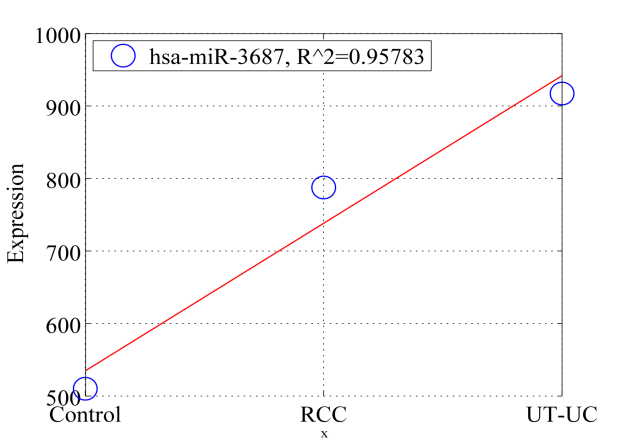

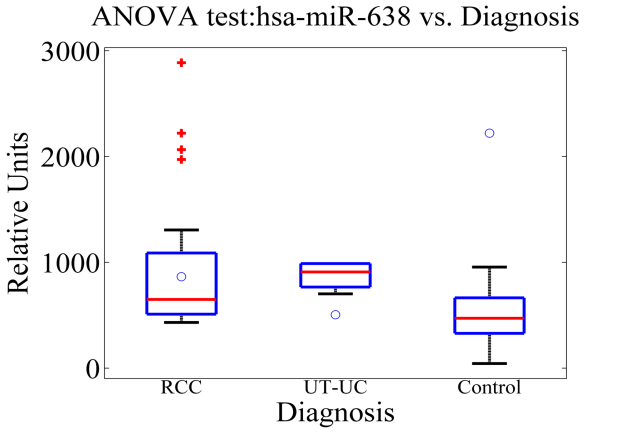

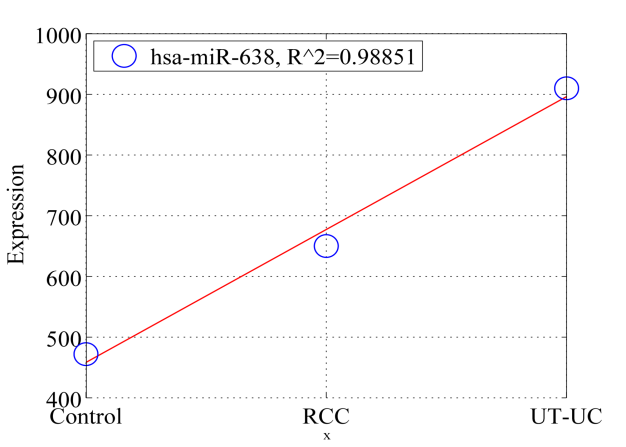

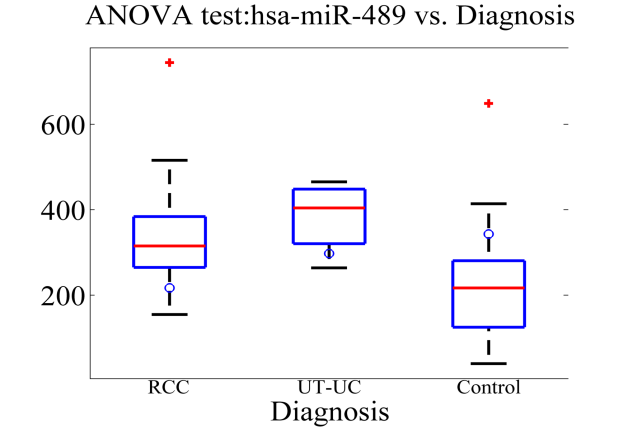

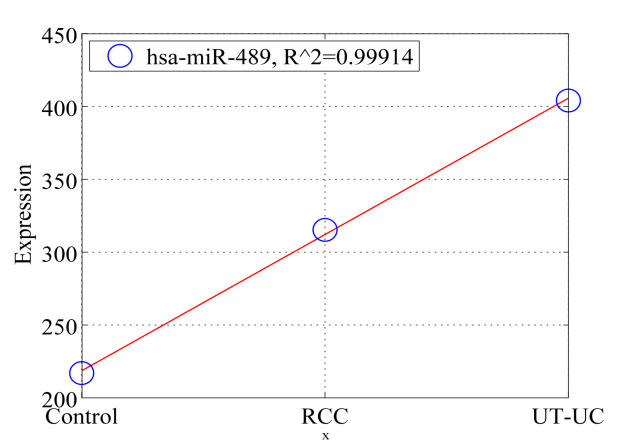

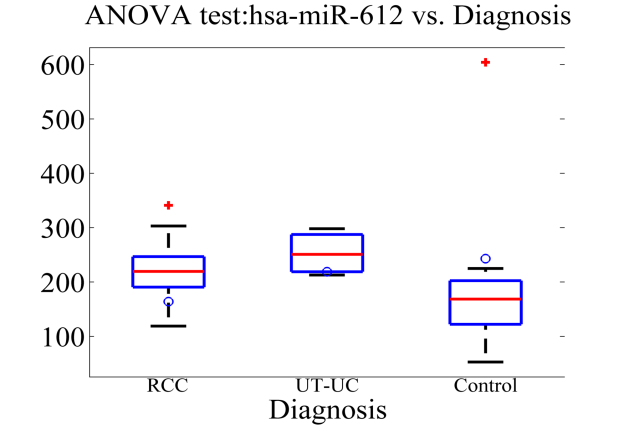

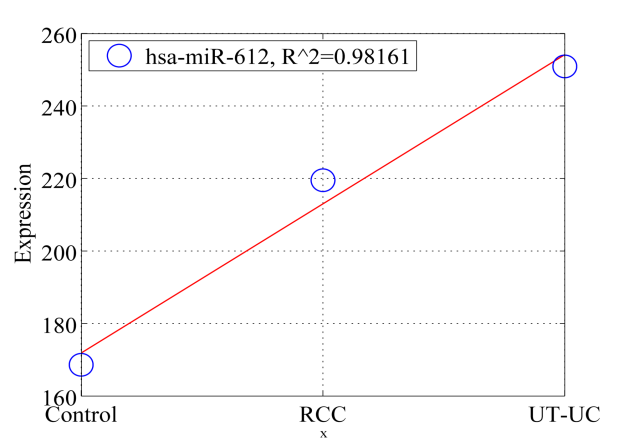

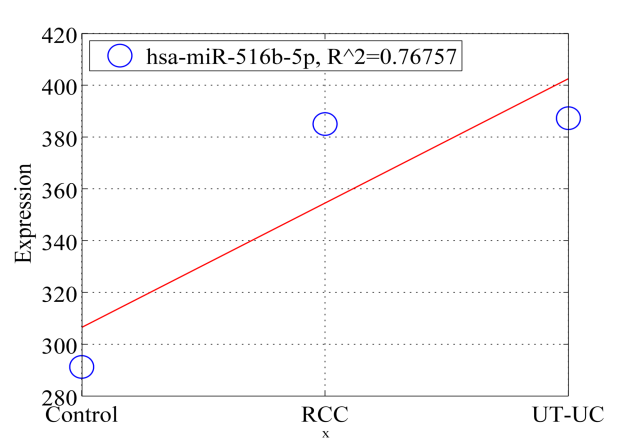


**M**


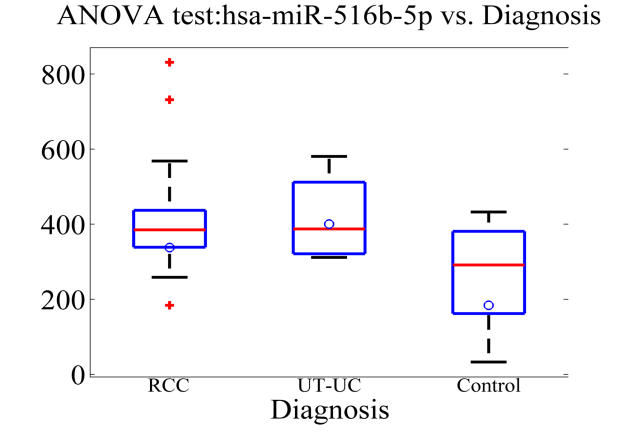


**M**


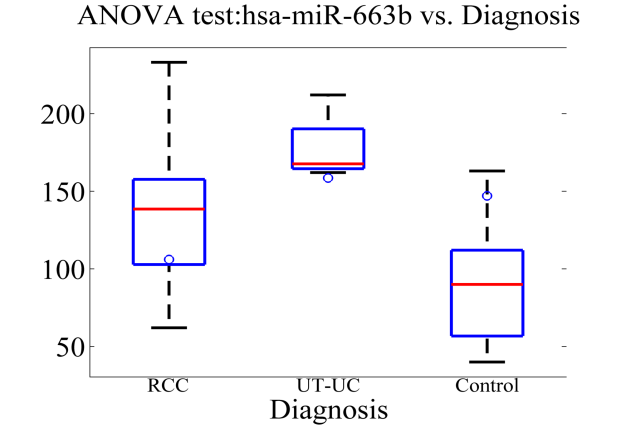

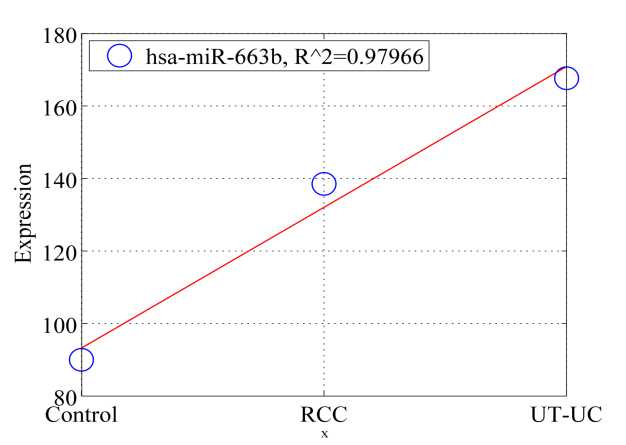

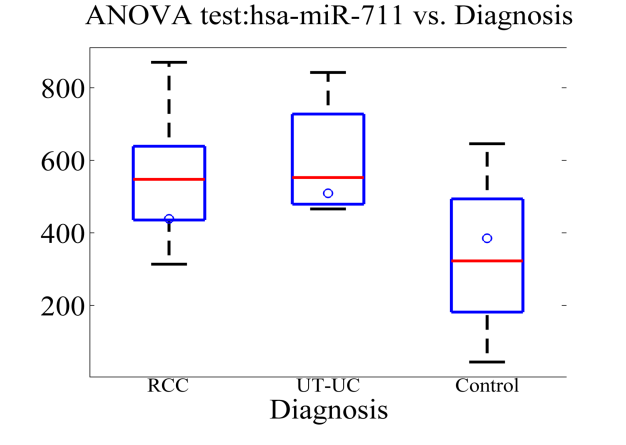

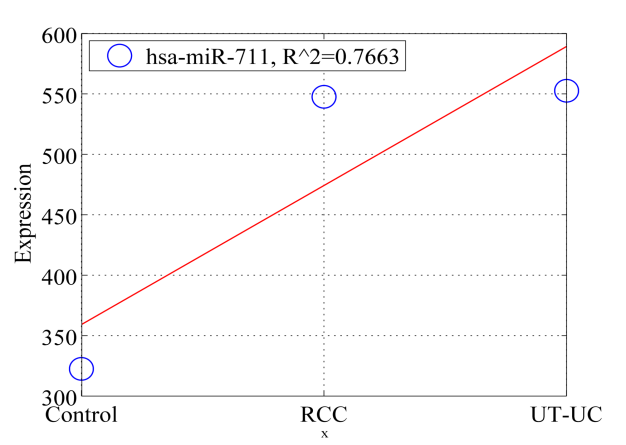

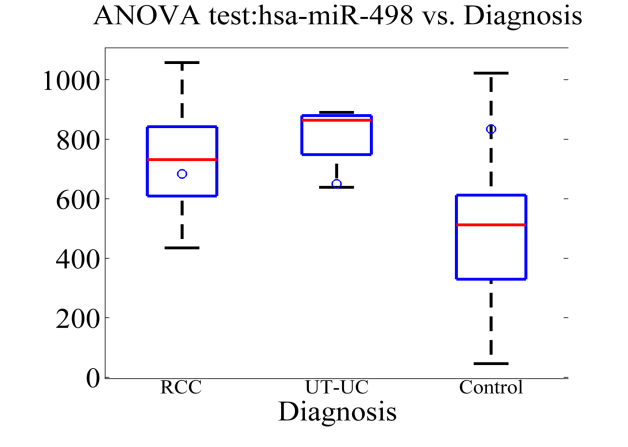

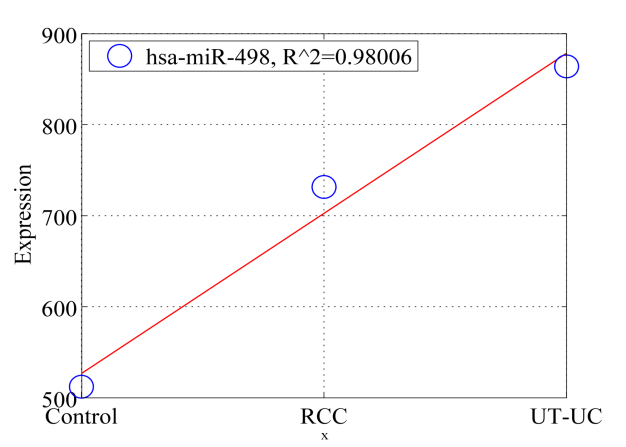


**B)**


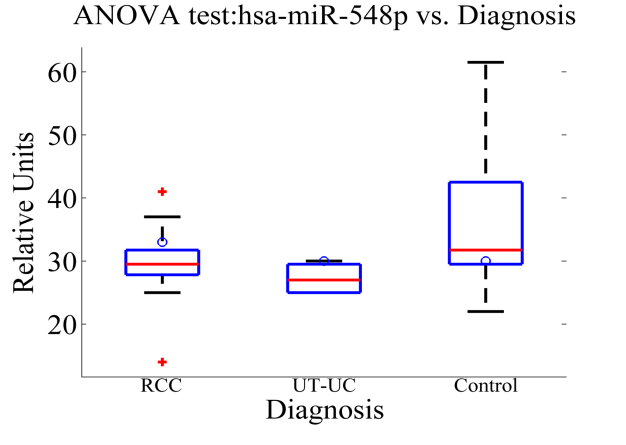

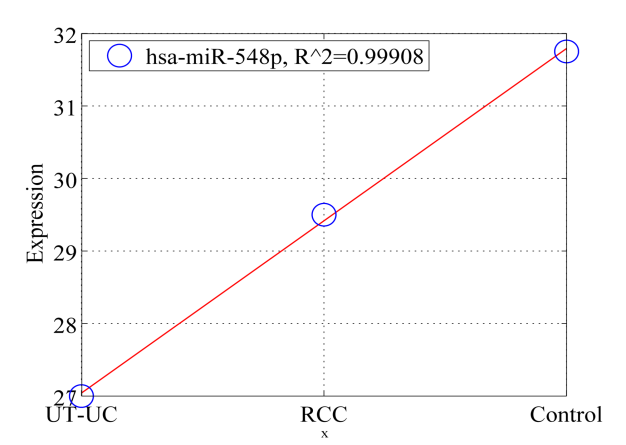

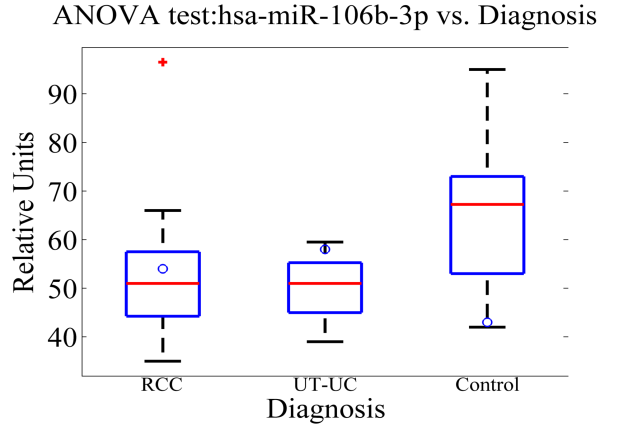

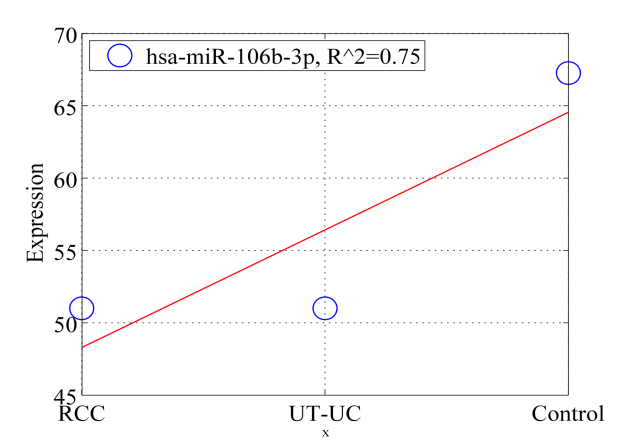

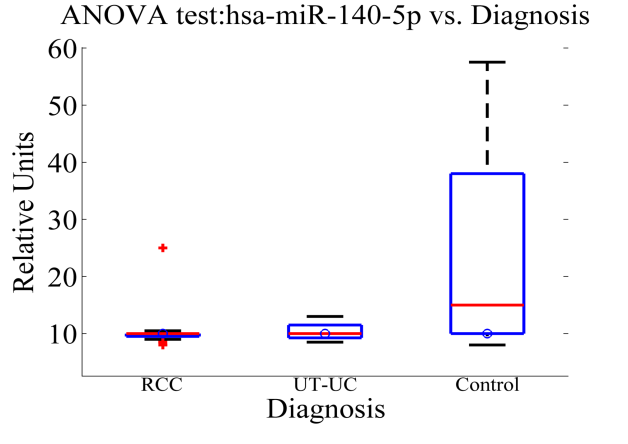

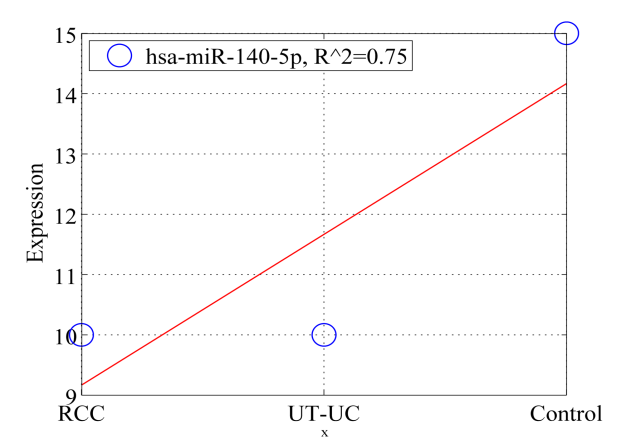

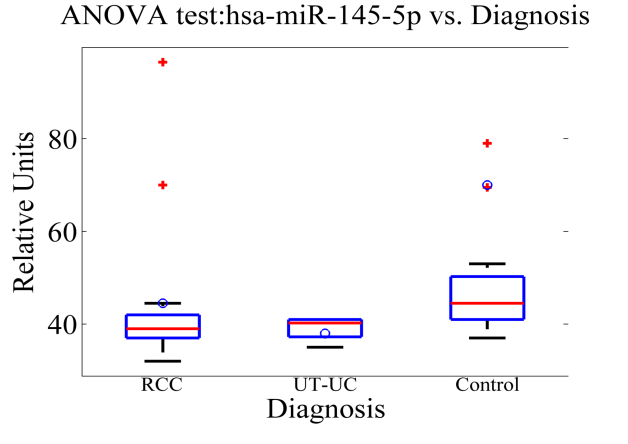

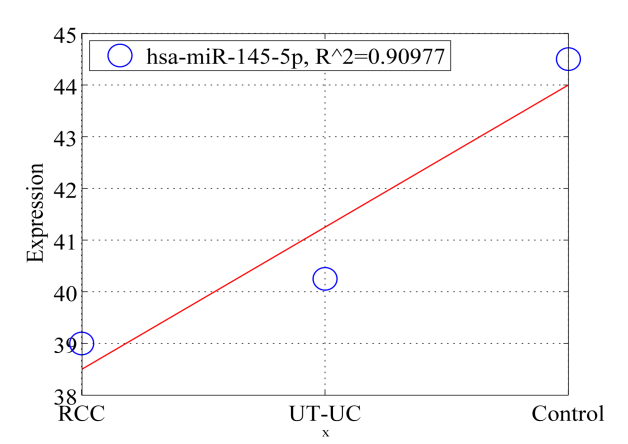

Supplement: Figure S3 — Regression analysis. Regression analysis of top up-regulated (A) and top down-regulated (B) miRNAs. miRNAs were tested with one-way ANOVA and further linear regression was used to fit their expression into a linear model. Left columns represent the ANOVA results. Right columns represent the linear regression results after sorting of the median values of the respective miRNAs in ascending order. (DOCX) [file pone.0091646.s003.docx]

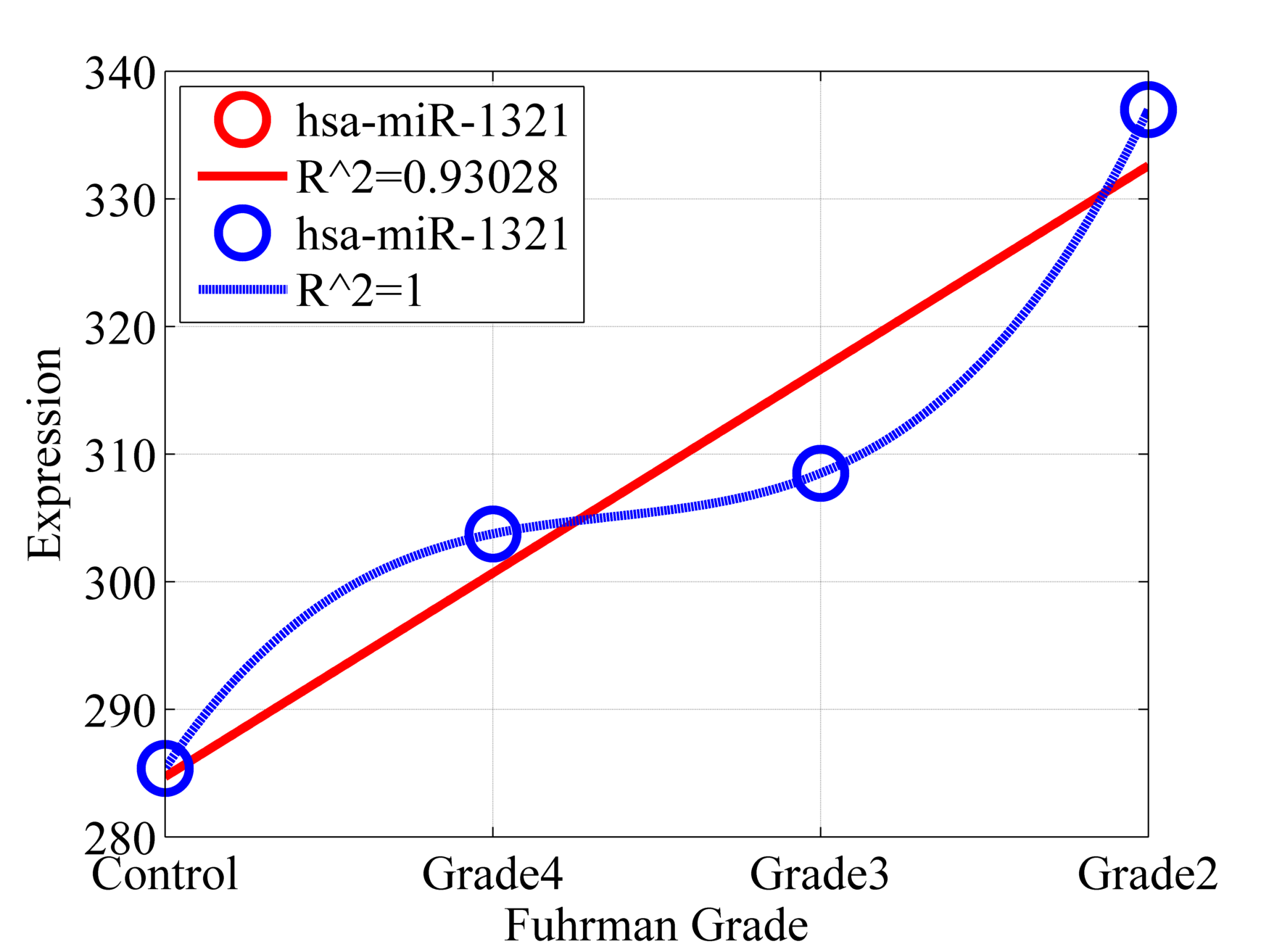

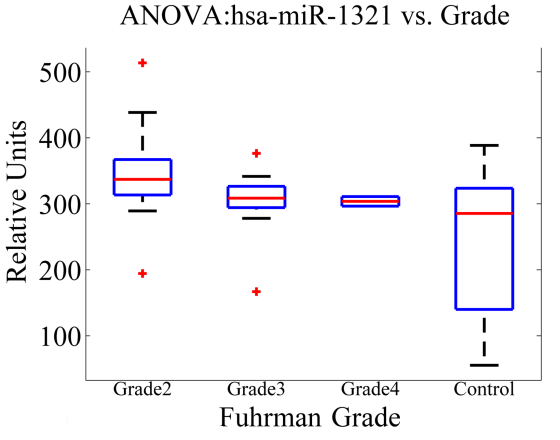


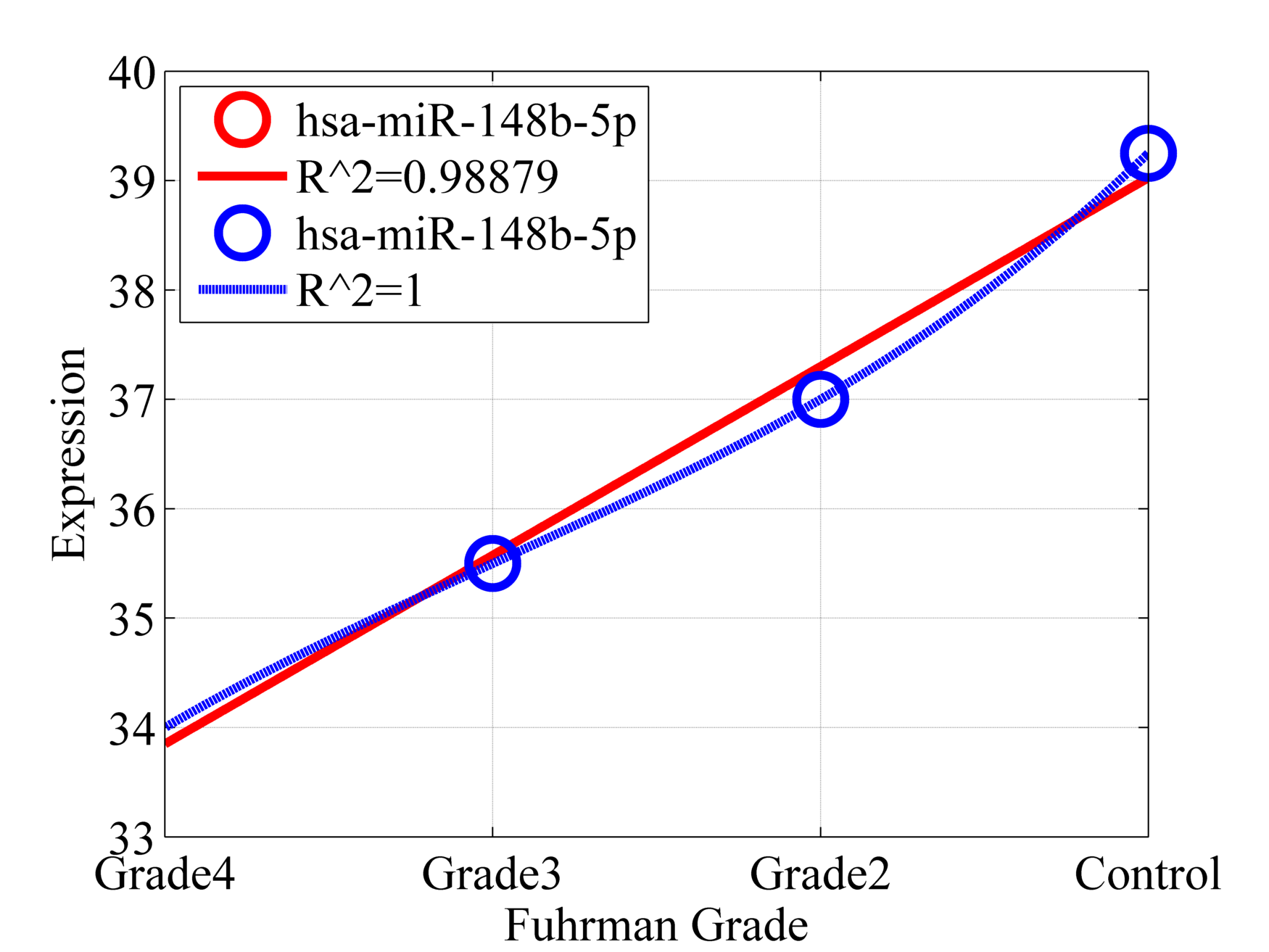

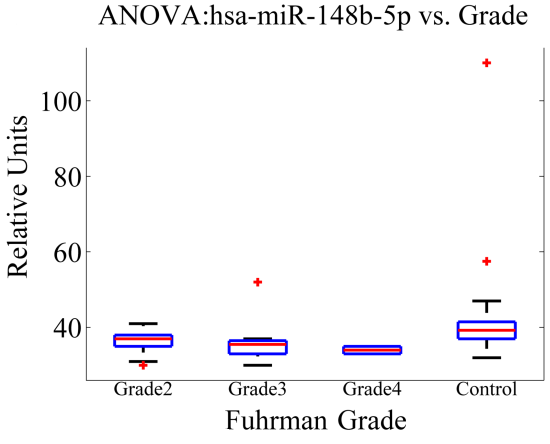


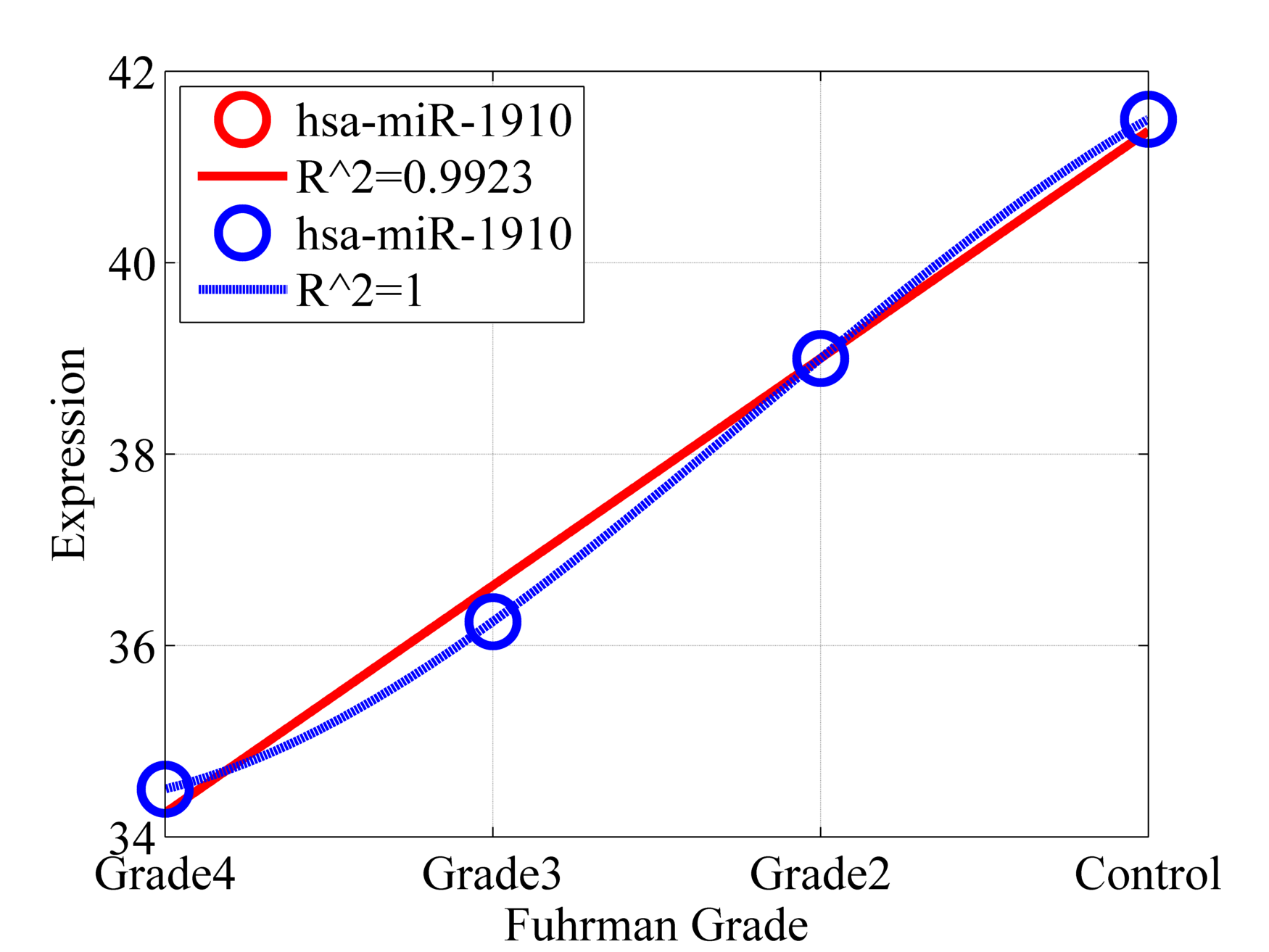


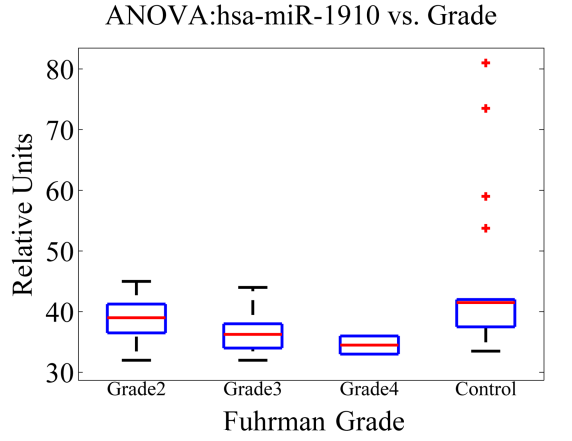


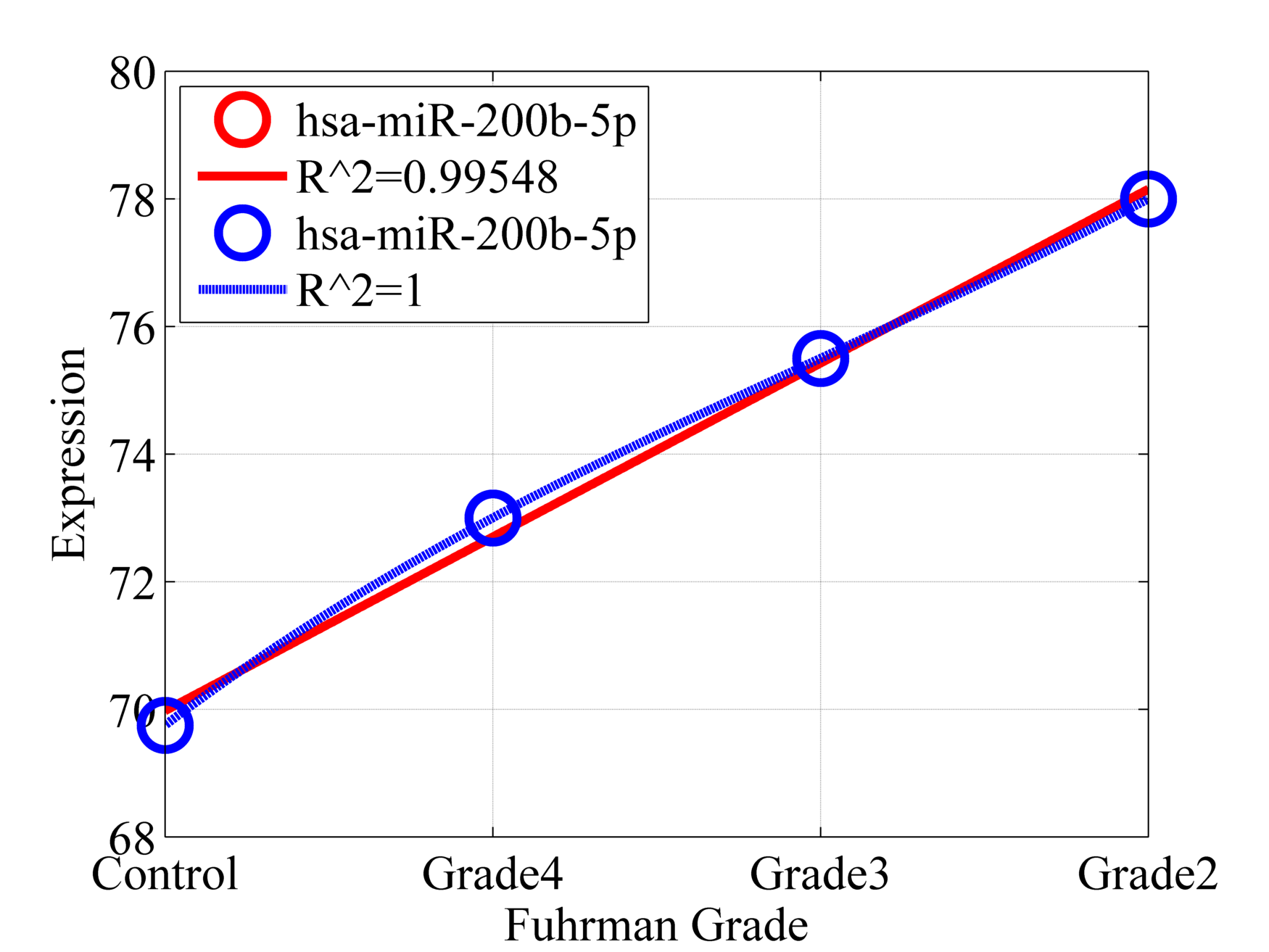


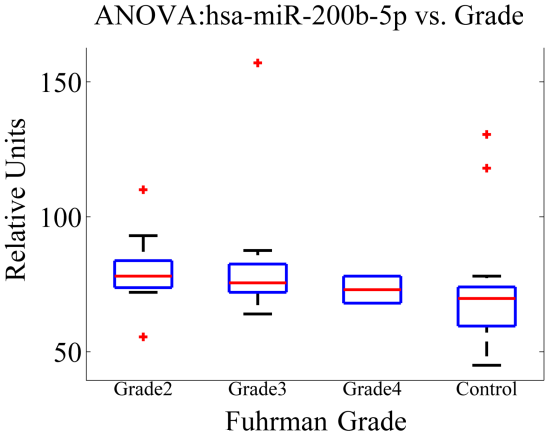


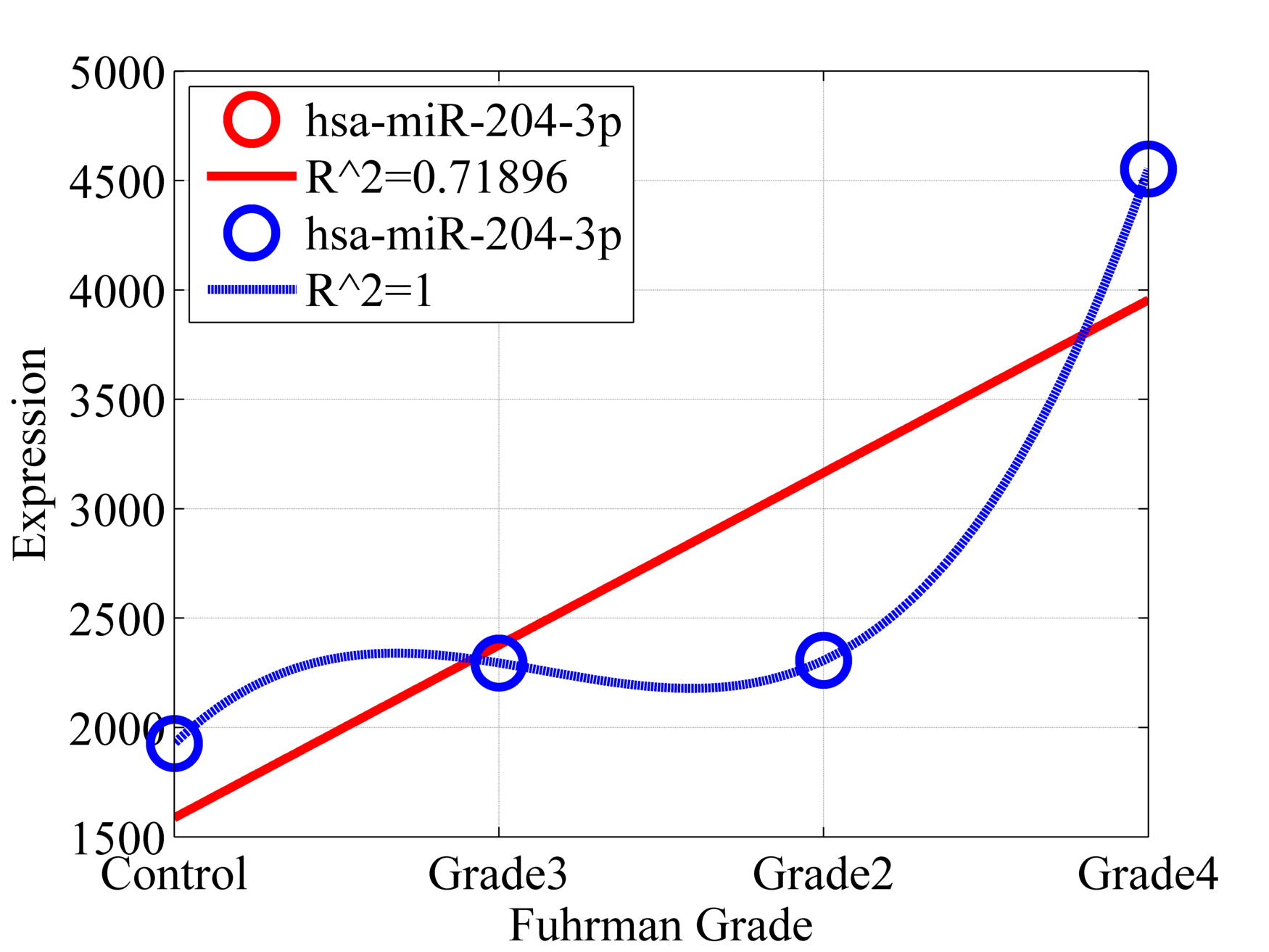

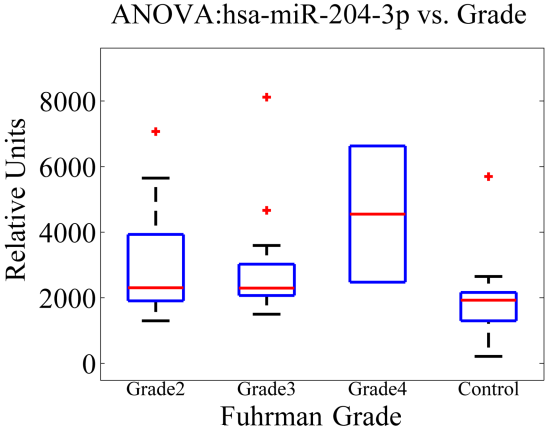


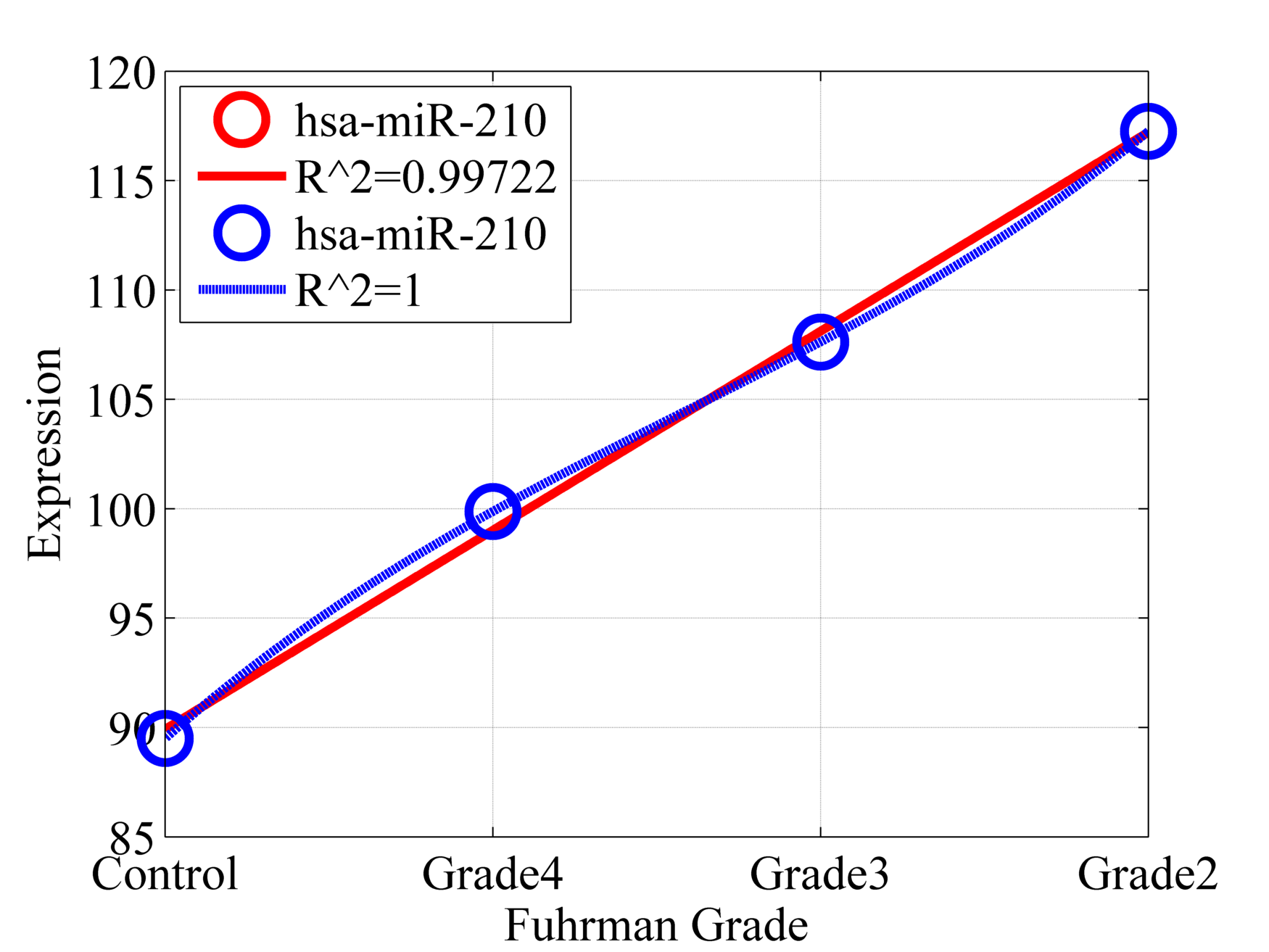


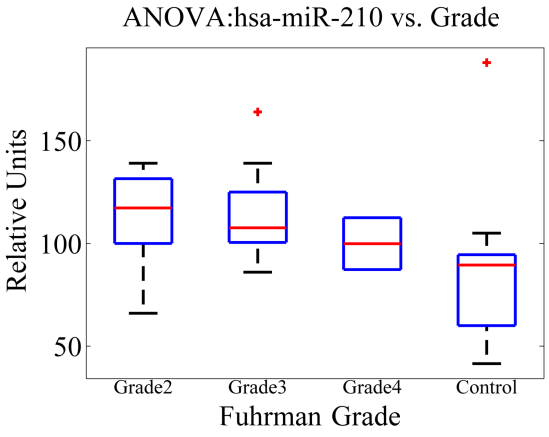


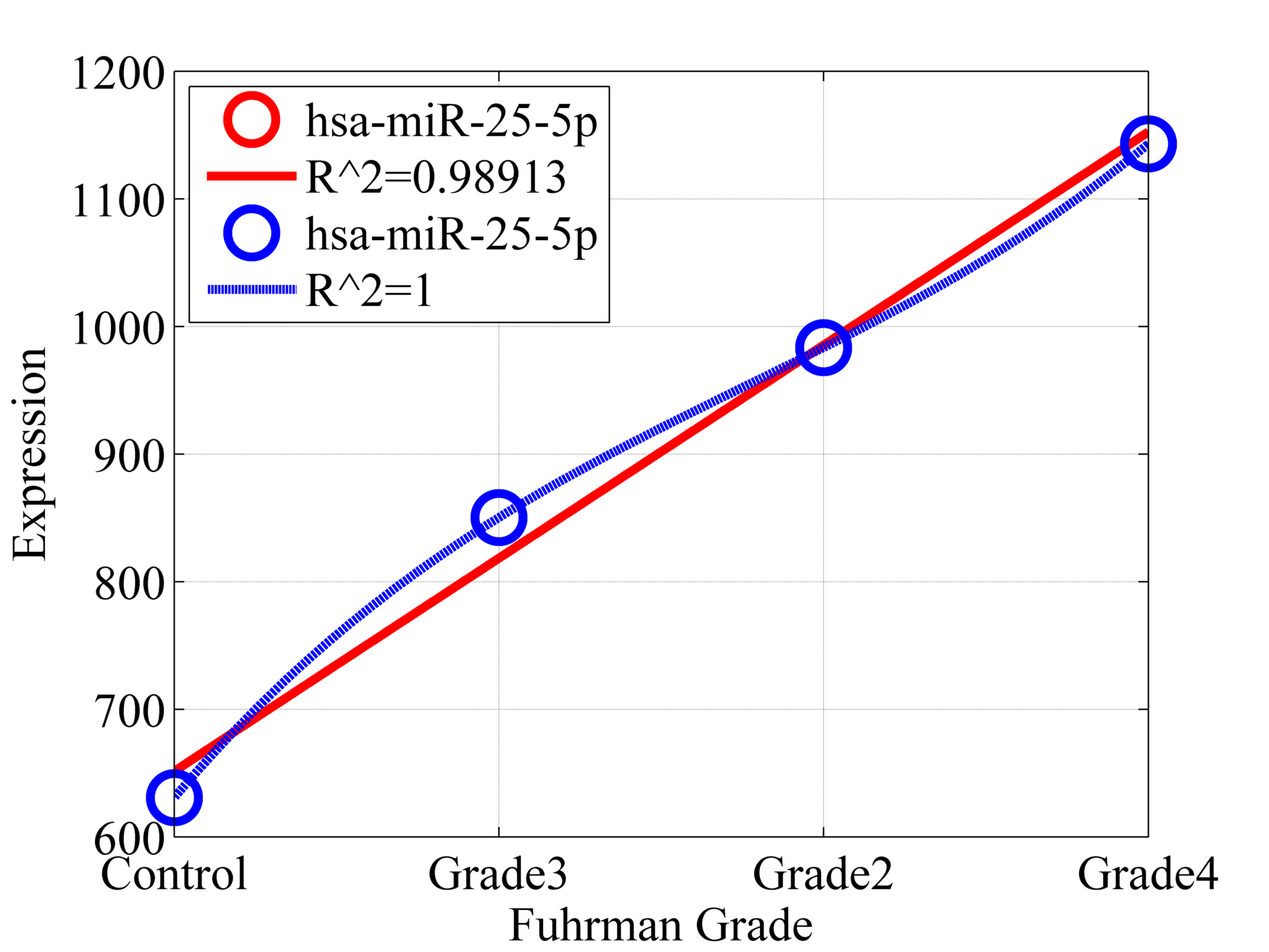


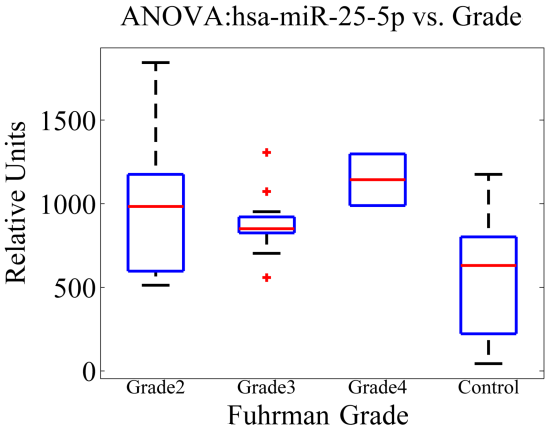


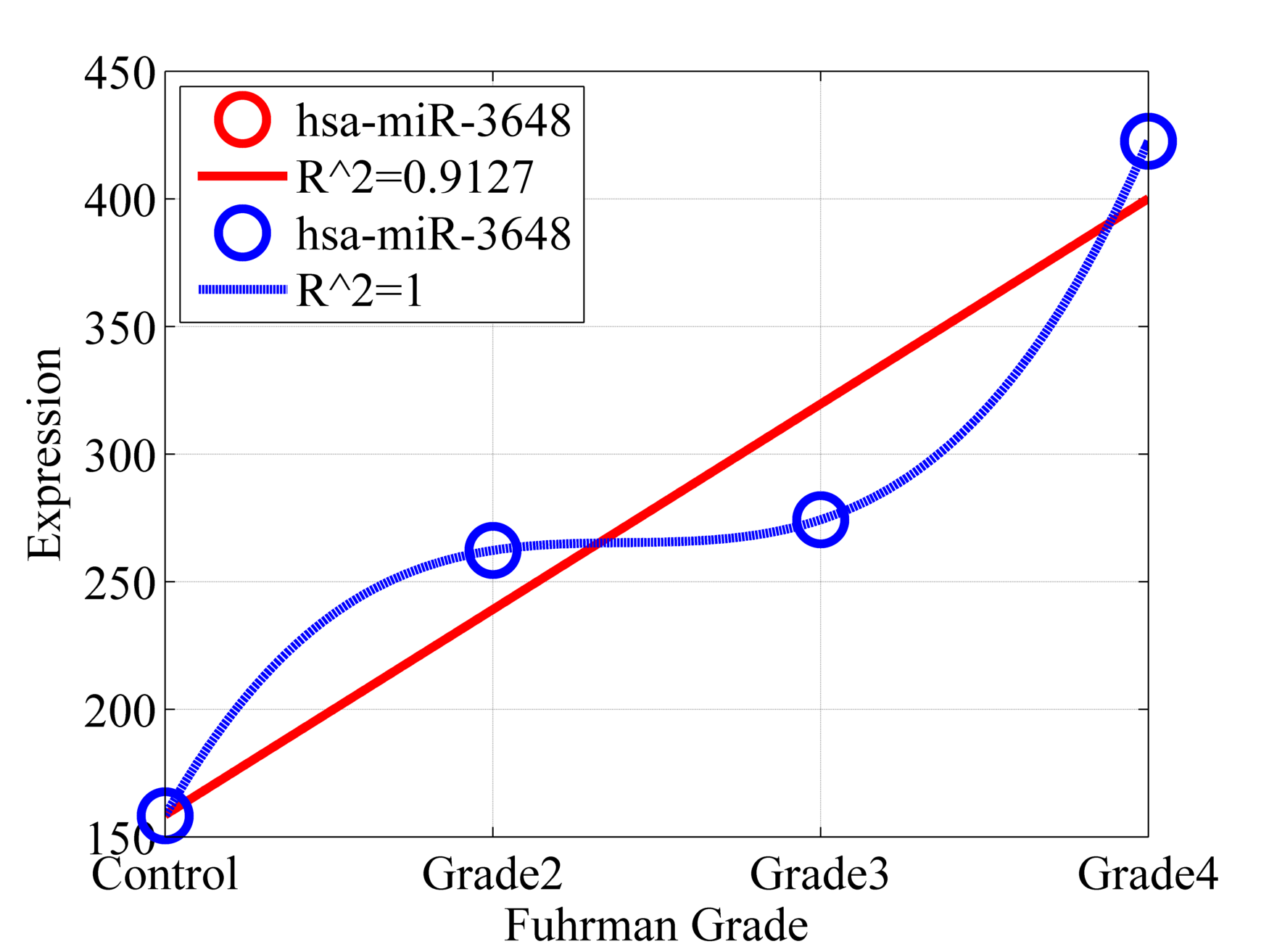


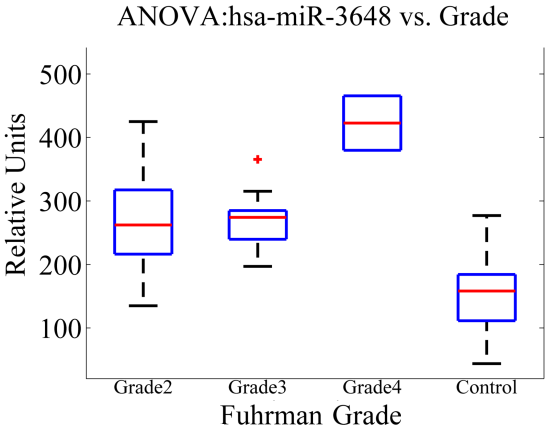


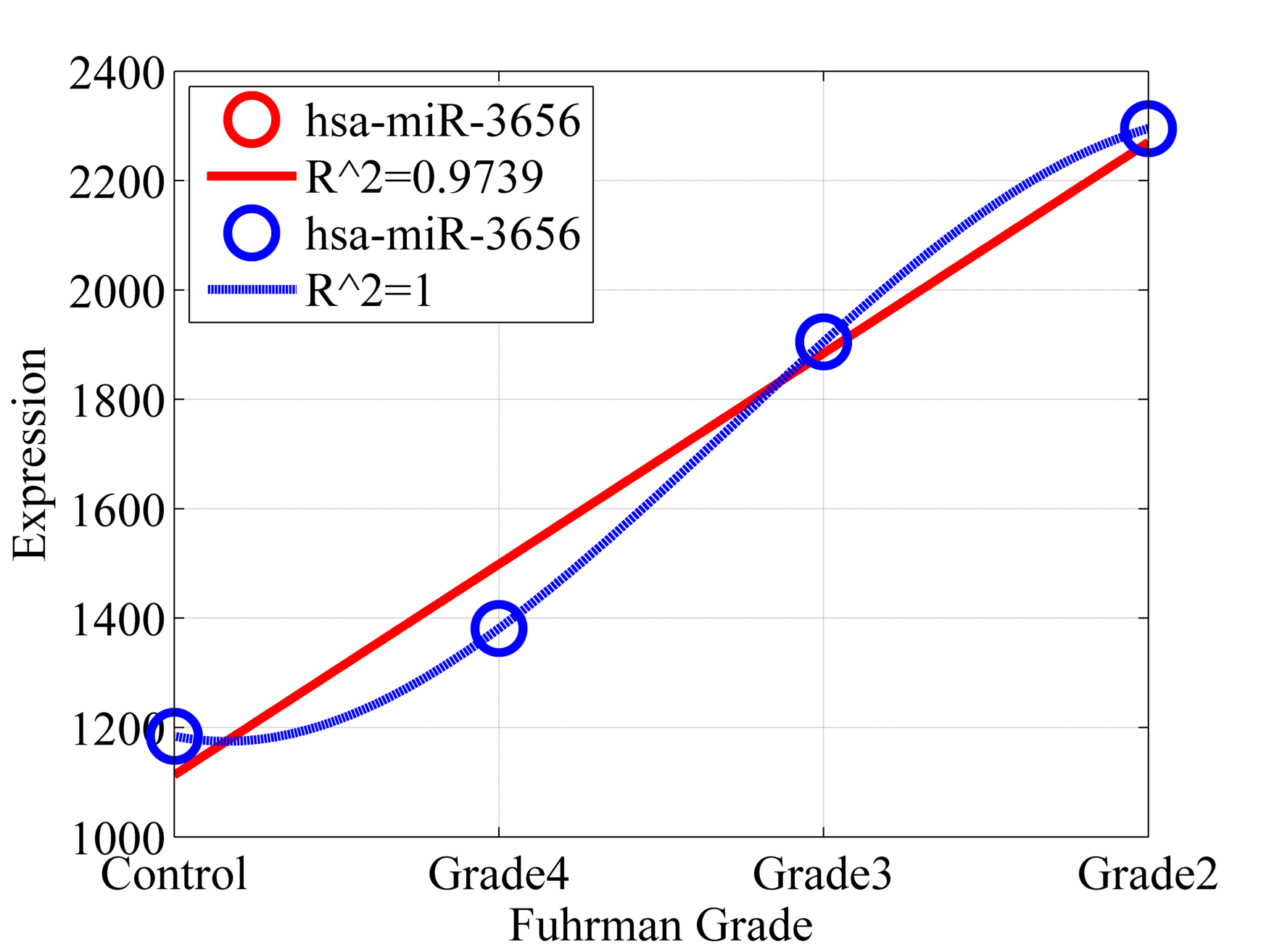

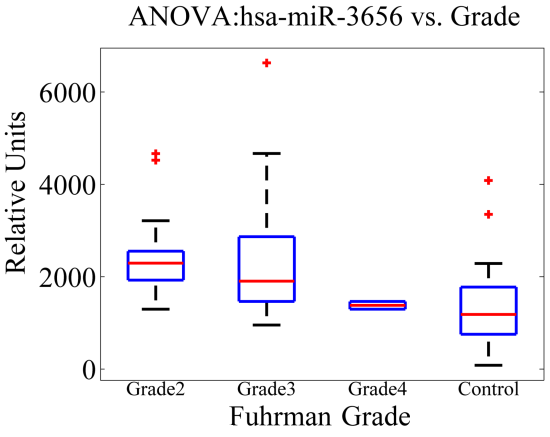


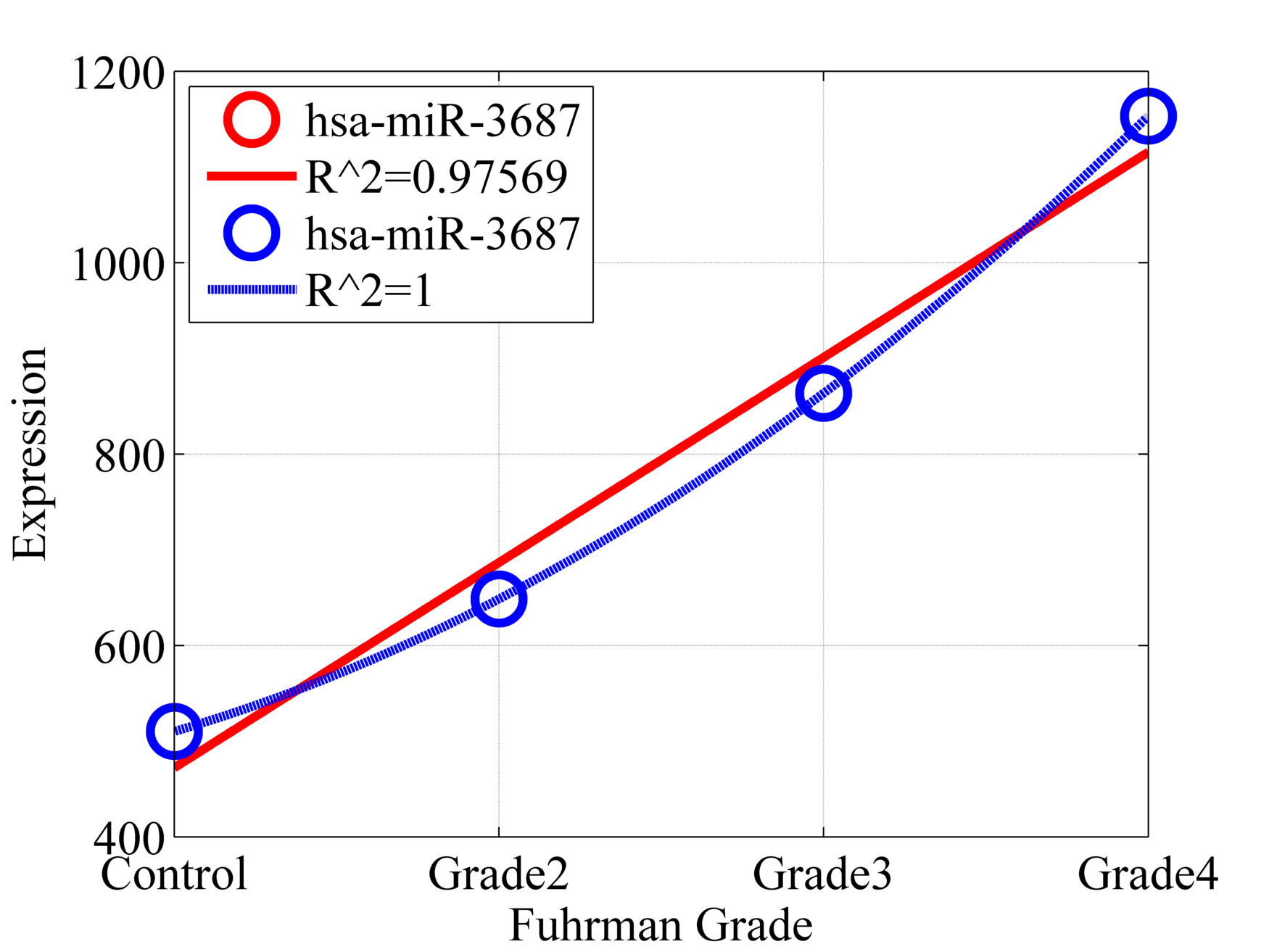

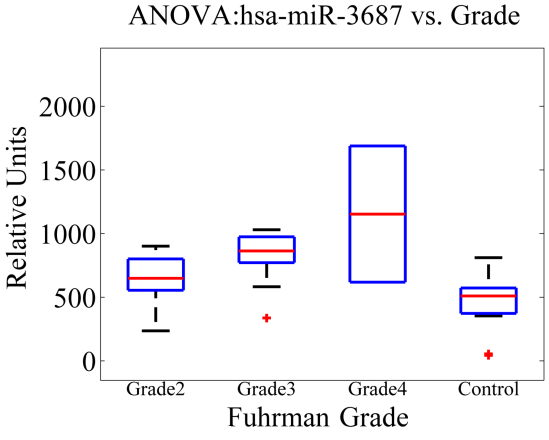


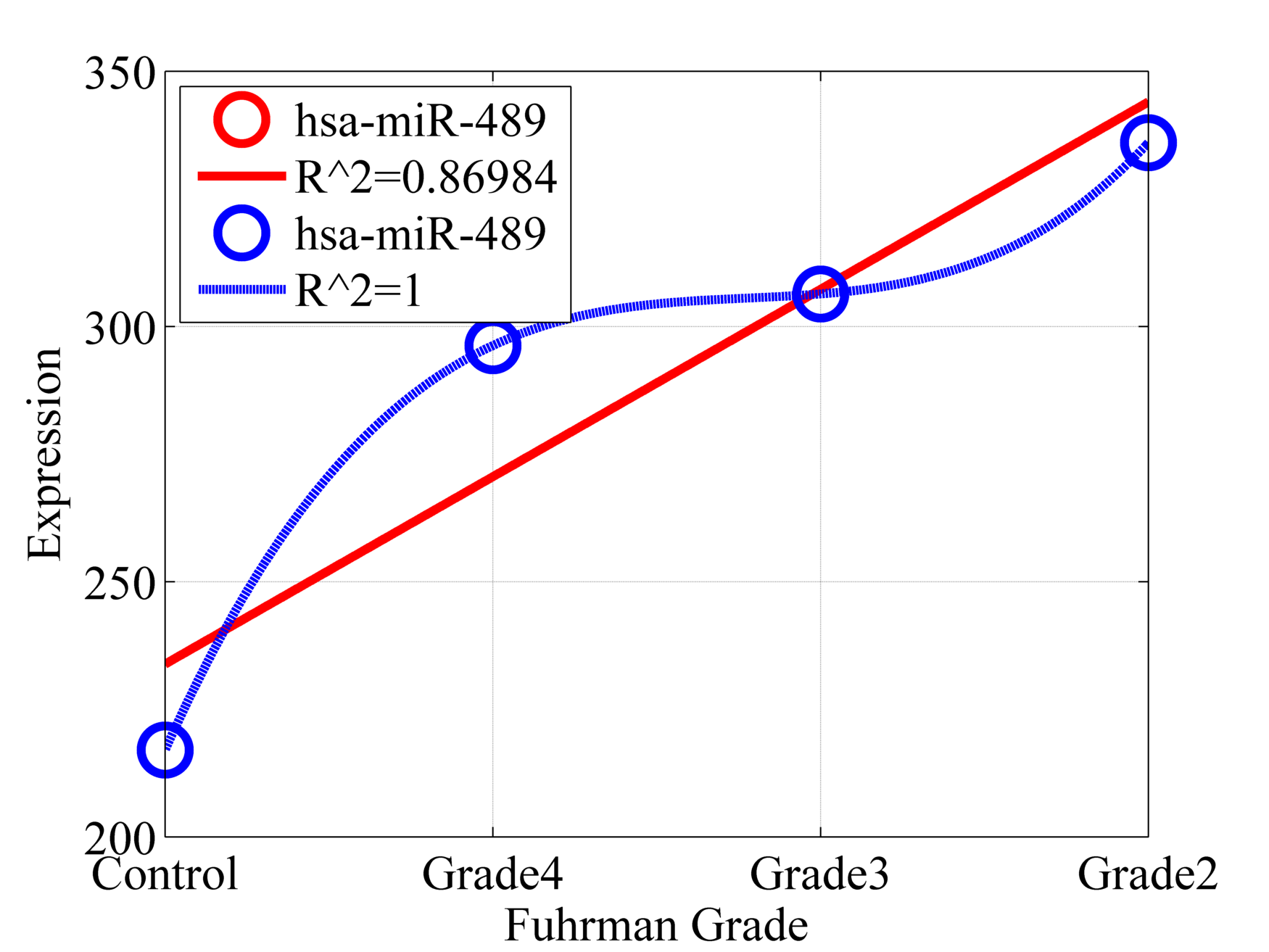

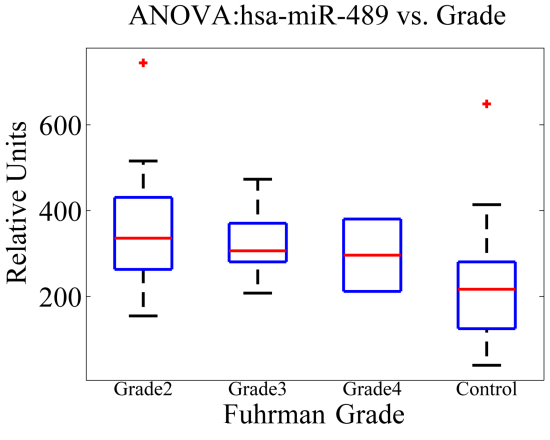


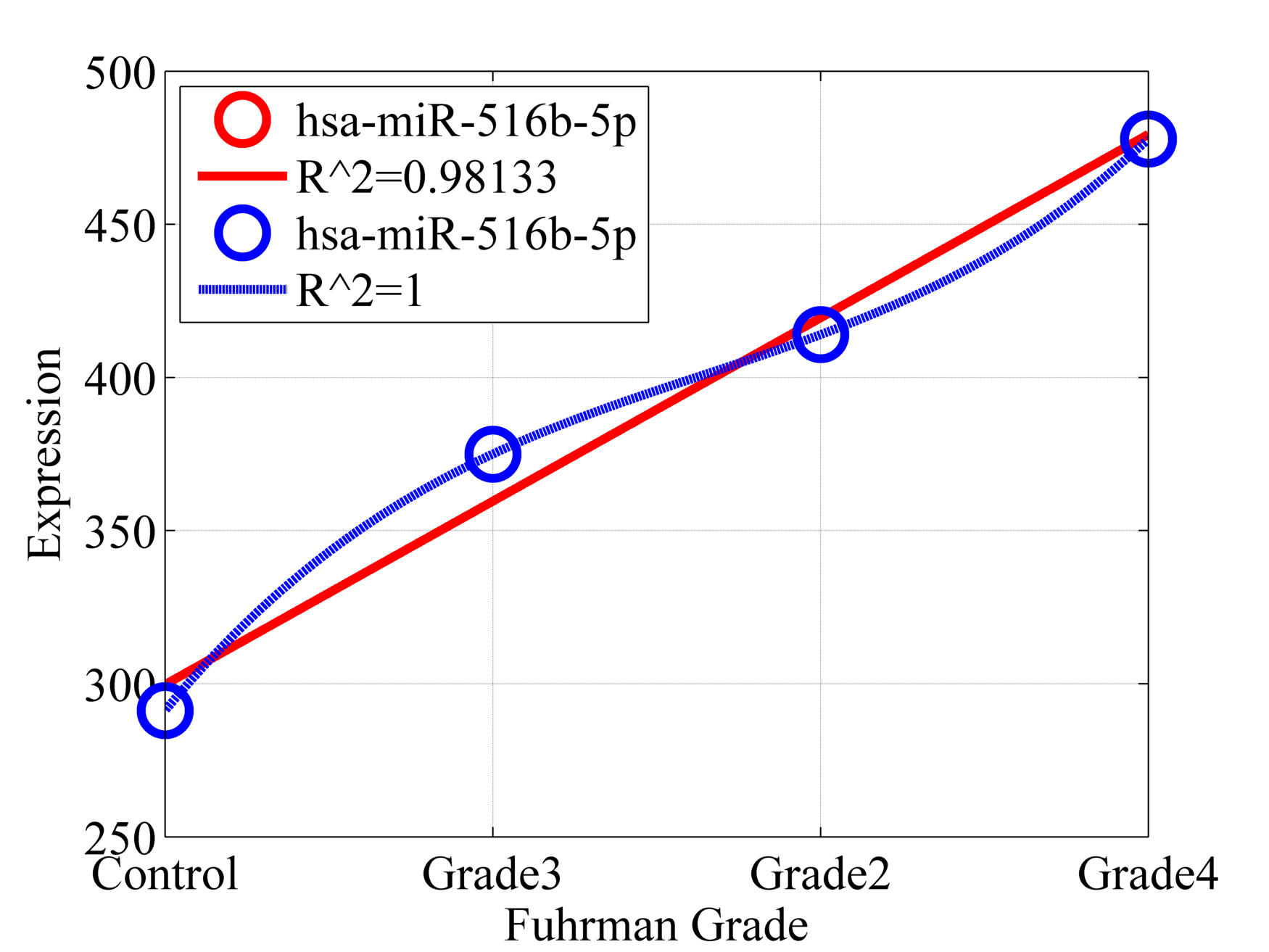

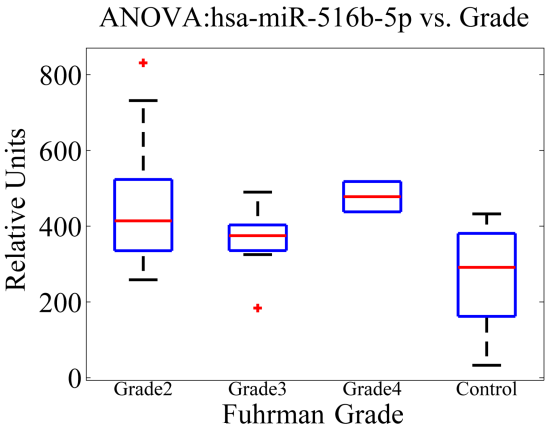


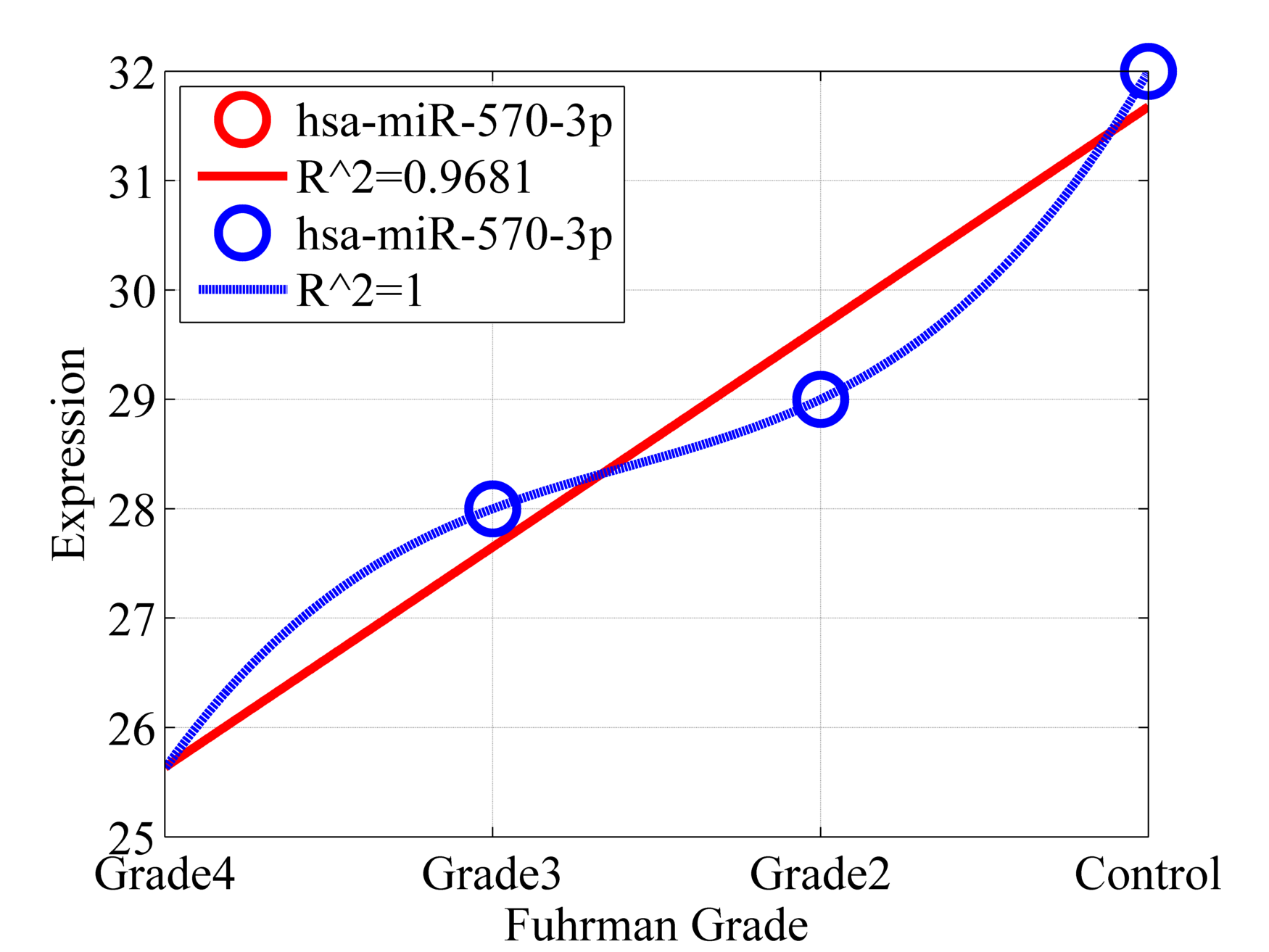

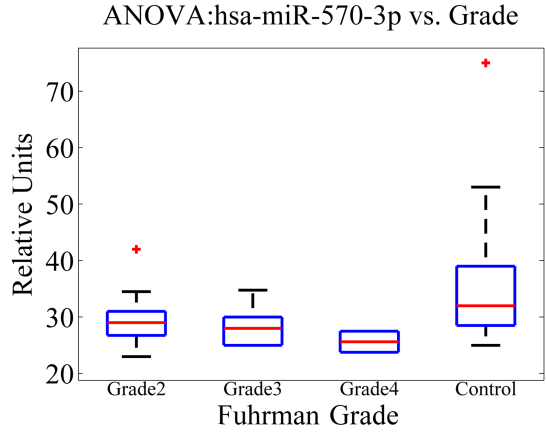


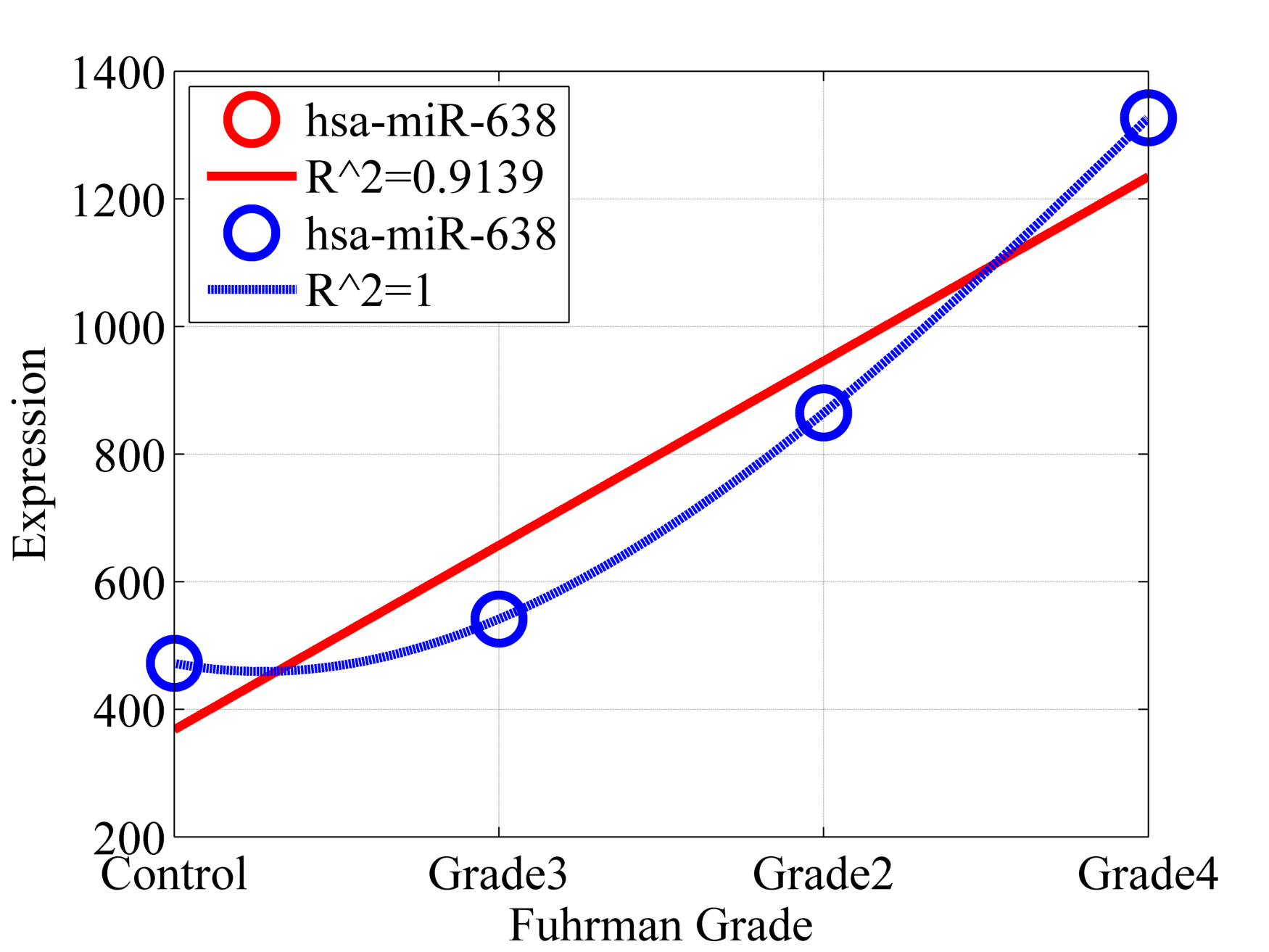

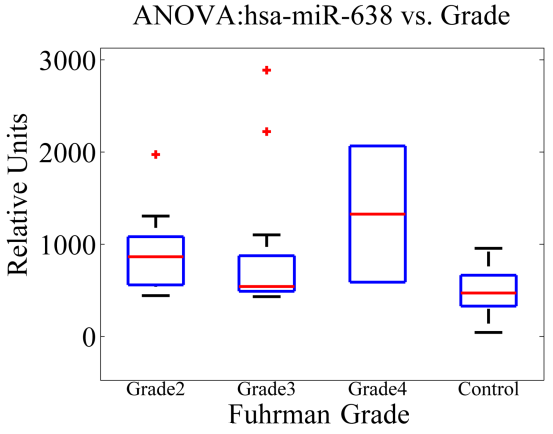


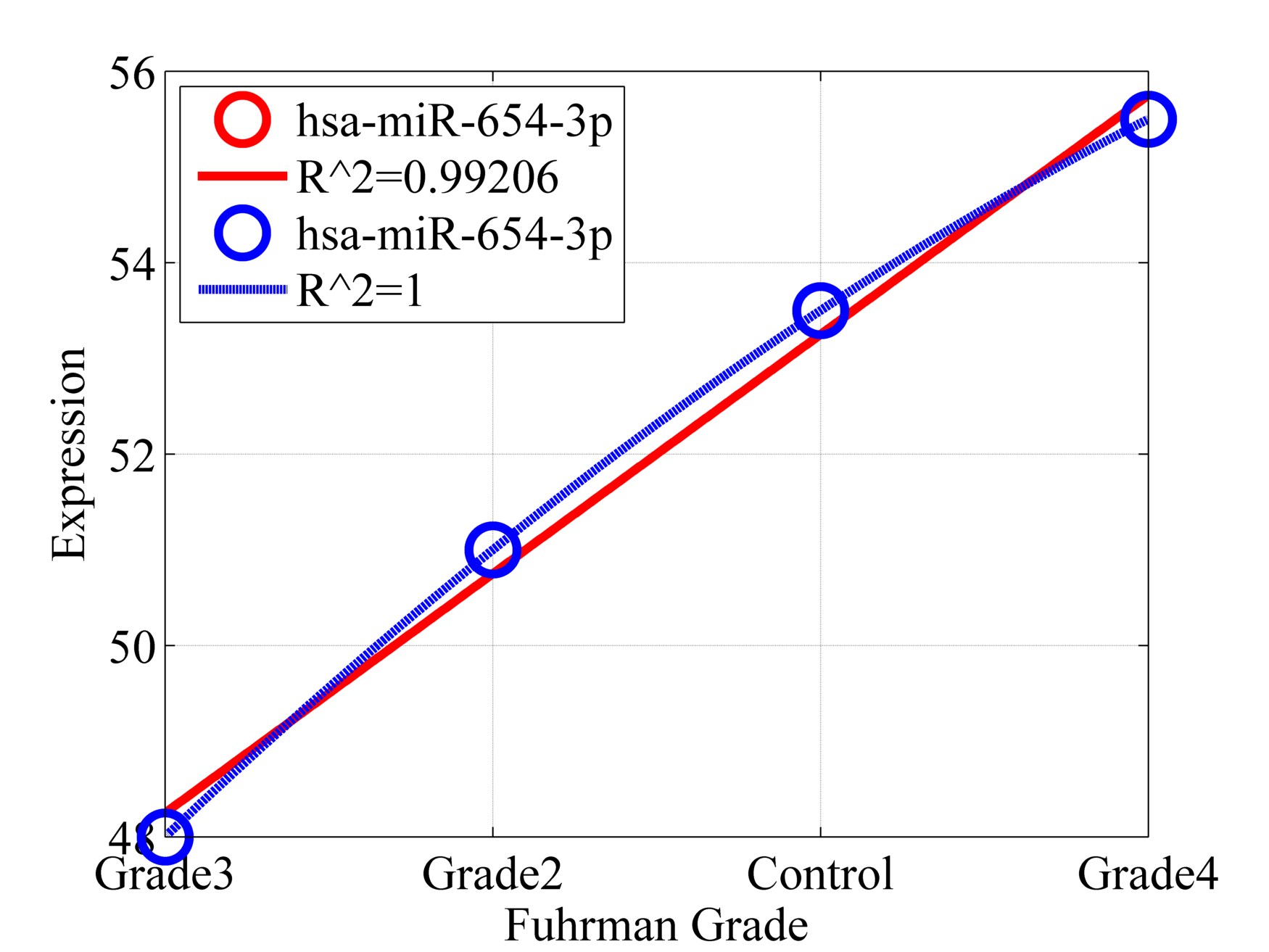

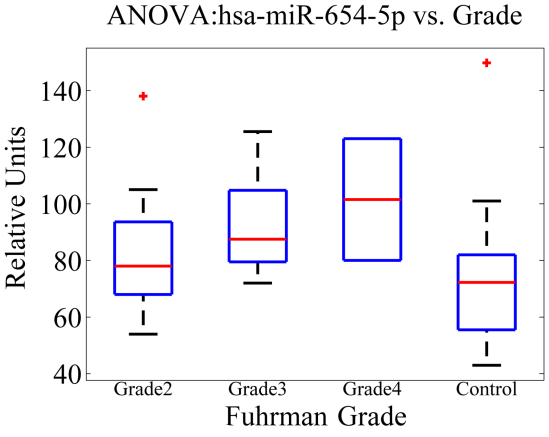


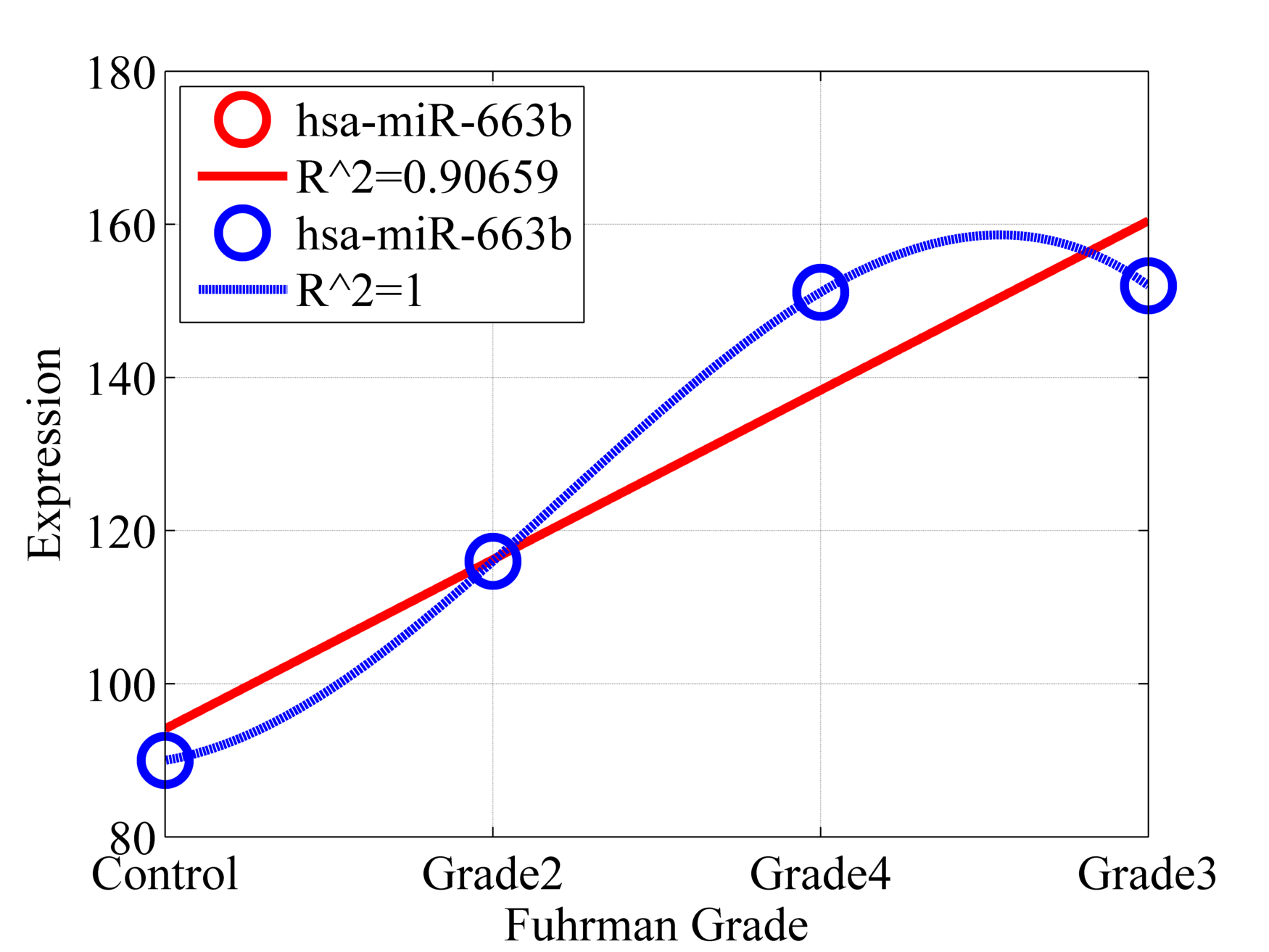

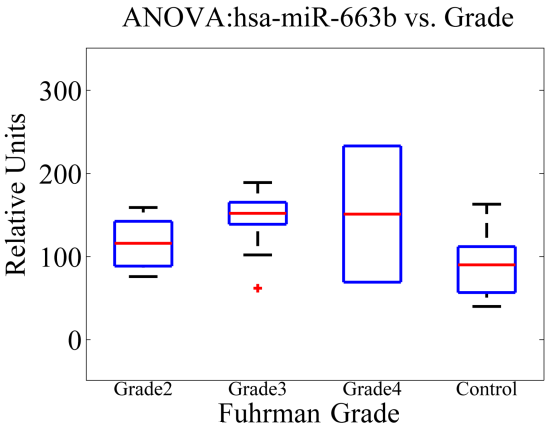

Supplement: Figure S4 — Regression analysis. Regression analysis of the top deregulated miRNAs. One-way ANOVA (left columns) along with linear regression (right columns) of the RCC grades with respect to miRNA expression was used to fit miRNA expression into a linear model. Each regression consists of two curves: one of the form y = ax+b (red) and one of the form y = ax3+bx2+cx+d (blue). All results presented obtained a p<0.05. The median expression values of the miRNAs were used. Interestingly, miR-148b-5p, miR-1910 and miR-200b-5p, miR-210 and miR-3656, presented linear behavior in a descending order of the RCC grade; whereas miR-3687 and miR-654-5p manifested linear behavior in ascending order of the RCC grade. (DOC) [file pone.0091646.s004.doc]

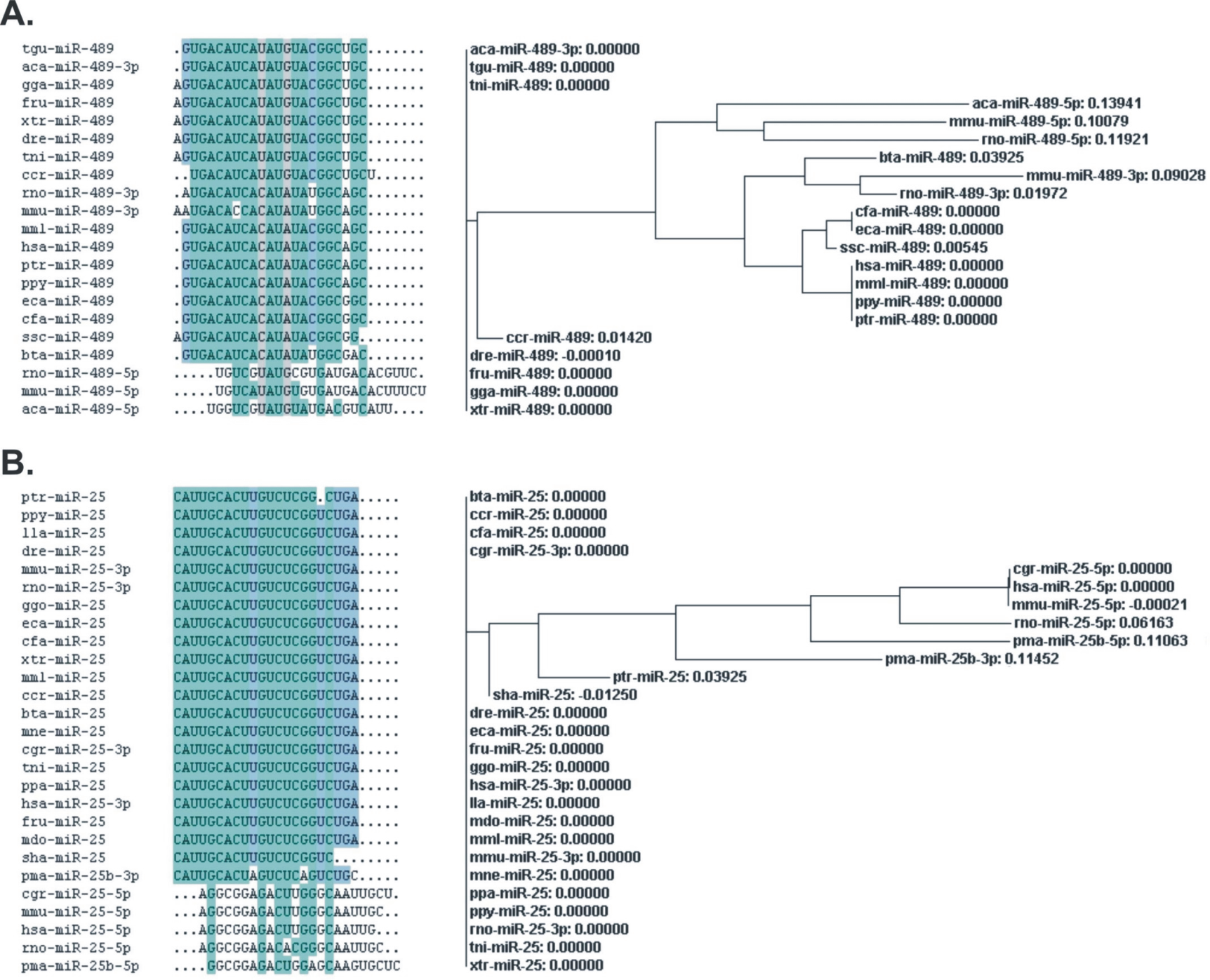

Supplement: Figure S5 — Multiple sequence alignments (MSA). Representative MSA and phylogenetic trees of miR-489 (A) and miR-25-5p (B). The majority of the DE miRNAs (78.45%) were highly conserved among species. Specifically, 70.68% of the up-regulated and 81.22% of the down-regulated miRNAs were phylogenetically conserved among species. (TIF) [file pone.0091646.s005.tif]
