# Supplementary material for: New miRNA Profiles Accurately Distinguish Renal Cell Carcinomas and Upper Tract Urothelial Carcinomas from the Normal Kidney
Source: PLoS One. 2014 Mar 12;9(3):e91646. doi: 10.1371/journal.pone.0091646 (PMC3951427; doi:10.1371/journal.pone.0091646)
Supplement: Table S1 — The table lists the primer sequences that were designed to introduce a SpeI and a HindIII restriction enzyme sites to be cloned into the pMiR-REPORT luciferase vector. (DOC) [file pone.0091646.s006.doc]

**Table S1.** The table lists the primer sequences that were designed to introduce a SpeI and a HindIII restriction enzyme sites to be cloned into the pMiR-REPORT luciferase vector.

| **Primer name** | **Primer sequence (5’- 3’)** |
| --- | --- |
| ABCA1-m23c-CL-F: | ataactagtGCCACTGCCCCACTATTT |
| ABCA1-m23c-CL-R | ataaagcttGGCTCCTACAACCACAAGAC |
| SLC7A8-m204-3p-CL-F | ataactagtCCAATAATCCCCCACTCCTC |
| SLC7A8-m204-3p-CL-R | ataaagcttGGGCTCCTGTGTATTCCAGTT |
| ATP2B4-m520a-5p-CL-F | ataactagtTGGATAAAACAATGGCTGGT |
| ATP2B4-m520a-5p-CL-R | ataaagcttCAAGGGGAAGAAGAAACTCAA |
| TFCP2L1-m489-CL-F | ataactagtGGAATAATGTGCTCAGGGTCA |
| TFCP2L1-m489-CL-R | ataaagcttCATGCCAGAAAAATGGAAGA |
| TRIM10-m874-CL-F | ataactagtCCAGGGACCACACTCAACC |
| TRIM10-m874-CL-R | ataaagcttCTCTTCCATCCCATTTCTCA |
| NR3C1-m513a-3p-CL-F | ataactagtTGGTTTATAGAGGGCCAAGA |
| NR3C1-m513a-3p-CL-R | ataaagcttATCCAGCCAACTGTGAAAAA |
