# Supplementary material for: New miRNA Profiles Accurately Distinguish Renal Cell Carcinomas and Upper Tract Urothelial Carcinomas from the Normal Kidney
Source: PLoS One. 2014 Mar 12;9(3):e91646. doi: 10.1371/journal.pone.0091646 (PMC3951427; doi:10.1371/journal.pone.0091646)
Supplement: Table S2 — Top deregulated miRNAs in ccRCC, papRCC, chRCC and UT-UC vs. the normal kidney tissue. (DOC) [file pone.0091646.s007.doc]

**Table S2.** Top deregulated miRNAs in ccRCC, papRCC, chRCC and UT-UC vs. the normal kidney tissue.

| **ccRCC vs. normal kidney (top 30 up-regulated)** | | | | | | | | | |
| --- | --- | --- | --- | --- | --- | --- | --- | --- | --- |
| **miRNA name** | | **median FC** | | **SD** | | **p-values** | | **FDR** | |
| miR-489 | | 0.8191 | | 0.4648 | | 0.0000 | | 0.0003 | |
| miR-3648 | | 0.7889 | | 0.4029 | | 0.0000 | | 0.0000 | |
| miR-638 | | 0.7637 | | 0.8482 | | 0.0045 | | 0.0136 | |
| miR-3656 | | 0.7627 | | 0.6880 | | 0.0037 | | 0.0122 | |
| miR-25-5p | | 0.6784 | | 0.4525 | | 0.0000 | | 0.0009 | |
| miR-3665 | | 0.6348 | | 0.6812 | | 0.0015 | | 0.0069 | |
| miR-711 | | 0.6315 | | 0.3673 | | 0.0000 | | 0.0009 | |
| miR-3126-5p | | 0.6251 | | 0.3288 | | 0.0000 | | 0.0003 | |
| miR-498 | | 0.6118 | | 0.3587 | | 0.0089 | | 0.0200 | |
| miRPlus-C1087 | | 0.5670 | | 0.3237 | | 0.0006 | | 0.0041 | |
| miR-516b-5p | | 0.5611 | | 0.4059 | | 0.0000 | | 0.0009 | |
| miR-1908 | | 0.5602 | | 0.6243 | | 0.0002 | | 0.0025 | |
| miR-3676-3p | | 0.5514 | | 0.4954 | | 0.0001 | | 0.0019 | |
| miR-4299 | | 0.5401 | | 0.4961 | | 0.0309 | | 0.0412 | |
| miR-3195 | | 0.5240 | | 0.4218 | | 0.0005 | | 0.0033 | |
| miR-1469 | | 0.5104 | | 0.6624 | | 0.0020 | | 0.0084 | |
| miR-204-3p | | 0.5032 | | 0.7876 | | 0.0023 | | 0.0091 | |
| miR-423-5p | | 0.5032 | | 0.3711 | | 0.0002 | | 0.0021 | |
| miR-3687 | | 0.4982 | | 0.6355 | | 0.0000 | | 0.0009 | |
| miR-4290 | | 0.4524 | | 0.3287 | | 0.0004 | | 0.0031 | |
| miR-628-3p | | 0.4412 | | 0.3232 | | 0.0001 | | 0.0015 | |
| miR-1184 | | 0.4216 | | 0.3367 | | 0.0000 | | 0.0003 | |
| miR-4285 | | 0.4198 | | 0.5189 | | 0.0003 | | 0.0031 | |
| miR-3196 | | 0.4193 | | 0.3282 | | 0.0019 | | 0.0083 | |
| miR-210 | | 0.4140 | | 0.2937 | | 0.0001 | | 0.0018 | |
| miR-612 | | 0.4119 | | 0.3290 | | 0.0032 | | 0.0110 | |
| miR-143-3p | | 0.3858 | | 0.2731 | | 0.0009 | | 0.0050 | |
| miR-921 | | 0.3856 | | 0.3609 | | 0.0000 | | 0.0008 | |
| miR-744-5p | | 0.3808 | | 0.4215 | | 0.0345 | | 0.0444 | |
| miR-4317 | | 0.3762 | | 0.2765 | | 0.0002 | | 0.0021 | |
| **ccRCC vs. normal kidney (top 30 down-regulated)** | | | | | | | | | |
| **miRNA name** | | **median FC** | | **SD** | | **p-values** | | **FDR** | |
| miR-4284 | | -1.6009 | | 0.8393 | | 0.0070 | | 0.0175 | |
| miR-1973 | | -1.5405 | | 0.7698 | | 0.0129 | | 0.0242 | |
| miR-140-5p | | -0.5220 | | 0.4226 | | 0.0004 | | 0.0033 | |
| miR-4286 | | -0.5192 | | 0.9717 | | 0.0078 | | 0.0190 | |
| miR-4324 | | -0.5071 | | 0.2390 | | 0.0086 | | 0.0199 | |
| miR-106b-3p | | -0.4876 | | 0.2751 | | 0.0004 | | 0.0032 | |
| miR-23c | | -0.4538 | | 0.2025 | | 0.0000 | | 0.0004 | |
| miR-155-3p | | -0.4269 | | 0.2630 | | 0.0009 | | 0.0050 | |
| miR-551b-5p | | -0.4251 | | 0.2012 | | 0.0003 | | 0.0028 | |
| miR-656 | | -0.4047 | | 0.2198 | | 0.0004 | | 0.0031 | |
| miR-3614-5p | | -0.3890 | | 0.2476 | | 0.0005 | | 0.0037 | |
| miR-145-3p | | -0.3576 | | 0.2147 | | 0.0003 | | 0.0030 | |
| miR-195-3p | | -0.3456 | | 0.3165 | | 0.0415 | | 0.0487 | |
| miR-764 | | -0.3289 | | 0.2260 | | 0.0217 | | 0.0335 | |
| miR-125a-5p | | -0.3289 | | 0.1895 | | 0.0238 | | 0.0362 | |
| miR-1307-5p | | -0.3261 | | 0.1546 | | 0.0000 | | 0.0010 | |
| miR-1257 | | -0.3219 | | 0.1826 | | 0.0000 | | 0.0010 | |
| miR-676-5p | | -0.3194 | | 0.2035 | | 0.0002 | | 0.0025 | |
| miR-493-3p | | -0.3190 | | 0.1818 | | 0.0004 | | 0.0033 | |
| miR-944 | | -0.3099 | | 0.2789 | | 0.0175 | | 0.0295 | |
| miR-520a-5p | | -0.2998 | | 0.1484 | | 0.0451 | | 0.0511 | |
| miR-501-5p | | -0.2981 | | 0.2028 | | 0.0067 | | 0.0171 | |
| miR-624-3p | | -0.2859 | | 0.2352 | | 0.0360 | | 0.0450 | |
| miR-193b-3p | | -0.2836 | | 0.1884 | | 0.0105 | | 0.0214 | |
| miR-4308 | | -0.2816 | | 0.1983 | | 0.0125 | | 0.0236 | |
| let-7f-1-3p | | -0.2792 | | 0.1453 | | 0.0011 | | 0.0056 | |
| miR-570-3p | | -0.2785 | | 0.2240 | | 0.0001 | | 0.0017 | |
| miR-513a-3p/miR-513c-3p | | -0.2734 | | 0.1176 | | 0.0002 | | 0.0024 | |
| miR-3171 | | -0.2696 | | 0.1352 | | 0.0001 | | 0.0017 | |
| miR-520g/miR-520h | | -0.2657 | | 0.1635 | | 0.0003 | | 0.0030 | |
| **papRCC vs. normal kidney (top 30 up-regulated)** | | | | | | | | | |
| **miRNA name** | | **median FC** | | **SD** | | **p-values** | | **FDR** | |
| miR-3687 | | 0.8158 | | 0.1869 | | 0.0000 | | 0.0009 | |
| miR-3648 | | 0.7889 | | 0.2882 | | 0.0000 | | 0.0000 | |
| miR-3656 | | 0.7416 | | 0.5529 | | 0.0037 | | 0.0122 | |
| miR-663b | | 0.7335 | | 0.1686 | | 0.0003 | | 0.0031 | |
| miR-3676-3p | | 0.7175 | | 0.2909 | | 0.0001 | | 0.0019 | |
| miR-3126-5p | | 0.7107 | | 0.2267 | | 0.0000 | | 0.0003 | |
| miRPlus-C1087 | | 0.6322 | | 0.2131 | | 0.0006 | | 0.0041 | |
| miR-4290 | | 0.6076 | | 0.2117 | | 0.0004 | | 0.0031 | |
| miR-204-3p | | 0.5578 | | 0.3156 | | 0.0023 | | 0.0091 | |
| miR-550a-3p | | 0.5482 | | 0.1385 | | 0.0175 | | 0.0295 | |
| miR-489 | | 0.4783 | | 0.3177 | | 0.0000 | | 0.0003 | |
| miR-1276 | | 0.4701 | | 0.2005 | | 0.0001 | | 0.0018 | |
| miR-3195 | | 0.4663 | | 0.2183 | | 0.0005 | | 0.0033 | |
| miR-630 | | 0.4565 | | 0.2214 | | 0.0003 | | 0.0028 | |
| miR-1908 | | 0.4487 | | 0.5687 | | 0.0002 | | 0.0025 | |
| miR-1909-3p | | 0.4158 | | 0.2446 | | 0.0006 | | 0.0041 | |
| miR-1469 | | 0.4111 | | 0.4521 | | 0.0020 | | 0.0084 | |
| miR-3665 | | 0.4064 | | 0.3370 | | 0.0015 | | 0.0069 | |
| miR-4299 | | 0.4044 | | 0.2436 | | 0.0309 | | 0.0412 | |
| miR-1184 | | 0.3861 | | 0.1346 | | 0.0000 | | 0.0003 | |
| miR-214-3p | | 0.3823 | | 0.1175 | | 0.0001 | | 0.0015 | |
| miR-194-3p | | 0.3721 | | 0.2614 | | 0.0001 | | 0.0015 | |
| miR-3614-3p | | 0.3592 | | 0.1457 | | 0.0128 | | 0.0239 | |
| miR-921 | | 0.3579 | | 0.3414 | | 0.0000 | | 0.0008 | |
| miR-4301 | | 0.3509 | | 0.2900 | | 0.0201 | | 0.0320 | |
| miR-3178 | | 0.3257 | | 0.2994 | | 0.0089 | | 0.0200 | |
| miR-371a-5p | | 0.3202 | | 0.1602 | | 0.0000 | | 0.0009 | |
| miR-516b-5p | | 0.3193 | | 0.2187 | | 0.0000 | | 0.0009 | |
| miRPlus-C1076 | | 0.3139 | | 0.1244 | | 0.0032 | | 0.0111 | |
| miR-638 | | 0.3108 | | 0.4509 | | 0.0045 | | 0.0136 | |
| **papRCC vs. normal kidney (top 30 down-regulated)** | | | | | | | | | |
| **miRNA name** | | **median FC** | | **SD** | | **p-values** | | **FDR** | |
| miR-4286 | | -1.2463 | | 0.6594 | | 0.0078 | | 0.0190 | |
| miR-4284 | | -1.1637 | | 0.9942 | | 0.0070 | | 0.0175 | |
| miR-1973 | | -0.6058 | | 0.7811 | | 0.0129 | | 0.0242 | |
| miR-140-5p | | -0.5979 | | 0.0575 | | 0.0004 | | 0.0033 | |
| miR-106b-3p | | -0.4499 | | 0.1360 | | 0.0004 | | 0.0032 | |
| miR-155-3p | | -0.4365 | | 0.1698 | | 0.0009 | | 0.0050 | |
| miR-145-3p | | -0.4150 | | 0.1156 | | 0.0003 | | 0.0030 | |
| miR-23c | | -0.4013 | | 0.1316 | | 0.0000 | | 0.0004 | |
| miR-764 | | -0.3766 | | 0.1642 | | 0.0217 | | 0.0335 | |
| miR-501-5p | | -0.3748 | | 0.1175 | | 0.0067 | | 0.0171 | |
| miR-4276 | | -0.3610 | | 0.0646 | | 0.0005 | | 0.0033 | |
| miR-493-3p | | -0.3337 | | 0.0688 | | 0.0004 | | 0.0033 | |
| miR-95 | | -0.3272 | | 0.1308 | | 0.0146 | | 0.0263 | |
| miR-1257 | | -0.3219 | | 0.0495 | | 0.0000 | | 0.0010 | |
| miR-31-3p | | -0.3175 | | 0.1507 | | 0.0005 | | 0.0033 | |
| miR-194-5p | | -0.3135 | | 0.0815 | | 0.0001 | | 0.0013 | |
| miR-569 | | -0.3090 | | 0.0244 | | 0.0013 | | 0.0067 | |
| miR-183-5p | | -0.3088 | | 0.1333 | | 0.0056 | | 0.0154 | |
| miR-802 | | -0.3015 | | 0.1856 | | 0.0264 | | 0.0377 | |
| miR-520e | | -0.2960 | | 0.1086 | | 0.0000 | | 0.0004 | |
| miR-514b-3p | | -0.2944 | | 0.0764 | | 0.0000 | | 0.0002 | |
| miR-3929 | | -0.2936 | | 0.1122 | | 0.0433 | | 0.0505 | |
| miR-876-3p | | -0.2906 | | 0.1408 | | 0.0007 | | 0.0042 | |
| miR-513a-3p/miR-513c-3p | | -0.2848 | | 0.0384 | | 0.0002 | | 0.0024 | |
| miR-125a-5p | | -0.2839 | | 0.0567 | | 0.0238 | | 0.0362 | |
| miR-187-3p | | -0.2829 | | 0.0383 | | 0.0285 | | 0.0395 | |
| miR-3919 | | -0.2812 | | 0.0770 | | 0.0045 | | 0.0137 | |
| miR-374b-5p | | -0.2808 | | 0.0518 | | 0.0004 | | 0.0032 | |
| miR-570-3p | | -0.2785 | | 0.0624 | | 0.0001 | | 0.0017 | |
| miR-3144-5p | | -0.2775 | | 0.1267 | | 0.0251 | | 0.0367 | |
| **chRCC vs. normal kidney (top 30 up-regulated)** | | | | | | | | |  |
| **miRNA name** | **median FC** | | **SD** | | **p-values** | | **FDR** | |  |
| miR-3656 | 0.7080 | | 0.7028 | | 0.0037 | | 0.0122 | |  |
| miR-663b | 0.6883 | | 0.3119 | | 0.0003 | | 0.0031 | |  |
| miR-193b-5p | 0.6812 | | 0.5454 | | 0.0013 | | 0.0066 | |  |
| miR-489 | 0.6163 | | 0.3277 | | 0.0000 | | 0.0003 | |  |
| miR-638 | 0.5728 | | 1.0402 | | 0.0045 | | 0.0136 | |  |
| miR-3687 | 0.5726 | | 0.4147 | | 0.0000 | | 0.0009 | |  |
| miR-25-5p | 0.5324 | | 0.3670 | | 0.0000 | | 0.0009 | |  |
| miR-3126-5p | 0.5067 | | 0.3495 | | 0.0000 | | 0.0003 | |  |
| miRPlus-C1087 | 0.5030 | | 0.2140 | | 0.0006 | | 0.0041 | |  |
| miR-296-5p | 0.4911 | | 0.3542 | | 0.0182 | | 0.0299 | |  |
| miR-874 | 0.4811 | | 0.3736 | | 0.0004 | | 0.0032 | |  |
| miR-204-3p | 0.4661 | | 0.2996 | | 0.0023 | | 0.0091 | |  |
| miR-3665 | 0.4659 | | 0.6241 | | 0.0015 | | 0.0069 | |  |
| miR-1184 | 0.4642 | | 0.3411 | | 0.0000 | | 0.0003 | |  |
| miR-3648 | 0.4583 | | 0.2988 | | 0.0000 | | 0.0000 | |  |
| miR-1909-3p | 0.4505 | | 0.6010 | | 0.0006 | | 0.0041 | |  |
| miR-654-5p | 0.4392 | | 0.3302 | | 0.0080 | | 0.0192 | |  |
| miR-4290 | 0.3971 | | 0.3921 | | 0.0004 | | 0.0031 | |  |
| miR-550a-3p | 0.3869 | | 0.1334 | | 0.0175 | | 0.0295 | |  |
| miR-1276 | 0.3784 | | 0.2924 | | 0.0001 | | 0.0018 | |  |
| miR-3614-3p | 0.3747 | | 0.1756 | | 0.0128 | | 0.0239 | |  |
| miR-4299 | 0.3698 | | 0.5191 | | 0.0309 | | 0.0412 | |  |
| miR-1228-5p | 0.3571 | | 0.3091 | | 0.0397 | | 0.0471 | |  |
| miR-744-5p | 0.3490 | | 0.6485 | | 0.0345 | | 0.0444 | |  |
| miR-214-3p | 0.3483 | | 0.2906 | | 0.0001 | | 0.0015 | |  |
| miR-1469 | 0.3311 | | 0.7143 | | 0.0020 | | 0.0084 | |  |
| miR-185-3p | 0.3189 | | 0.1789 | | 0.0045 | | 0.0136 | |  |
| miRPlus-C1076 | 0.3104 | | 0.2131 | | 0.0032 | | 0.0111 | |  |
| miR-652-5p | 0.3043 | | 0.3260 | | 0.0049 | | 0.0140 | |  |
| miR-516b-5p | 0.2965 | | 0.3082 | | 0.0000 | | 0.0009 | |  |
| **chRCC vs. normal kidney (top 30 down-regulated)** | | | | | | | | |  |
| **miRNA name** | **median FC** | | **SD** | | **p-values** | | **FDR** | |  |
| miR-3171 | -0.5355 | | 0.2170 | | 0.0001 | | 0.0017 | |  |
| miR-155-3p | -0.4854 | | 0.3481 | | 0.0009 | | 0.0050 | |  |
| miR-140-5p | -0.4854 | | 0.2268 | | 0.0004 | | 0.0033 | |  |
| miR-1973 | -0.4761 | | 0.0833 | | 0.0129 | | 0.0242 | |  |
| miR-620 | -0.4753 | | 0.1814 | | 0.0352 | | 0.0445 | |  |
| miR-23c | -0.4717 | | 0.1410 | | 0.0000 | | 0.0004 | |  |
| miR-551b-5p | -0.4372 | | 0.0314 | | 0.0003 | | 0.0028 | |  |
| miR-764 | -0.4260 | | 0.2651 | | 0.0217 | | 0.0335 | |  |
| miR-520a-5p | -0.3774 | | 0.1988 | | 0.0451 | | 0.0511 | |  |
| miR-1257 | -0.3667 | | 0.0689 | | 0.0000 | | 0.0010 | |  |
| miR-501-5p | -0.3584 | | 0.2151 | | 0.0067 | | 0.0171 | |  |
| miR-520b/miR-520c-3p | -0.3434 | | 0.1676 | | 0.0170 | | 0.0293 | |  |
| miR-145-3p | -0.3352 | | 0.2136 | | 0.0003 | | 0.0030 | |  |
| miR-4286 | -0.3306 | | 0.2512 | | 0.0078 | | 0.0190 | |  |
| miR-1307-5p | -0.3261 | | 0.2177 | | 0.0000 | | 0.0010 | |  |
| miR-944 | -0.3250 | | 0.2816 | | 0.0175 | | 0.0295 | |  |
| miR-106b-3p | -0.3219 | | 0.2040 | | 0.0004 | | 0.0032 | |  |
| miR-3144-5p | -0.3056 | | 0.1942 | | 0.0251 | | 0.0367 | |  |
| miR-99b-3p | -0.2996 | | 0.1761 | | 0.0226 | | 0.0348 | |  |
| miR-590-3p | -0.2880 | | 0.1028 | | 0.0038 | | 0.0125 | |  |
| miR-134 | -0.2795 | | 0.0595 | | 0.0006 | | 0.0041 | |  |
| miR-4276 | -0.2785 | | 0.0866 | | 0.0005 | | 0.0033 | |  |
| miR-554 | -0.2777 | | 0.1080 | | 0.0019 | | 0.0082 | |  |
| miR-4311 | -0.2726 | | 0.2597 | | 0.0192 | | 0.0310 | |  |
| miR-182-3p | -0.2706 | | 0.1991 | | 0.0008 | | 0.0049 | |  |
| miR-676-5p | -0.2692 | | 0.2898 | | 0.0002 | | 0.0025 | |  |
| miR-374a-3p | -0.2555 | | 0.1240 | | 0.0003 | | 0.0028 | |  |
| miR-125a-5p | -0.2530 | | 0.0862 | | 0.0238 | | 0.0362 | |  |
| miR-194-5p | -0.2521 | | 0.0509 | | 0.0001 | | 0.0013 | |  |
| miR-190a | -0.2503 | | 0.1121 | | 0.0099 | | 0.0211 | |  |
| **UT-UC vs. normal kidney (top 30 up-regulated)** | | | | | | | | |  |
| **miRNA name** | **median FC** | | **SD** | | **p-values** | | **FDR** | |  |
| miR-1908 | 1.1864 | | 0.4424 | | 0.0002 | | 0.0025 | |  |
| miR-3196 | 1.1803 | | 0.5214 | | 0.0019 | | 0.0083 | |  |
| miR-3648 | 1.1523 | | 0.3949 | | 0.0000 | | 0.0000 | |  |
| miR-3195 | 1.1388 | | 0.6259 | | 0.0005 | | 0.0033 | |  |
| miR-489 | 0.9937 | | 0.1253 | | 0.0000 | | 0.0003 | |  |
| miR-638 | 0.9496 | | 0.2473 | | 0.0045 | | 0.0136 | |  |
| miR-3665 | 0.9299 | | 0.4118 | | 0.0015 | | 0.0069 | |  |
| miR-204-3p | 0.9133 | | 0.4095 | | 0.0023 | | 0.0091 | |  |
| miR-3687 | 0.8746 | | 0.3441 | | 0.0000 | | 0.0009 | |  |
| miR-663b | 0.8475 | | 0.2274 | | 0.0003 | | 0.0031 | |  |
| miR-3656 | 0.7860 | | 0.6780 | | 0.0037 | | 0.0122 | |  |
| miR-874 | 0.7818 | | 0.1493 | | 0.0004 | | 0.0032 | |  |
| miRPlus-C1087 | 0.7352 | | 0.3096 | | 0.0006 | | 0.0041 | |  |
| miR-3126-5p | 0.7227 | | 0.1477 | | 0.0000 | | 0.0003 | |  |
| miR-4285 | 0.7221 | | 0.0000 | | 0.0003 | | 0.0031 | |  |
| miR-498 | 0.7067 | | 0.2425 | | 0.0089 | | 0.0200 | |  |
| miR-25-5p | 0.6837 | | 0.3263 | | 0.0000 | | 0.0009 | |  |
| miR-423-5p | 0.6673 | | 0.2913 | | 0.0002 | | 0.0021 | |  |
| miR-3676-3p | 0.6440 | | 0.4612 | | 0.0001 | | 0.0019 | |  |
| miR-1909-3p | 0.6194 | | 0.3166 | | 0.0006 | | 0.0041 | |  |
| miR-1469 | 0.6118 | | 0.3221 | | 0.0020 | | 0.0084 | |  |
| miR-516b-5p | 0.6002 | | 0.3849 | | 0.0000 | | 0.0009 | |  |
| miR-711 | 0.5987 | | 0.3612 | | 0.0000 | | 0.0009 | |  |
| miR-921 | 0.5808 | | 0.2265 | | 0.0000 | | 0.0008 | |  |
| miR-1184 | 0.5702 | | 0.0335 | | 0.0000 | | 0.0003 | |  |
| miR-612 | 0.5567 | | 0.1715 | | 0.0032 | | 0.0110 | |  |
| miR-185-3p | 0.5536 | | 0.1405 | | 0.0045 | | 0.0136 | |  |
| miR-1276 | 0.5427 | | 0.2154 | | 0.0001 | | 0.0018 | |  |
| miR-210 | 0.5336 | | 0.1125 | | 0.0001 | | 0.0018 | |  |
| miR-630 | 0.5325 | | 0.1800 | | 0.0003 | | 0.0028 | |  |
| **UT-UC vs. normal kidney (top 30 down-regulated)** | | | | | | | | |  |
| **miRNA name** | | **median FC** | | **SD** | | **p-values** | | **FDR** |  |
| miR-4284 | | -1.9156 | | 1.6687 | | 0.0070 | | 0.0175 |  |
| miR-1973 | | -1.7333 | | 0.9264 | | 0.0129 | | 0.0242 |  |
| miR-4286 | | -0.8253 | | 0.9252 | | 0.0078 | | 0.0190 |  |
| miR-3148 | | -0.6077 | | 0.2966 | | 0.0464 | | 0.0517 |  |
| miR-4324 | | -0.5268 | | 0.1480 | | 0.0086 | | 0.0199 |  |
| miR-140-5p | | -0.5224 | | 0.0811 | | 0.0004 | | 0.0033 |  |
| miR-106b-3p | | -0.4231 | | 0.2016 | | 0.0004 | | 0.0032 |  |
| miR-501-5p | | -0.4038 | | 0.1838 | | 0.0067 | | 0.0171 |  |
| miR-125a-5p | | -0.3819 | | 0.1062 | | 0.0238 | | 0.0362 |  |
| miR-145-3p | | -0.3693 | | 0.3255 | | 0.0003 | | 0.0030 |  |
| miR-656 | | -0.3583 | | 0.1146 | | 0.0004 | | 0.0031 |  |
| miR-99b-3p | | -0.3482 | | 0.1551 | | 0.0226 | | 0.0348 |  |
| miR-23c | | -0.3383 | | 0.1546 | | 0.0000 | | 0.0004 |  |
| miR-676-5p | | -0.3380 | | 0.1919 | | 0.0002 | | 0.0025 |  |
| miR-155-3p | | -0.3282 | | 0.3068 | | 0.0009 | | 0.0050 |  |
| miR-518e-3p | | -0.3064 | | 0.1056 | | 0.0016 | | 0.0073 |  |
| miR-944 | | -0.2803 | | 0.2866 | | 0.0175 | | 0.0295 |  |
| miR-764 | | -0.2743 | | 0.2374 | | 0.0217 | | 0.0335 |  |
| miR-1226-3p | | -0.2707 | | 0.0538 | | 0.0093 | | 0.0203 |  |
| miR-195-3p | | -0.2564 | | 0.1335 | | 0.0415 | | 0.0487 |  |
| miR-513a-3p/miR-513c-3p | | -0.2509 | | 0.0434 | | 0.0002 | | 0.0024 |  |
| miR-551b-5p | | -0.2491 | | 0.0993 | | 0.0003 | | 0.0028 |  |
| miR-577 | | -0.2402 | | 0.0610 | | 0.0083 | | 0.0195 |  |
| miR-4308 | | -0.2376 | | 0.1410 | | 0.0125 | | 0.0236 |  |
| miRPlus-J1003 | | -0.2371 | | 0.1220 | | 0.0258 | | 0.0372 |  |
| miR-570-3p | | -0.2325 | | 0.0124 | | 0.0001 | | 0.0017 |  |
| miR-199a-5p | | -0.2287 | | 0.0609 | | 0.0060 | | 0.0161 |  |
| miR-1324 | | -0.2287 | | 0.1901 | | 0.0043 | | 0.0134 |  |
| miR-514b-3p | | -0.2280 | | 0.0212 | | 0.0000 | | 0.0002 |  |
| miR-520g/miR-520h | | -0.2245 | | 0.0773 | | 0.0003 | | 0.0030 |  |
