# Supplementary material for: New miRNA Profiles Accurately Distinguish Renal Cell Carcinomas and Upper Tract Urothelial Carcinomas from the Normal Kidney
Source: PLoS One. 2014 Mar 12;9(3):e91646. doi: 10.1371/journal.pone.0091646 (PMC3951427; doi:10.1371/journal.pone.0091646)
Supplement: Table S3 — Co-deregulated and tissue specific miRNAs in ccRCC, papRCC, chRCC and UT-UC. Co-upregulated and tissue specific miRNAs in RCC subtypes (A). Co-downregulated and tissue specific miRNAs in RCC subtypes (B). Co-upregulated and tissue specific miRNAs in RCC and UT-UC (C). Co-downregulated and tissue specific miRNAs in RCC and UT-UC (D). (DOC) [file pone.0091646.s008.doc]

**Table S3.** Co-deregulated and tissue specific miRNAs in ccRCC, papRCC, chRCC and UT-UC. Co-upregulated and tissue specific miRNAs in RCC subtypes (A). Co-downregulated and tissue specific miRNAs in RCC subtypes (B). Co-upregulated and tissue specific miRNAs in RCC and UT-UC (C). Co-downregulated and tissue specific miRNAs in RCC and UT-UC (D).

1. **Up-regulated miRNAs in RCC subtypes**.

| **Common elements in "papRCC", "ccRCC" and "chRCC":** |
| --- |
| miR-3687 |
| miR-3648 |
| miR-3656 |
| miR-663b |
| miR-3676-3p |
| miR-3126-5p |
| miRPlus-C1087 |
| miR-4290 |
| miR-204-3p |
| miR-550a-3p |
| miR-489 |
| miR-1276 |
| miR-3195 |
| miR-630 |
| miR-1908 |
| miR-1909-3p |
| miR-1469 |
| miR-3665 |
| miR-4299 |
| miR-1184 |
| miR-214-3p |
| miR-194-3p |
| miR-3614-3p |
| miR-921 |
| miR-4301 |
| miR-371a-5p |
| miR-516b-5p |
| miRPlus-C1076 |
| miR-638 |
| miR-193b-5p |
| miR-25-5p |
| miR-3135a |
| miR-4317 |
| miR-210 |
| miR-612 |
| miR-519e-5p |
| miR-498 |
| miR-874 |
| miR-628-3p |
| miR-885-5p |
| miR-365a-5p/miR-365b-5p |
| miR-421 |
| miR-4273 |
| miR-1228-5p |
| miR-200b-5p |
| miR-4285 |
| miR-323a-3p |
| miR-3606 |
| miRPlus-C1110 |
| miR-769-3p |
| miR-492 |
| miR-3944-3p |
| miRPlus-B1114 |
| miR-1207-3p |
| miR-185-3p |
| miR-711 |
| miR-2115-5p |
| miR-296-5p |
| miR-3655 |
| miR-654-5p |
| miR-143-3p |
| miR-585 |
| miR-103a-3p |
| miR-744-5p |
| miR-2861 |
| miR-575 |
| miR-571 |
| miR-3937 |
| miR-652-5p |
| miR-373-5p |
| miR-24-3p |
| miR-762 |
| miR-219-2-3p |
| miR-4325 |
| miR-675-5p |
| miR-3922-3p |
| miR-663a |
| miR-147b |
| miR-124-5p |
| miR-298 |
| miR-130b-3p |
| miR-1912 |
| miR-423-5p |
| miR-3065-5p |
| miR-3127-5p |
| miR-3177-3p |
| miR-27a-5p |
| miR-205-5p |
| miR-26a-2-3p |
| miR-129-5p |
| miR-639 |
| miR-378a-5p |
| miR-4315 |
| miR-10a-3p |

| **Common elements in "papRCC" and "ccRCC":** |
| --- |
| miR-3196 |
| miR-3607-5p |
| miRPlus-K1303* |
| miR-1247-3p |
| miR-1281 |
| miR-363-5p |
| miR-2277-5p |
| miR-483-5p |
| miR-150-3p |
| miR-224-5p |
| miR-423-3p |
| miR-203 |
| miR-3917 |
| miR-516a-5p |
| miR-3124-5p |
| miR-302d-3p |
| miR-452-5p |
| miR-3186-3p |

| **Common elements in "papRCC" and "chRCC":** |
| --- |
| miR-551a |
| miR-127-3p |
| miR-34c-3p |
| miR-940 |
| miRPlus-J1003 |
| miR-132-3p |
| miR-379-3p |
| miR-519a-3p |
| miR-422a |
| miR-525-3p |
| miR-3143 |
| miR-1538 |
| miR-186-3p |
| miR-505-3p |
| miR-2117 |
| miR-4308 |

| **Common elements in "ccRCC" and "chRCC":** |
| --- |
| miR-92b-5p |
| miR-2110 |
| miR-635 |
| miR-3692-3p |
| miR-3654 |

| **Elements only in "papRCC":** |
| --- |
| miR-3178 |
| miR-520a-5p |
| miR-3148 |
| miR-518e-3p |
| miR-3161 |
| miR-346 |
| miR-1324 |
| miR-1206 |
| miR-520d-5p |
| miR-144-3p |
| miR-518c-3p |
| miR-376a-3p |
| miR-200a-3p |
| miR-567 |
| miR-876-5p |
| miR-374a-5p |
| miR-19a-5p |
| miR-526b-3p |
| miRPlus-G1140-3p |
| miR-662 |
| miR-523-3p |
| miR-3074-3p |
| miR-33b-5p |
| miR-548t-5p |

| **Elements only in "ccRCC":** | |
| --- | --- |
| miR-30c-1-3p |  |
| miR-3945 |  |
| miR-4251 |  |
| miR-1321 |  |
| miR-409-3p |  |
| miR-4796-3p |  |
| miR-548f |  |
| miR-548c-3p |  |
| miR-32-5p |  |
| miR-3909 |  |
| miR-27a-3p |  |

| **Elements only in "chRCC":** |
| --- |
| miR-95 |
| miR-30d-3p |
| miR-504 |
| miR-2277-3p |
| miR-4284 |
| miR-10b-3p |
| miR-586 |
| miR-4293 |
| miR-145-5p |
| miR-873-5p |
| miR-562 |
| miR-942 |
| miR-3151 |
| miR-345-5p |
| miR-1909-5p |
| miR-448 |
| miR-3663-3p |
| miR-1197 |
| miR-3657 |
| miR-548p |
| miR-454-5p |
| miRPlus-G1079-5p |
| miR-548u |
| miR-1200 |
| miR-193a-3p |
| miR-3168 |
| miR-591 |
| miR-543 |
| miR-545-5p |
| miR-105-3p |
| miR-31-5p |
| miR-28-3p |
| miR-522-3p |
| miR-4262 |
| miR-1256 |
| miR-494 |
| miR-363-3p |
| miR-186-5p |
| miR-3128 |
| miR-520g |
| miR-196b-3p |
| miR-148a-5p |
| miR-4310 |
| miR-596 |

1. **Down-regulated miRNAs in RCC subtypes**.

| **Common elements in "papRCC", "ccRCC" and "chRCC":** |
| --- |
| miR-199b-5p |
| miR-548w |
| miR-548a-3p |
| miR-520b/miR-520c-3p |
| miR-1288 |
| miR-518d-3p |
| miR-561-5p |
| miRPlus-J135a-2* |
| miR-106a-5p |
| miR-603 |
| miR-495 |
| miR-643 |
| miR-541-5p |
| miR-3065-3p |
| miR-548d-5p |
| miR-744-3p |
| miR-617 |
| miR-30a-5p |
| miR-181a-3p |
| miR-520f |
| miR-409-5p |
| miR-606 |
| miR-3615 |
| miR-34a-3p |
| miR-429 |
| miR-944 |
| miR-514a-3p |
| miR-1185-5p |
| miRPlus-C1057 |
| miR-302e |
| miR-142-5p |
| miR-548aa/miR-548t-3p |
| miR-103a-2-5p |
| miR-153 |
| miR-548j |
| miR-218-5p |
| miR-2052 |
| miR-1283 |
| miR-191-5p |
| miR-520c-3p |
| miR-3614-5p |
| miR-154-5p |
| miR-190a |
| miR-4282 |
| miR-656 |
| miR-1234 |
| miR-548l |
| miR-3144-3p |
| miR-3674 |
| miR-888-5p |
| miR-338-5p |
| miR-582-5p |
| miR-558 |
| miR-521 |
| miR-1193 |
| miR-181d |
|  |
| miR-514b-5p |
| miR-135a-5p |
| miR-1226-5p |
| miR-342-3p |
| miR-2909 |
| miR-769-5p |
| miR-4266 |
| miR-222-5p |
| miR-548e |
| miR-323b-5p |
| miR-566 |
| miR-34c-5p |
| miR-759 |
| miR-2116-3p |
| miR-511 |
| miR-3158-3p |
| miR-1238 |
| miR-518f-5p |
| miR-501-3p |
| miR-4311 |
| miR-148b-5p |
| miR-3187-3p |
| miR-362-3p |
| miR-520g/miR-520h |
| miR-577 |
| miR-412 |
| miR-369-3p |
| miR-1248 |
| miR-375 |
| miR-587 |
| miR-411-5p |
| miR-1226-3p |
| miR-616-5p |
| miRPlus-C1056 |
| miR-500a-5p/miR-500b |
| miR-3927 |
| miR-449a |
| miR-182-5p |
| miR-98 |
| miR-582-3p |
| miR-649 |
| miR-549 |
| miR-329 |
| miR-579 |
| miR-410 |
| miR-641 |
| miR-146a-5p |
| miR-411-3p |
| miR-4328 |
| miR-450b-5p |
| miR-576-3p |
| miR-548n |
| miR-3180-3p |
| miR-3157-5p |
| miR-654-3p |
| miR-607 |
| miR-195-3p |
| miR-3118 |
| miR-548y |
| miR-199a-5p |
| miR-1208 |
| miR-1323 |
| miR-548d-3p |
| miR-548g-3p |
| miR-212-5p |
| miR-548i |
| miR-192-5p |
| miR-515-5p |
| miR-193b-3p |
| miR-941 |
| miR-3064-3p |
| miR-518d-5p/miR-520c-5p/miR-526a |
| miR-4324 |
| miR-200c-5p |
| miR-2964a-5p |
| miR-3616-3p |
| miR-138-5p |
| miR-342-5p |
| miR-101-3p |
| miR-376b |
| miR-3164 |
| miR-496 |
| miR-136-5p |
| miR-493-5p |
| miR-3194-5p |
| miR-3181 |
| miR-624-3p |
| miR-211-5p |
| miR-4269 |
| miR-875-3p |
| miR-3920 |
| miR-381 |
| miR-590-3p |
| miR-3176 |
| miR-3668 |
| miR-1251 |
| miR-134 |
| miR-137 |
| miR-199a-3p/miR-199b-3p |
| miR-29b-3p |
| miR-3669 |
| miR-3117-3p |
| let-7f-1-3p |
| miR-644a |
| miR-122-5p |
| miR-518f-3p |
| miR-1910 |
| miR-10b-5p |
| miR-497-5p |
| miR-182-3p |
| miR-548am-5p/miR-548c-5p/miR-548o-5p |
| miR-646 |
| miR-3672 |
| miR-20a-3p |
| miR-146b-5p |
| miR-554 |
| miR-4256 |
| miR-1262 |
| miR-92a-3p |
| miR-337-3p |
| miR-206 |
| miR-605 |
| miR-519d |
| miR-99b-3p |
| miR-875-5p |
| let-7c |
| miR-620 |
| miR-1303 |
| miR-1307-5p |
| miR-551b-5p |
| miR-24-2-5p |
| miR-614 |
| miR-374a-3p |
| miR-676-5p |
| miR-3171 |
| miR-889 |
| miR-4270 |
| miR-3144-5p |
| miR-570-3p |
| miR-374b-5p |
| miR-3919 |
| miR-187-3p |
| miR-125a-5p |
| miR-513a-3p/miR-513c-3p |
| miR-876-3p |
| miR-3929 |
| miR-514b-3p |
| miR-520e |
| miR-802 |
| miR-183-5p |
| miR-569 |
| miR-194-5p |
| miR-31-3p |
| miR-1257 |
| miR-493-3p |
| miR-4276 |
| miR-501-5p |
| miR-764 |
| miR-23c |
| miR-145-3p |
| miR-155-3p |
| miR-106b-3p |
| miR-140-5p |
| miR-1973 |
| miR-4286 |

| **Common elements in "papRCC" and "ccRCC":** |
| --- |
| miR-448 |
| miR-196b-3p |
| miR-1197 |
| miR-548u |
| miR-3657 |
| miR-2277-3p |
| miR-548p |
| miR-591 |
| miR-148a-5p |
| miR-545-5p |
| miR-4262 |
| miR-586 |
| miR-1200 |
| miR-105-3p |
| miR-873-5p |
| miR-543 |
| miR-30d-3p |
| miR-1909-5p |
| miR-1256 |
| miR-145-5p |
| miR-4293 |
| miR-3168 |
| miR-504 |
| miR-10b-3p |
| miR-186-5p |
| miR-28-3p |
| miR-363-3p |
| miR-520g |
| miR-3663-3p |
| miR-345-5p |
| miR-494 |
| miR-31-5p |
| miR-193a-3p |
| miR-942 |
| miR-4310 |
| miR-522-3p |
| miR-562 |
| miR-596 |
| miR-3151 |
| miR-454-5p |
| miRPlus-G1079-5p |
| miR-3128 |
| miR-95 |
| miR-4284 |

| **Common elements in "papRCC" and "chRCC":** |
| --- |
| miR-4251 |
| miR-30c-1-3p |
| miR-548f |
| miR-409-3p |
| miR-4796-3p |
| miR-3945 |
| miR-27a-3p |
| miR-1321 |
| miR-3909 |
| miR-548c-3p |
| miR-32-5p |

| **Common elements in "ccRCC" and "chRCC":** |
| --- |
| miR-376a-3p |
| miR-3178 |
| miR-19a-5p |
| miRPlus-G1140-3p |
| miR-3074-3p |
| miR-200a-3p |
| miR-374a-5p |
| miR-567 |
| miR-1206 |
| miR-346 |
| miR-876-5p |
| miR-33b-5p |
| miR-662 |
| miR-518e-3p |
| miR-1324 |
| miR-526b-3p |
| miR-144-3p |
| miR-518c-3p |
| miR-520d-5p |
| miR-523-3p |
| miR-3148 |
| miR-548t-5p |
| miR-3161 |
| miR-520a-5p |

| **Elements only in "papRCC":** |
| --- |
| miR-92b-5p |
| miR-635 |
| miR-2110 |
| miR-3692-3p |
| miR-3654 |

| **Elements only in "ccRCC":** | |
| --- | --- |
| miR-551a |  |
| miR-422a |  |
| miR-525-3p |  |
| miR-2117 |  |
| miR-127-3p |  |
| miR-132-3p |  |
| miR-505-3p |  |
| miR-940 |  |
| miR-186-3p |  |
| miR-34c-3p |  |
| miR-519a-3p |  |
| miR-379-3p |  |
| miR-1538 |  |
| miR-3143 |  |
| miRPlus-J1003 |  |
| miR-4308 |  |

| **Elements only in "chRCC":** |
| --- |
| miR-1281 |
| miR-224-5p |
| miR-150-3p |
| miR-3917 |
| miR-363-5p |
| miRPlus-K1303* |
| miR-1247-3p |
| miR-2277-5p |
| miR-3124-5p |
| miR-302d-3p |
| miR-483-5p |
| miR-3607-5p |
| miR-203 |
| miR-423-3p |
| miR-3196 |
| miR-3186-3p |
| miR-452-5p |
| miR-516a-5p |

1. **Up-regulated miRNAs in RCC and UT-UC**.

| **Common elements in "papRCC", "ccRCC", "chRCC" and "UT-UC":** |
| --- |
| miR-3687 |
| miR-3648 |
| miR-3656 |
| miR-663b |
| miR-3676-3p |
| miR-3126-5p |
| miRPlus-C1087 |
| miR-4290 |
| miR-204-3p |
| miR-550a-3p |
| miR-489 |
| miR-1276 |
| miR-3195 |
| miR-630 |
| miR-1908 |
| miR-1909-3p |
| miR-1469 |
| miR-3665 |
| miR-4299 |
| miR-1184 |
| miR-214-3p |
| miR-194-3p |
| miR-3614-3p |
| miR-921 |
| miR-4301 |
| miR-371a-5p |
| miR-516b-5p |
| miRPlus-C1076 |
| miR-638 |
| miR-193b-5p |
| miR-25-5p |
| miR-3135a |
| miR-4317 |
| miR-210 |
| miR-612 |
| miR-519e-5p |
| miR-498 |
| miR-874 |
| miR-628-3p |
| miR-365a-5p/miR-365b-5p |
| miR-421 |
| miR-4273 |
| miR-1228-5p |
| miR-200b-5p |
| miR-4285 |
| miR-323a-3p |
| miR-3606 |
| miRPlus-C1110 |
| miR-769-3p |
| miR-492 |
| miR-3944-3p |
| miRPlus-B1114 |
| miR-1207-3p |
| miR-185-3p |
| miR-711 |
| miR-2115-5p |
| miR-296-5p |
| miR-3655 |
| miR-654-5p |
| miR-143-3p |
| miR-585 |
| miR-103a-3p |
| miR-744-5p |
| miR-2861 |
| miR-575 |
| miR-571 |
| miR-3937 |
| miR-652-5p |
| miR-373-5p |
| miR-762 |
| miR-219-2-3p |
| miR-4325 |
| miR-675-5p |
| miR-3922-3p |
| miR-663a |
| miR-147b |
| miR-124-5p |
| miR-298 |
| miR-130b-3p |
| miR-423-5p |
| miR-3065-5p |
| miR-3127-5p |
| miR-3177-3p |
| miR-27a-5p |
| miR-205-5p |
| miR-129-5p |
| miR-639 |
| miR-4315 |
| miR-10a-3p |

| **Common elements in "papRCC", "ccRCC" and "UT-UC":** |
| --- |
| miR-3196 |
| miR-3607-5p |
| miRPlus-K1303* |
| miR-1247-3p |
| miR-1281 |
| miR-2277-5p |
| miR-483-5p |
| miR-224-5p |
| miR-423-3p |
| miR-203 |
| miR-3917 |
| miR-516a-5p |
| miR-3124-5p |
| miR-452-5p |
| miR-3186-3p |

| **Common elements in "papRCC", "chRCC" and "UT-UC":** | |
| --- | --- |
| miR-551a |  |
| miR-34c-3p |  |
| miR-940 |  |
| miR-422a |  |

| **Common elements in "papRCC" and "UT-UC":** |
| --- |
| miR-3178 |

| **Common elements in "ccRCC" and "UT-UC":** |
| --- |
| miR-30c-1-3p |
| miR-3945 |
| miR-4251 |
| miR-1321 |
| miR-409-3p |
| miR-4796-3p |
| miR-32-5p |

| **Common elements in "ccRCC", "chRCC" and "UT-UC":** |
| --- |
| miR-92b-5p |
| miR-2110 |
| miR-635 |
| miR-3692-3p |
| miR-3654 |

| **Common elements in "papRCC" and "chRCC":** |
| --- |
| miR-127-3p |
| miRPlus-J1003 |
| miR-132-3p |
| miR-379-3p |
| miR-519a-3p |
| miR-525-3p |
| miR-3143 |
| miR-1538 |
| miR-186-3p |
| miR-505-3p |
| miR-2117 |
| miR-4308 |

| **Common elements in "papRCC", "ccRCC" and "chRCC":** |
| --- |
| miR-885-5p |
| miR-24-3p |
| miR-1912 |
| miR-26a-2-3p |
| miR-378a-5p |

| **Common elements in "ccRCC" and "chRCC":** |
| --- |
|  |

| **Elements only in "UT-UC":** | |
| --- | --- |
| miR-3144-5p |  |
| miR-193b-3p |  |
| miR-587 |  |
| miR-3117-3p |  |
| miR-769-5p |  |
| miR-617 |  |
| miR-3615 |  |
| miR-148b-5p |  |
| miR-24-2-5p |  |
| miR-142-5p |  |
| miR-3164 |  |
| miR-514b-5p |  |
| miR-375 |  |
| miR-520c-3p |  |
| miR-191-5p |  |
| miR-520b/miR-520c-3p |  |

| **Common elements in "chRCC" and "UT-UC":** | |
| --- | --- |
| miR-4293 |  |
| miR-3663-3p |  |
| miR-3168 |  |
| miR-494 |  |
| miR-4310 |  |

| **Elements only in "papRCC":** |
| --- |
| miR-520a-5p |
| miR-3148 |
| miR-518e-3p |
| miR-3161 |
| miR-346 |
| miR-1324 |
| miR-1206 |
| miR-520d-5p |
| miR-144-3p |
| miR-518c-3p |
| miR-376a-3p |
| miR-200a-3p |
| miR-567 |
| miR-876-5p |
| miR-374a-5p |
| miR-19a-5p |
| miR-526b-3p |
| miRPlus-G1140-3p |
| miR-662 |
| miR-523-3p |
| miR-3074-3p |
| miR-33b-5p |
| miR-548t-5p |

| **Common elements in "papRCC" and "ccRCC":** |
| --- |
| miR-363-5p |
| miR-150-3p |
| miR-302d-3p |

| **Elements only in "chRCC":** |
| --- |
| miR-95 |
| miR-30d-3p |
| miR-504 |
| miR-2277-3p |
| miR-4284 |
| miR-10b-3p |
| miR-586 |
| miR-145-5p |
| miR-873-5p |
| miR-562 |
| miR-942 |
| miR-3151 |
| miR-345-5p |
| miR-1909-5p |
| miR-448 |
| miR-1197 |
| miR-3657 |
| miR-548p |
| miR-454-5p |
| miRPlus-G1079-5p |
| miR-548u |
| miR-1200 |
| miR-193a-3p |
| miR-591 |
| miR-543 |
| miR-545-5p |
| miR-105-3p |
| miR-31-5p |
| miR-28-3p |
| miR-522-3p |
| miR-4262 |
| miR-1256 |
| miR-363-3p |
| miR-186-5p |
| miR-3128 |
| miR-520g |
| miR-196b-3p |
| miR-148a-5p |
| miR-596 |

| **Elements only in "ccRCC":** | |
| --- | --- |
| miR-548f |  |
| miR-548c-3p |  |
| miR-3909 |  |
| miR-27a-3p |  |

1. **Down-regulated miRNAs in RCC and UT-UC**.

| **Common elements in "papRCC", "ccRCC", "chRCC" and "UT-UC":** | |
| --- | --- |
| miR-199b-5p |  |
| miR-548w |  |
| miR-548a-3p |  |
| miR-1288 |  |
| miR-518d-3p |  |
| miR-561-5p |  |
| miRPlus-J135a-2* |  |
| miR-106a-5p |  |
| miR-603 |  |
| miR-495 |  |
| miR-643 |  |
| miR-541-5p |  |
| miR-3065-3p |  |
| miR-548d-5p |  |
| miR-744-3p |  |
| miR-30a-5p |  |
| miR-181a-3p |  |
| miR-520f |  |
| miR-409-5p |  |
| miR-606 |  |
| miR-34a-3p |  |
| miR-429 |  |
| miR-944 |  |
| miR-514a-3p |  |
| miR-1185-5p |  |
| miRPlus-C1057 |  |
| miR-302e |  |
| miR-548aa/miR-548t-3p |  |
| miR-103a-2-5p |  |
| miR-153 |  |
| miR-548j |  |
| miR-218-5p |  |
| miR-2052 |  |
| miR-1283 |  |
| miR-3614-5p |  |
| miR-154-5p |  |
| miR-190a |  |
| miR-4282 |  |
| miR-656 |  |
| miR-1234 |  |
| miR-548l |  |
| miR-3144-3p |  |
| miR-3674 |  |
| miR-888-5p |  |
| miR-338-5p |  |
| miR-582-5p |  |
| miR-558 |  |
| miR-521 |  |
| miR-1193 |  |
| miR-181d |  |
| miR-135a-5p |  |
| miR-1226-5p |  |
| miR-342-3p |  |
| miR-2909 |  |
| miR-4266 |  |
| miR-222-5p |  |
| miR-548e |  |
| miR-323b-5p |  |
| miR-566 |  |
| miR-34c-5p |  |
| miR-759 |  |
| miR-2116-3p |  |
| miR-511 |  |
| miR-3158-3p |  |
| miR-1238 |  |
| miR-518f-5p |  |
| miR-501-3p |  |
| miR-4311 |  |
| miR-3187-3p |  |
| miR-362-3p |  |
| miR-520g/miR-520h |  |
| miR-577 |  |
| miR-412 |  |
| miR-369-3p |  |
| miR-1248 |  |
| miR-411-5p |  |
| miR-1226-3p |  |
| miR-616-5p |  |
| miRPlus-C1056 |  |
| miR-500a-5p/miR-500b |  |
| miR-3927 |  |
| miR-449a |  |
| miR-182-5p |  |
| miR-98 |  |
| miR-582-3p |  |
| miR-649 |  |
| miR-549 |  |
| miR-329 |  |
| miR-579 |  |
| miR-410 |  |
| miR-641 |  |
| miR-146a-5p |  |
| miR-411-3p |  |
| miR-4328 |  |
| miR-450b-5p |  |
| miR-576-3p |  |
| miR-548n |  |
| miR-3180-3p |  |
| miR-3157-5p |  |
| miR-654-3p |  |
| miR-607 |  |
| miR-195-3p |  |
| miR-3118 |  |
| miR-548y |  |
| miR-199a-5p |  |
| miR-1208 |  |
| miR-1323 |  |
| miR-548d-3p |  |
| miR-548g-3p |  |
| miR-212-5p |  |
| miR-548i |  |
| miR-192-5p |  |
| miR-515-5p |  |
| miR-941 |  |
| miR-3064-3p |  |
| miR-518d-5p/miR-520c-5p/miR-526a |  |
| miR-4324 |  |
| miR-200c-5p |  |
| miR-2964a-5p |  |
| miR-3616-3p |  |
| miR-138-5p |  |
| miR-342-5p |  |
| miR-101-3p |  |
| miR-376b |  |
| miR-496 |  |
| miR-136-5p |  |
| miR-493-5p |  |
| miR-3194-5p |  |
| miR-3181 |  |
| miR-624-3p |  |
| miR-211-5p |  |
| miR-4269 |  |
| miR-875-3p |  |
| miR-3920 |  |
| miR-381 |  |
| miR-590-3p |  |
| miR-3176 |  |
| miR-3668 |  |
| miR-1251 |  |
| miR-134 |  |
| miR-137 |  |
| miR-199a-3p/miR-199b-3p |  |
| miR-29b-3p |  |
| miR-3669 |  |
| let-7f-1-3p |  |
| miR-644a |  |
| miR-122-5p |  |
| miR-518f-3p |  |
| miR-1910 |  |
| miR-10b-5p |  |
| miR-497-5p |  |
| miR-182-3p |  |
| miR-548am-5p/miR-548c-5p/miR-548o-5p |  |
| miR-646 |  |
| miR-3672 |  |
| miR-20a-3p |  |
| miR-146b-5p |  |
| miR-554 |  |
| miR-4256 |  |
| miR-1262 |  |
| miR-92a-3p |  |
| miR-337-3p |  |
| miR-206 |  |
| miR-605 |  |
| miR-519d |  |
| miR-99b-3p |  |
| miR-875-5p |  |
| let-7c |  |
| miR-620 |  |
| miR-1303 |  |
| miR-1307-5p |  |
| miR-551b-5p |  |
| miR-614 |  |
| miR-374a-3p |  |
| miR-676-5p |  |
| miR-3171 |  |
| miR-889 |  |
| miR-4270 |  |
| miR-570-3p |  |
| miR-374b-5p |  |
| miR-3919 |  |
| miR-187-3p |  |
| miR-125a-5p |  |
| miR-513a-3p/miR-513c-3p |  |
| miR-876-3p |  |
| miR-3929 |  |
| miR-514b-3p |  |
| miR-520e |  |
| miR-802 |  |
| miR-183-5p |  |
| miR-569 |  |
| miR-194-5p |  |
| miR-31-3p |  |
| miR-1257 |  |
| miR-493-3p |  |
| miR-4276 |  |
| miR-501-5p |  |
| miR-764 |  |
| miR-23c |  |
| miR-145-3p |  |
| miR-155-3p |  |
| miR-106b-3p |  |
| miR-140-5p |  |
| miR-1973 |  |
| miR-4286 |  |

| **Common elements in "papRCC", "ccRCC" and "UT-UC":** |
| --- |
| miR-448 |
| miR-196b-3p |
| miR-1197 |
| miR-548u |
| miR-3657 |
| miR-2277-3p |
| miR-548p |
| miR-591 |
| miR-148a-5p |
| miR-545-5p |
| miR-4262 |
| miR-586 |
| miR-1200 |
| miR-105-3p |
| miR-873-5p |
| miR-543 |
| miR-30d-3p |
| miR-1909-5p |
| miR-1256 |
| miR-145-5p |
| miR-504 |
| miR-10b-3p |
| miR-186-5p |
| miR-28-3p |
| miR-363-3p |
| miR-520g |
| miR-345-5p |
| miR-31-5p |
| miR-193a-3p |
| miR-942 |
| miR-522-3p |
| miR-562 |
| miR-596 |
| miR-3151 |
| miR-454-5p |
| miRPlus-G1079-5p |
| miR-3128 |
| miR-95 |
| miR-4284 |

**Common elements in "papRCC" and "UT-UC":**

| **Common elements in "papRCC", "chRCC" and "UT-UC":** |
| --- |
| miR-548f |
| miR-27a-3p |
| miR-3909 |
| miR-548c-3p |

| **Common elements in "ccRCC" and "UT-UC":** |
| --- |
| miR-525-3p |
| miR-2117 |
| miR-127-3p |
| miR-132-3p |
| miR-505-3p |
| miR-186-3p |
| miR-519a-3p |
| miR-379-3p |
| miR-1538 |
| miR-3143 |
| miRPlus-J1003 |
| miR-4308 |

| **Elements only in "UT-UC":** |
| --- |
| miR-885-5p |
| miR-1912 |
| miR-378a-5p |
| miR-24-3p |
| miR-26a-2-3p |

| **Common elements in "ccRCC", "chRCC" and "UT-UC":** |
| --- |
| miR-376a-3p |
| miR-19a-5p |
| miRPlus-G1140-3p |
| miR-3074-3p |
| miR-200a-3p |
| miR-374a-5p |
| miR-567 |
| miR-1206 |
| miR-346 |
| miR-876-5p |
| miR-33b-5p |
| miR-662 |
| miR-518e-3p |
| miR-1324 |
| miR-526b-3p |
| miR-144-3p |
| miR-518c-3p |
| miR-520d-5p |
| miR-523-3p |
| miR-3148 |
| miR-548t-5p |
| miR-3161 |
| miR-520a-5p |

| **Common elements in "chRCC" and "UT-UC":** |
| --- |
| miR-150-3p |
| miR-363-5p |
| miR-302d-3p |

| **Common elements in "papRCC" and "chRCC":** |
| --- |
| miR-4251 |
| miR-30c-1-3p |
| miR-409-3p |
| miR-4796-3p |
| miR-3945 |
| miR-1321 |
| miR-32-5p |

| **Elements only in "papRCC":** |
| --- |
| miR-92b-5p |
| miR-635 |
| miR-2110 |
| miR-3692-3p |
| miR-3654 |

| **Common elements in "papRCC", "ccRCC" and "chRCC":** |
| --- |
| miR-520b/miR-520c-3p |
| miR-617 |
| miR-3615 |
| miR-142-5p |
| miR-191-5p |
| miR-520c-3p |
| miR-514b-5p |
| miR-769-5p |
| miR-148b-5p |
| miR-375 |
| miR-587 |
| miR-193b-3p |
| miR-3164 |
| miR-3117-3p |
| miR-24-2-5p |
| miR-3144-5p |

| **Common elements in "papRCC" and "ccRCC":** | |
| --- | --- |
| miR-4293 |  |
| miR-3168 |  |
| miR-3663-3p |  |
| miR-494 |  |
| miR-4310 |  |

| **Common elements in "ccRCC" and "chRCC":** |
| --- |
| miR-3178 |

| **Elements only in "chRCC":** |
| --- |
| miR-1281 |
| miR-224-5p |
| miR-3917 |
| miRPlus-K1303* |
| miR-1247-3p |
| miR-2277-5p |
| miR-3124-5p |
| miR-483-5p |
| miR-3607-5p |
| miR-203 |
| miR-423-3p |
| miR-3196 |
| miR-3186-3p |
| miR-452-5p |
| miR-516a-5p |

| **Elements only in "ccRCC":** |
| --- |
| miR-551a |
| miR-422a |
| miR-940 |
| miR-34c-3p |
