# Supplementary material for: New miRNA Profiles Accurately Distinguish Renal Cell Carcinomas and Upper Tract Urothelial Carcinomas from the Normal Kidney
Source: PLoS One. 2014 Mar 12;9(3):e91646. doi: 10.1371/journal.pone.0091646 (PMC3951427; doi:10.1371/journal.pone.0091646)
Supplement: Table S4 — Reported chromosomal abnormalities associated with ccRCC, papRCC, chRCC and UT-UC, as well as some of the well-studied genes located within those aberrated regions that are involved in the pathogenesis of the tumour subtypes. Many of the significantly deregulated miRNAs are located within cytogenetic regions that are significantly aberrated in the different subtypes. In the reported chromosomal aberrations the minus sign denotes a loss and the plus sign denotes a gain in chromosomal material. Red colour (↑) denotes elevated miRNA expression and blue colour (↓) denotes reduced miRNA expression. (DOC) [file pone.0091646.s009.doc]

**Table S4**. Reported chromosomal abnormalities associated with ccRCC, papRCC, chRCC and UT-UC, as well as some of the well-studied genes located within those aberrated regions that are involved in the pathogenesis of the tumour subtypes. Many of the significantly deregulated miRNAs are located within cytogenetic regions that are significantly aberrated in the different subtypes. In the reported chromosomal aberrations the minus sign denotes a loss and the plus sign denotes a gain in chromosomal material. Red colour (↑) denotes elevated miRNA expression and blue colour (↓) denotes reduced miRNA expression.

| **RCC subtype or UT-UC** | **Frequency** | **Reported chromosomal aberrations** | **miRNAs within regions** | **Reported associated genes** |
| --- | --- | --- | --- | --- |
| **ccRCC** | 60-70% | 3p25-26, mutation or hypermethylation | **miR-4270 (↓)** | VHL, OGG1 |
|  |  | 3p14.2-p25 continuous deletion | **miRPlus-K1303* (↑), miR-2115-5p (↑), miR-1226-5p (↓), miR-191-5p (↓), miR-138-5p (↓), miR-1226-3p (↓), miR-711 (↑)** | VHL, FHIT, HCA1, OGG1 |
|  |  | 3p14.1 - | - |  |
|  |  | 3p14.2 - | - | FHIT |
|  |  | 3p12 - | **miR-4273 (↑), miR-1324 (↓), miR-885-5p (↑)** | NRC1 |
|  |  | 3p - | **miR-1226-3p (↓)** |  |
|  |  | 14q - (14q32.32 -qter) | **miR-656 (↓), miR-654-5p (↑), miR-1247-3p (↑), miR-203 (↑), miR-323a-3p (↑), miR-769-3p (↑), miR-409-3p (↑), miR-495 (↓), miR-376a-3p (↓), miR-543 (↓), miR-494 (↓), miR-127-3p (↓), miR-329 (↓), miR-1193 (↓), miR-342-3p (↓), miR-548y (↓), miR-409-5p (↓), miR-541-5p (↓), miR-496 (↓), miR-411-5p (↓), miR-323b-5p (↓), miR-379-3p (↓), miR-345-5p (↓), miR-411-3p (↓), miR-342-5p (↓), miR-1185-5p (↓), miR-136-5p (↓), miR-381 (↓), miR-410 (↓), miR-412 (↓), miR-369-3p (↓), miR-654-3p (↓), miR-376b (↓), miR-493-5p (↓), miR-154-5p (↓), miRPlus-J1003 (↓), miR-889 (↓), miR-134 (↓), miR-337-3p (↓), miR-3171 (↓), miR-4308 (↓), miR-624-3p (↓), miR-493-3p (↓)** |  |
|  |  | 8p23.3 - | **miR-596 (↓), miR-3674 (↓)** |  |
|  |  | 9q21.13-qter - | - |  |
|  |  | 9p - | **miR-876-5p (↓), miR-873-5p (↓), miR-31-3p (↓), miR-31-5p (↓), miR-876-3p (↓)** |  |
|  |  | 5q33.1 -qter + | **miR-103a-3p (↑), miR-146a-5p (↓)** |  |
|  |  | 7q11.22-q35 + | **miR-590-3p (↓), miR-4284 (↓)** |  |
|  |  | 16p12.3-p13.12 + | **miR-3180-3p (↓), miR-193b-5p (↑), miR-365a-5p/miR-365b-5p (↑), miR-3177-3p (↑), miR-3178 (↓), miR-940 (↓), miR-662 (↓), miR-3176 (↓), miR-193b-3p (↓)** |  |
|  |  | Loss of heterozygosity on chr 10q | **miR-3944-3p (↑), miR-2110 (↑), miR-548f (↑), miR-3663-3p (↓), miR-346 (↓), miR-146b-5p (↓), miR-3157-5p (↓), miR-607 (↓), miR-605 (↓), miR-3158-3p (↓), miR-548e (↓), miR-1307-5p (↓)** | PTEN/MAC |
| **papRCC** | 10-15% | 7q31 (c-met mutation) | - |  |
|  |  | 7 +/trisomy | **miR-591 (↓), miR-148a-5p (↓), miR-182-3p (↓), miR-153 (↓)** |  |
|  |  | 8 + | **miR-596 (↓), miR-875-5p (↓), miR-1207-3p (↑)** |  |
|  |  | 12q + | **miR-26a-2-3p (↑)** |  |
|  |  | 16q + | - |  |
|  |  | 17 +/trisomy | **miR-744-3p (↓), miR-3065-3p (↓), miR-3614-5p (↓)** | Fumarate hydratase |
|  |  | 20q + | - |  |
|  |  | 1p - | **miR-942 (↓), miR-4256 (↓), miR-137 (↓), miR-186-5p (↓), miR-1262 (↓), miR-101-3p (↓), miR-3117-3p (↓), miR-606 (↓), miR-30c-1-3p (↓), miR-34a-3p (↓), miR-4251 (↓), miR-429 (↓), miR-186-3p (↑), miR-3917 (↑), miR-551a (↑), miR-200b-5p (↑), miR-200a-3p (↑)** |  |
|  |  | 4q - | **miR-575 (↑), miR-302d-3p (↑), miR-548t-5p (↑), miR-548aa/miR-548t-3p (↓), miR-576-3p (↓), miR-577 (↓), miR-1973 (↓), miR-4276 (↓), miR-3945 (↓)** |  |
|  |  | 6q - | - |  |
|  |  | 9p - | **miR-876-5p (↑), miR-4290 (↑), miR-3074-3p (↑), miR-219-2-3p (↑), miR-873-5p (↓)** |  |
|  |  | 11p - | **miR-1256 (↓)** |  |
|  |  | 13q - | - |  |
|  |  | 14q - | - |  |
|  |  | 18 - | - |  |
|  |  | 21q - | - |  |
|  |  | X - | - |  |
|  |  | Y - | - |  |
| **chRCC** | 5% | Multiple losses of whole chromosomes: |  | p53 mutation |
|  |  | Y - | - |  |
|  |  | 1 | **miR-942 (↑), miR-186-5p (↑), miR-186-3p (↑), miR-551a (↑), miR-200b-5p (↑), miR-92b-5p (↑), miR-921 (↑), miR-214-3p (↑), miR-205-5p (↑), miR-4256 (↓), miR-137(↓), miR-1262(↓), miR-101-3p(↓), miR-3117-3p(↓), miR-606(↓), miR-30c-1-3p(↓), miR-30c-1-3p (↓), miR-3917(↓), miR-34a-3p(↓), miR-4251(↓), miR-200a-3p (↓), miR-429(↓), miR-554(↓), miR-181a-3p(↓), miR-3124-5p(↓)** |  |
|  |  | 2 | **miR-3126-5p (↑), miR-4262 (↑), miR-3127-5p (↑), miR-663b (↑), miR-10b-3p (↑), miR-3128 (↑), miR-3606 (↑), miR-562 (↑), miR-566 (↓), miR-558 (↓), miR-4266 (↓), miR-10b-5p (↓), miR-561-5p (↓), miR-375 (↓), miR-4269 (↓)** |  |
|  |  | 4 | **miR-218-5p (↓), miR-95 (↑), miR-571 (↑), miR-575 (↑), miR-548aa/miR-548t-3p (↓), miR-576-3p (↓), miR-302d-3p (↓), miR-577 (↓), miR-1973 (↓), miR-548t-5p (↓), miR-4276 (↓), miR-3945 (↓)** |  |
|  |  | 10 | **miR-3663-3p (↑)** |  |
|  |  | 13 | **miR-3168 (↑)** |  |
|  |  | 17 | **miR-1288 (↓), miR-3615 (↓)** |  |
|  |  | 21 | - |  |
|  |  | 17p11.2 (clinical manifestations of the Birt-Hogg-Dube syndrome) | **miR-1288 (↓)** |  |
| **UT-UC** | 26% | 2p21 - | - | MSH2 |
|  |  | 14q23.2 + | - | HIF-1α |
|  |  | 3q26 + | - | telomerase RNA component (hTR) |
|  |  | 16q22.1 - | **miR-140-5p (↓), miR-1538 (↓)** | E-cadherin |
